# Supplementary material for: Alchemical Design of Pharmacological Chaperones with Higher Affinity for Phenylalanine Hydroxylase
Source: Int J Mol Sci. 2022 Apr 19;23(9):4502. doi: 10.3390/ijms23094502 (PMC9100405; doi:10.3390/ijms23094502)
Supplement: Supplementary file 1 [file ijms-23-04502-s001.zip › ijms-1673817-supplementary.pdf]

Supplementary Material

Alchemical Design of Pharmacological Chaperones with Higher Affinity for Phenylalanine Hydroxylase

María Conde-Giménez[1,2], Juan José Galano-Frutos[1,2], María Galiana-Cameo[1,2#], Alejandro Mahía[1,2], Bruno L. Victor[3##], Sandra Salillas[1,2], Adrián Velázquez-Campoy[1,2,4,5], Rui M.M. Brito[3], José Antonio Gálvez[6], María D. Díaz-de-Villegas[6] and Javier Sancho[1,2,4*]

[1]Departamento de Bioquímica y Biología Molecular y Celular, Facultad de Ciencias, Universidad de Zaragoza, 50009 Zaragoza, Spain.

[2]Biocomputation and Complex Systems Physics Institute (BIFI)-GBsC-CSIC Joint Unit, Universidad de Zaragoza, 50018 Zaragoza, Spain.

[3]Coimbra Chemistry Center-Institute of Molecular Sciences (CQC-IMS), Department of Chemistry, University of Coimbra, 3004-535 Coimbra, Portugal.

[4]Aragon Health Research Institute (IIS Aragón), 50009 Zaragoza, Spain.

[5]CIBER de Enfermedades Hepáticas y Digestivas CIBERehd, Instituto de Salud Carlos III, 28029 Madrid, Spain.

[6]Instituto de Síntesis Química y Catálisis Homogénea (ISQCH), CSIC-Departamento de Química Orgánica, Facultad de Ciencias, Universidad de Zaragoza, 50009 Zaragoza, Spain.

[#]Present address: Instituto de Síntesis Química y Catálisis Homogénea (ISQCH)-Departamento de Química Inorgánica, Facultad de Ciencias, Universidad de Zaragoza, 50009 Zaragoza, Spain.

[##]Present address: BioISI, Biosystems and Integrative Sciences Institute, Faculty of Sciences, Universidade de Lisboa, 1749-016 Lisboa, Portugal.

* Correspondence: jsancho@unizar.es

Content

Supplementary Figures and Tables

Figure S1…………………………………………………………………… 3

Figure S2…………………………………………………………………… 4

Figure S3…………………………………………………………………… 5

Figure S4…………………………………………………………………… 6

Figure S5…………………………………………………………………… 7

Figure S6…………………………………………………………………… 8

Table S1……………………………………………………………………. 9

Table S2…………………………………………………………………… 11

Table S3…………………………………………………………………… 12

Table S4…………………………………………………………………… 13

Organic synthesis and characterization of intermediates and IV^{PC} analogues.….... 14

References………………………………………………………………… 34

NMR spectra……………………………………………………………… 35

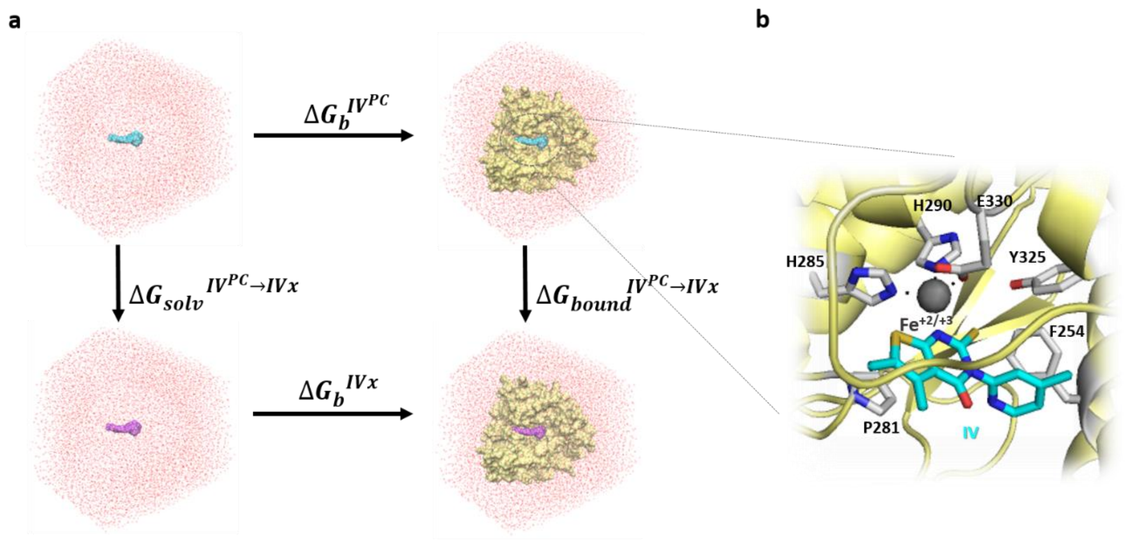

$$\Delta G_b{}^{IV^{PC}} + \Delta G_{bound}{}^{IV^{PC}\rightarrow IVx} - \Delta G_b{}^{IVx} - \Delta G_{solv}{}^{IV^{PC}\rightarrow IVx} = 0$$

$$\Delta\Delta G_b{}^{IV^{PC}\rightarrow IVx} = \Delta G_{bound}{}^{IV^{PC}\rightarrow IVx} - \Delta G_{solv}{}^{IV^{PC}\rightarrow IVx}$$

Figure S1. AFEC scheme and close view of the coordinated metal center. (**a**) Systems and simulated processes (vertical arrows) enabling the calculation of the relative alchemical binding free energy of the targeted **IVx** compounds versus $\mathbf{IV^{PC}}$ ($\Delta\Delta G_b$). (**b**) Modeled metal center showing the three amino acid residues (H285, H290 and E330, sticks with carbon atoms in gray) coordinating the iron atom (gray sphere) along with $\mathbf{IV^{PC}}$ (sticks with carbon atoms in cyan) in PDB 4ANP [1].

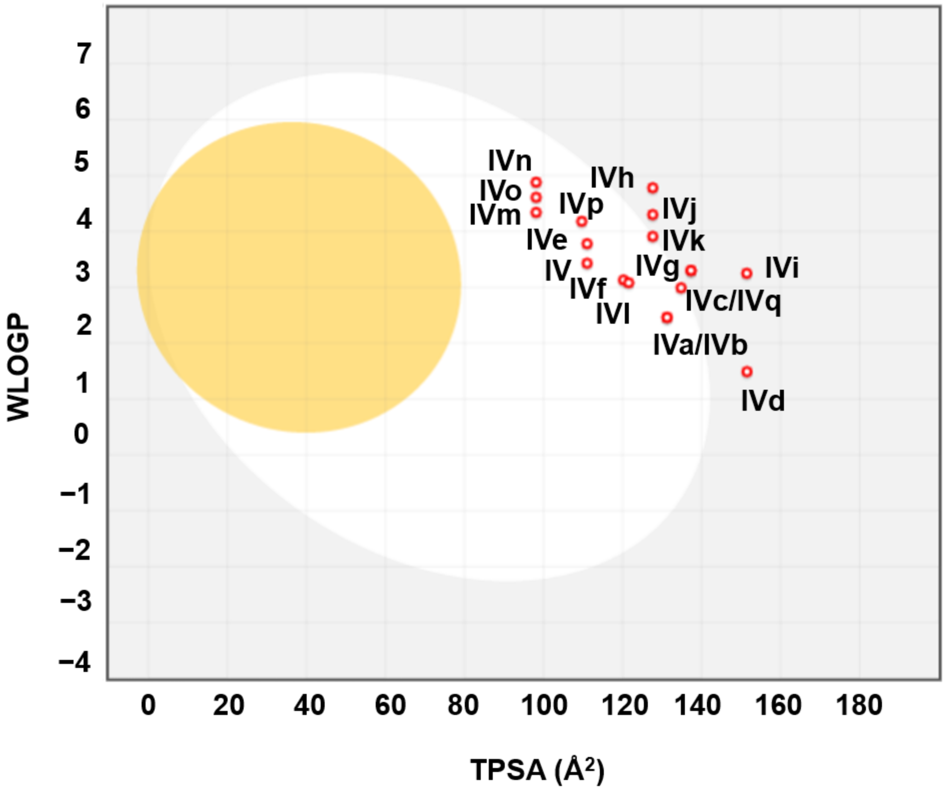

Figure S2. BOILED-Egg model [2] predictions for **IV[PC]** analogues. The lipophilicity (WLOGP descriptor) and polarity (TPSA descriptor) values obtained computationally using the SwissADME server (http://www.swissadme.ch) are represented. Compounds located within the white area are predicted to present a high intestinal absorption and those that are within the yolk area are predicted to present a high probability of crossing the blood-brain barrier.

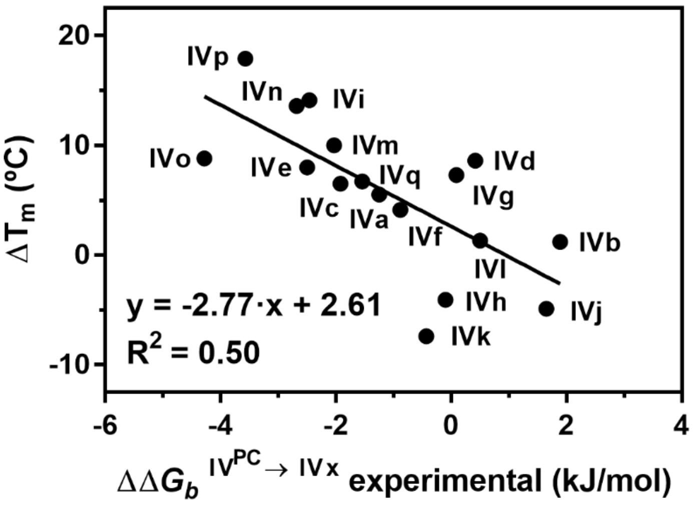

Figure S3. Correlation between experimental (by ITC) data of relative binding free energy ($\Delta\Delta G_{b\ IV^{PC}\rightarrow IVx}$) and thermal shift of PAH mid-point unfolding temperature (ΔT_m) in presence of **IVx** analogues. Scatter plot and linear fitting obtained from data of complexes of PAH with **IV^{PC}** and **IVx** analogues. The fitting equation and the square Pearson coefficient of the fit are depicted.

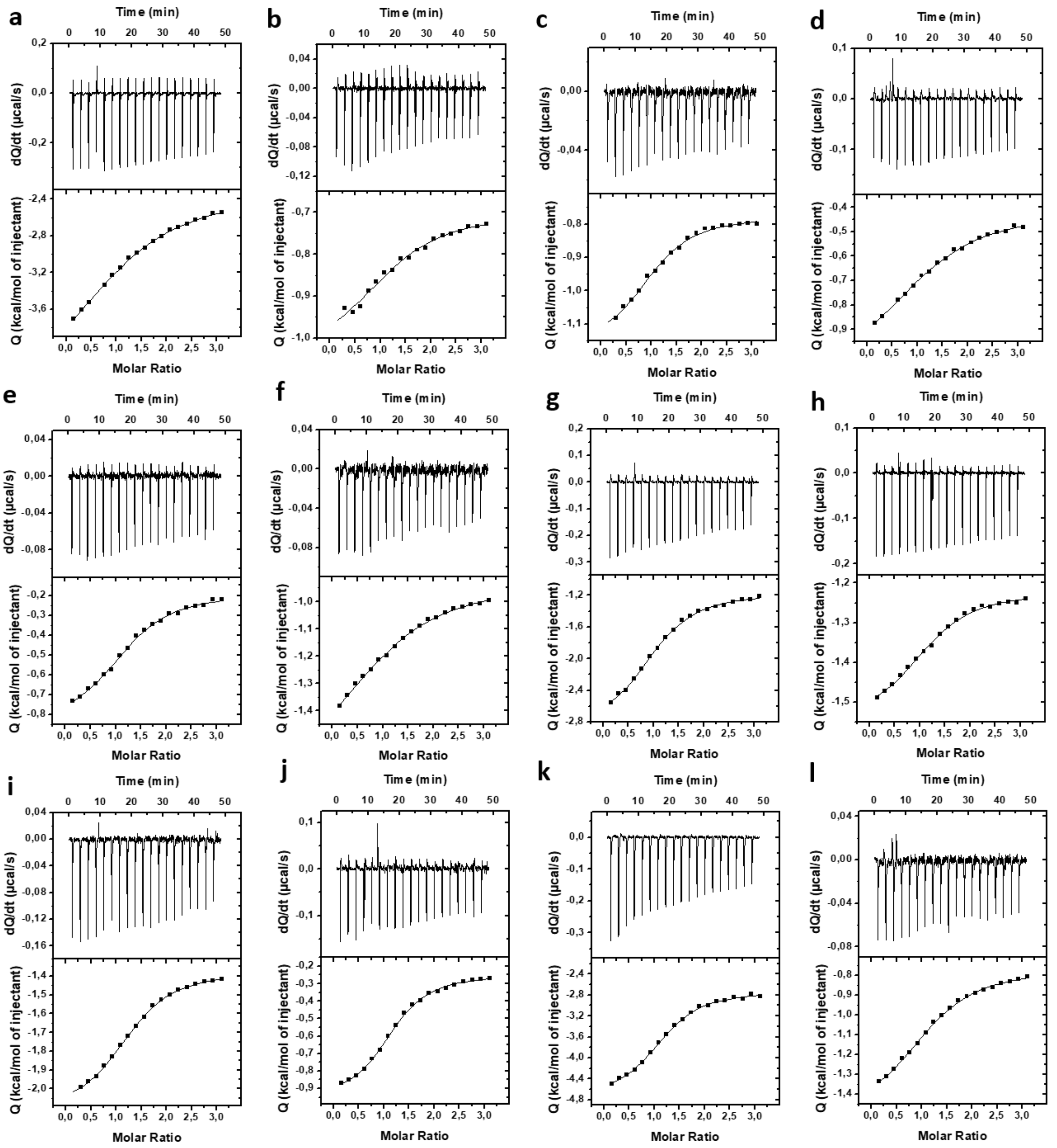

Figure S4. PAH titrations with **IV^{PC}** and **IVx** analogues showing a thermostabilizing effec**t.** Titrations were carried out with 20 µM PAH (in the calorimetric cell) and 300 µM of (**a**) **IV^{PC}**, (**b**) **IVa**, (**c**) **IVc**, (**d**) **IVd**, (**e**) **IVe**, (**f**) **IVg**, (**g**) **IVi**, (**h**) **IVm,** (**i**) **IVn,** (**j**) **IVo**, (**k**) **IVp** and (**l**) **IVq** (in the syringe) at 25 °C in PBS pH 7.4. The upper panels show the thermogram (thermal power vs. time) and the lower panels the binding isotherm (normalized heat vs. molar ratio) of different tests. The solid lines correspond to the fitting of the isotherms to a single site ligand binding model.

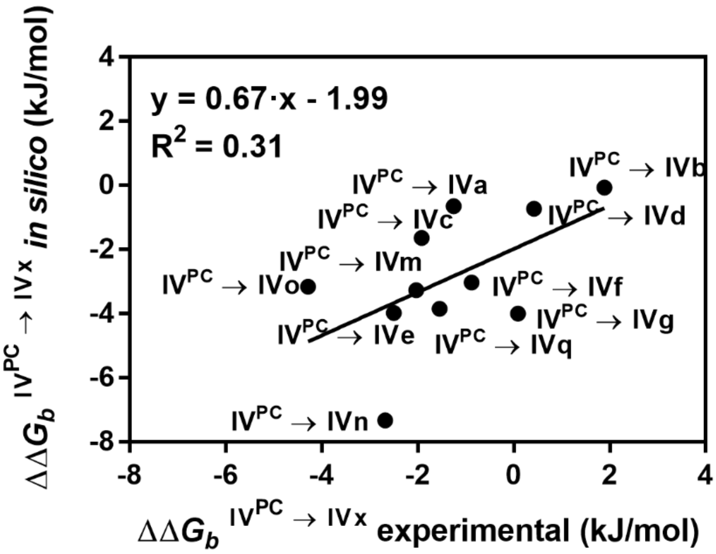

Figure S5. Correlation between computational (by AFEC) and experimental (by ITC) relative free-energies ($\Delta\Delta G_b{}^{\mathbf{IV^{PC}\rightarrow IVx}}$) for the binding of **IV**$^{\mathbf{PC}}$ and the **IVx** analogues to the enzyme PAH. The AFEC data has been obtained simulating the PAH enzyme as coordinating an FeIII ion. The straight line corresponds to a linear fit. The fitting equation and the square Pearson coefficient of the fit are indicated.

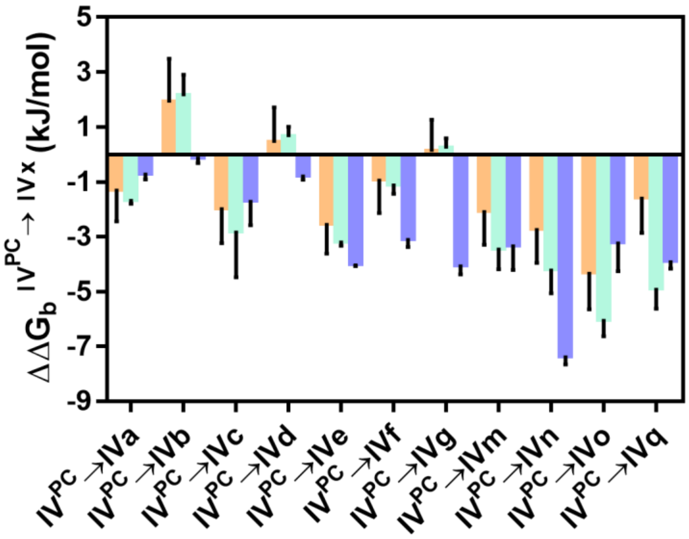

Figure S6. Bar diagram comparing the computational (by AFEC) and experimental (by ITC) relative binding free-energy values for the binding of compound IV^{PC} and analogues to the enzyme PAH. Experimental $\Delta\Delta G_b$ $^{\mathbf{IV^{PC}\rightarrow IVx}}$ values are shown in orange, while those calculated with either FeII or FeIII are shown in light green and light blue, respectively.

Table S1. Partial charges obtained for the atoms conforming the coordinated metal centers.[a]

Residue	Atom	Complex with Fe (II)	Complex with Fe (III)
His 285	N	−0.2741	−0.2957
	CA	−0.0880	−0.0504
	C	0.6778	0.6896
	O	−0.5699	−0.5656
	CB	−0.1016	−0.0556
	CG	−0.0188	0.0029
	ND1	−0.1007	−0.1377
	CD2	−0.1111	−0.0739
	CE1	−0.0483	0.0420
	NE2	−0.2233	−0.3870
	H	0.2015	0.2176
	HA	0.1014	0.0924
	HB2	0.0929	0.0881
	HB3	0.0929	0.0881
	HD1	0.3394	0.3433
	HE1	0.2098	0.1941
	HD2	0.1430	0.1414
	**Total fragment charge**	**0.3226**	**0.3336**
His 290	N	−0.4151	−0.0627
	CA	0.0058	−0.1360
	C	0.5893	0.6829
	O	−0.5086	−0.4607
	CB	−0.0216	−0.0279
	CG	0.0212	0.0243
	ND1	−0.0400	−0.0409
	CD2	−0.0511	0.0021
	CE1	−0.0484	−0.0054
	NE2	−0.3430	−0.4448
	H	0.2890	0.1388
	HA	0.0681	0.0846
	HB2	0.0239	0.0441

Table S1. Partial charges obtained for the atoms conforming the coordinated metal centers.[a]

Residue	Atom	Complex with Fe (II)	Complex with Fe (III)
	HB3	0.0239	0.0441
	HD1	0.3122	0.3163
	HE1	0.1839	0.1992
	HD2	0.1007	0.0782
	**Total fragment charge**	**0.1902**	**0.4361**
Glu330	N	−0.4133	−0.1531
	CA	−0.0390	−0.2408
	C	0.5642	0.8963
	O	−0.4888	−0.3015
	CB	−0.0086	0.0267
	CG	−0.0345	−0.0420
	CD	0.7511	0.8192
	OE1	−0.6168	−0.6556
	OE2	−0.6689	−0.7921
	H	0.2572	0.2312
	HA	0.0814	0.1165
	HB2	0.0297	0.0334
	HB3	0.0297	0.0334
	HG2	0.0087	0.0265
	HG3	0.0087	0.0265
	**Total fragment charge**	**−0.5392**	**0.0248**
Fe	FE	1.0263	1.2055
	**Total charge**	**1.0000**	**2.000**

[a] Merz-Kollman partial charges fitted by the RESP method as indicated in the Methods section of the main text.

Table S2. Lambda (λ) values settled for the AFEC transformations.[a]

λ	0	1	2	3	4	5	6	7	8	9	10	11	12	13	14	15
Bonded	0.0	0.0	0.0	0.0	0.0	0.0	0.1	0.2	0.3	0.4	0.5	0.6	0.7	0.8	0.9	1.0
Coul	0.0	0.2	0.4	0.6	0.8	1.0	1.0	1.0	1.0	1.0	1.0	1.0	1.0	1.0	1.0	1.0
vdW	0.0	0.0	0.0	0.0	0.0	0.0	0.08	0.17	0.27	0.38	0.5	0.61	0.72	0.83	0.92	1.0

[a] Bonded: λ values for the bonded interactions; Coul: λ values for the Coulombic interactions; vdW: λ values for the van de Waals interactions.

Table S3. Free energy changes for the solvated and bound AFEC transformations.[a]

Alchemical transformation	ΔG_{solv} (kJ/mol)	ΔG_{bound} Fe (II) (kJ/mol)	ΔG_{bound} Fe (III) (kJ/mol)
**IV^{PC}→IVa**	−13.93 ± 0.02	−15.54 ± 0.16	−14.58 ± 0.23
**IV^{PC}→IVb**	69.16 ± 0.01	71.28 ± 0.73	69.08 ± 0.22
**IV^{PC}→IVc**	48.26 ± 0.06	45.48 ± 1.64	46.61 ± 0.87
**IV^{PC}→IVd**	73.55 ± 0.03	74.18 ± 0.28	72.82 ± 0.16
**IV^{PC}→IVe**	75.27 ± 0.01	72.13 ± 0.18	71.29 ± 0.08
**IV^{PC}→IVf**	32.71 ± 0.01	33.78 ± 0.36	35.76 ± 0.32
**IV^{PC}→IVg**	44.30 ± 0.01	44.51 ± 0.36	40.29 ± 0.36
**IV^{PC}→IVi**	152.17 ± 0.12	152.81 ± 3.09	129.23 ± 4.24
**IV^{PC}→IVj**	205.72 ± 0.10	n.c.[b]	196.21 ± 5.85
**IV^{PC}→IVk**	227.14 ± 0.15	230.15 ± 0.47	n.c.[b]
**IV^{PC}→IVl**	231.07 ± 0.03	239.43 ± 2.61	n.c.[b]
**IV^{PC}→IVm**	197.51 ± 0.03	194.12 ± 0.77	194.23 ± 0.90
**IV^{PC}→IVn**	186.15 ± 0.01	181.98 ± 0.88	178.82 ± 0.31
**IV^{PC}→IVo**	158.40 ± 0.03	152.42 ± 0.61	155.23 ± 1.06
**IV^{PC}→IVp**	376.81± 0.11	375.82 ± 2.76	364.97 ± 2.91
**IV^{PC}→IVq**	−19.59 ± 0.22	−24.45 ± 0.54	−23.45 ± 0.09

[a] Free-energy changes used for the calculation of relative binding free-energies along with errors displayed in **Table 2** of the main text. The values are the mean of at least three MD simulation replicas and SE the standard error.

[b] Abbreviation n.c. stands for 'non-calculated' free-energy. In such cases, the compound (**IVx**) did not retain the binding in the coordinated center in any of the simulated replicas so that calculating the binding free energy was meaningless.

Table S4. Calculated SwissADME [3] properties for compound IV^{PC} analogues. Physicochemical properties; lipophilicity parameter (consensus of all five predicted octanol/water partition coefficient reported in [3]); theoretical solubility (from topological method implemented by [4]): pharmacokinetic properties for oral administration (gastrointestinal absorption according to the white of the BOILED-Egg [2]) and the compliance or violation (in this case, indicating the number) of different rules for the suitability of a chemical compound as a potential drug.

	**IV^{PC}**	**IVa**	**IVb**	**IVc**	**IVd**	**IVe**	**IVf**	**IVg**	**IVh**	**IVi**	**IVj**	**IVk**	**IVl**	**IVm**	**IVn**	**IVo**	**IVp**	**IVq**
**Physicochemical properties**																		
**Num. heavy atoms**	20	21	21	24	22	20	21	21	31	27	28	27	24	21	23	23	26	24
**Num. arom. heavy atoms**	15	15	15	15	15	15	15	15	21	15	15	15	15	15	19	15	15	15
**Num. H-bonds acceptors**	2	3	3	4	4	2	3	3	5	6	5	5	4	1	1	1	2	4
**Num. H-bond donors**	1	2	2	1	3	1	1	1	0	0	0	0	1	1	1	1	1	1
**TPSA (Å^2) [5]**	111	131	131	137	151	111	120	135	128	151	128	127	122	98	98	98	110	137
**Lipophilicity**																		
**Consensus Log $P_{o/w}$**	3.41	2.63	2.59	3.44	1.85	3.59	3.08	2.83	4.72	3.34	4.41	4.09	3.27	4.40	4.62	4.75	4.09	3.35
**Water solubility**																		
**Solubility (mg/ml) × 10^4**	183	1160	1160	149	7330	98.6	334	436	3.59	83.4	9.82	27.5	224	34.1	10.4	11.0	5.53	228
**Pharmacokinetics**																		
**Gastrointestinal absorption**	High	High	High	Low	Low	High	High	High	Low	Low	Low	High	High	High	High	High	High	Low
**Drug-likeness**																		
**Lipinski´s rule [6]**	Yes	Yes	Yes	Yes	Yes	Yes	Yes	Yes	Yes	Yes	Yes	Yes	Yes	Yes	Yes	Yes	Yes	Yes
**Veber´s rule [7]**	Yes	Yes	Yes	Yes	No,1	Yes	Yes	Yes	Yes	Yes	Yes	Yes	Yes	Yes	Yes	Yes	Yes	Yes
**Egan´s rule [8]**	Yes	Yes	Yes	No,1	No,1	Yes	Yes	No,1	Yes	Yes	Yes	Yes	Yes	Yes	Yes	Yes	Yes	No,1

Organic synthesis and characterization of intermediates and compound IV analogues

Reactions were carried out using anhydrous solvents provided by a solvent purification system. Reactions were magnetically stirred and whenever possible monitored by TLC. TLC was performed on precoated silica gel polyester plates and products were visualized using ultraviolet light (254 nm), ninhydrin, potassium permanganate or phosphomolybdic acid solution followed by heating. Column chromatography was performed using silica gel (Kiesegel 60, 230−400 mesh). Melting points were determined in open glass capillaries with a Gallenkamp apparatus and are uncorrected. ^{1}H-NMR and ^{13}C-NMR spectra were acquired at room temperature at 400 and 100 MHz, respectively, using a 5 mm probe with a Bruker AV400 spectrometer. Chemical shifts (δ) are reported in parts per million relative to tetramethylsilane with the solvent resonance as the internal standard. Coupling constants (J) are quoted in Hertz. The splitting patterns are reported as 's' (singlet), 'd' (doublet), 't' (triplet), 'q' (quartet), 'dd' (doublet of doublets), 'm' (multiplet), 'br s' (broad singlet) and 'br d' (broad doublet). Attached proton test spectra were acquired to determine the types of carbon signals. High-resolution mass spectra were made using a Bruker Daltonics micro-TOF (time-of-flight) spectrometer, and spectra were recorded from methanolic ultradiluted solutions using the positive electrospray ionization mode (ESI^{+}).

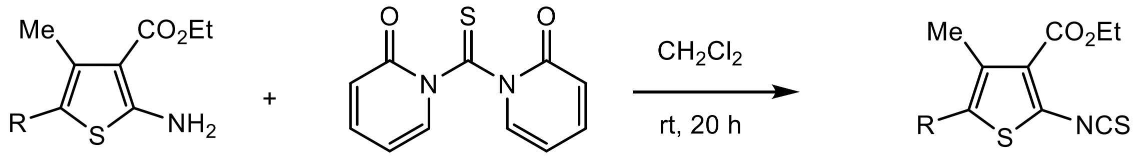

1, R = CO_2Et
2, R = Me

3, R = CO_2Et
4, R = Me

Diethyl 5-isothiocyanato-3-methylthiophene-2,4-dicarboxylate (3)

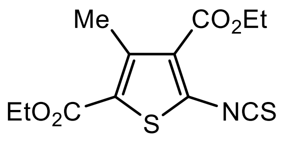

To a solution of diethyl 5-amino-3-methylthiophene-2,4-dicarboxylate (**1**) (386 mg, 1.5 mmol) in dry dichloromethane (25 mL), 1,1'-thiocarbonylbis(pyridin-2(1*H*)-one) (348 mg, 1.5 mmol) was added and the resulting solution was stirred at room temperature for 20 hours. Then solvent was removed under reduced pressure and the resulting residue was purified by column chromatography (Eluent, hexane/ Et_2O 9:1) to afford 310 mg (69% yield) of compound **3** as a white solid. M. p. = 69.6−69.8 ºC. ^{1}H-

NMR (400 MHz, $CDCl_3$): δ 4.39 (q, *J* = 7.1 Hz, 2H, $CO_2CH_2CH_3$), 4.32 (q, *J* = 7.1 Hz, 2H, $CO_2CH_2CH_3$), 2.71 (s, 3H, CH_3), 1.42 (t, *J* = 7.1 Hz, 3H, $CO_2CH_2CH_3$), 1.36 (t, *J* = 7.1 Hz, 3H, $CO_2CH_2CH_3$). $^{13}C\{^1H\}$-APT NMR (100 MHz, $CDCl_3$): δ 161.7 (C), 161.4 (C), 146.1 (C), 139.4 (C), 138.9 (C), 128.8 (C), 121.9 (C), 61.4 (CH_2), 61.4 (CH_2), 15.2 (CH_3), 14.4 (CH_3), 14.3 (CH_3). HRMS (ESI^+): m/z $[M + Na]^+$ calculated for $C_{12}H_{13}NNaO_4S_2$ 322.0178, found 322.0171.

Ethyl 2-isothiocyanato-4,5-dimethylthiophene-3-carboxylate (4)

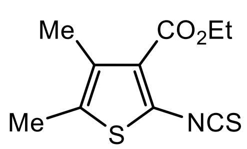

To a solution of ethyl 2-amino-4,5-dimethylthiophene-3-carboxylate (**2**) (150 mg, 0.75 mmol) in dry dichloromethane (10 mL), 1,1'-thiocarbonylbis(pyridin-2(1*H*)-one) (175 mg, 0.75 mmol) was added and the resulting solution was stirred at room temperature for 2 hours. Then solvent was removed under reduced pressure and the resulting residue was purified by column chromatography (Eluent, CH_2Cl_2) to afford 154 mg (85% yield) of compound **4** as a white solid. ^1H-NMR (400 MHz, $CDCl_3$): δ 4.29 (q, *J* = 7.1 Hz, 2H, $CO_2CH_2CH_3$), 2.22 (s, 3H, CH_3), 2.18 (s, 3H, CH_3), 1.34 (t, *J* = 7.1 Hz, 3H, $CO_2CH_2CH_3$). $^{13}C\{^1H\}$-APT NMR (100 MHz, $CDCl_3$): δ 162.3 (C), 137.0 (C), 132.9 (C), 131.5 (C), 128.8 (C), 127.8 (C), 60.9 (CH_2), 14.4 (CH_3), 13.8 (CH_3), 13.0 (CH_3). HRMS (ESI^+): m/z $[M + Na]^+$ calculated for $C_{10}H_{11}NNaO_2S_2$ 264.0123, found 264.0119.

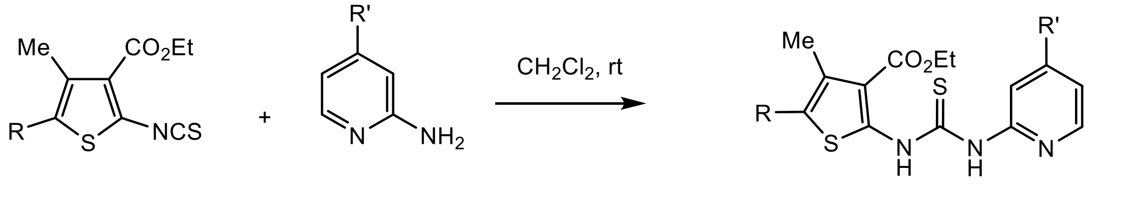

3, R = CO_2Et
4, R = Me
5, R = Me
6, R = $CH_2OTBDMS$
7, R = CO_2Et, R' = Me
8, R = CO_2Et, R' = $CH_2OTBDMS$
9, R = Me, R' = $CH_2OTBDMS$

General procedure for the synthesis of (pyridin-2-yl)thioureidothiophene carboxylates (7−9)

To a solution of the corresponding isothiocyanate (1.0 mmol) in dry dichloromethane (25 mL), the corresponding pyridin-2-amine (2.0 mmol) was added and the resulting solution was stirred at room temperature until TLC analysis in hexane/EtOAc 8:2 shows almost complete disappearance of isothiocyanate (24-48 h). Then solvent was removed under reduced pressure and the resulting residue was purified by column chromatography to

afford the corresponding 3-(pyridin-2-yl)-2-thioxo-2,3-dihydrothieno[2,3-d]pyrimidin-4(1H)-one.

Diethyl 3-methyl-5-(3-(4-methylpyridin-2-yl)thioureido)thiophene-2,4-dicarboxylate (7)

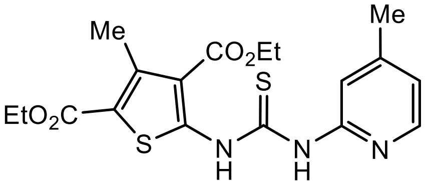

Starting from diethyl 5-isothiocyanato-3-methylthiophene-2,4-dicarboxylate (**3**) (300 mg, 1.0 mmol) and 4-methylpyridin-2-amine (**5**) (216 mg, 2.0 mmol) and following general procedure 398 mg (98% yield) of compound **7** were obtained as a yellow solid (Eluent column chromatography,1st Et_2O/hexane 1:1, 2nd CH_2Cl_2/Et_2O 7:3). M. p. = 200.5−200.8 ºC. ^{1}H-NMR (400 MHz, $CDCl_3$): δ 15.86 (s, 1H, N*H*), 8.58 (br s, 1H, N*H*), 8.29 (d, *J* = 5.2 Hz, 1H, H_6-py), 6.88 (d, *J* = 5.2 Hz, 1H, H_5-py), 6.61 (br s, 1H, H_3-py), 4.42 (q, *J* = 7.1 Hz, 2H, CO_2-C*H*$_2$CH$_3$), 4.33 (q, = 7.1 Hz, 2H, CO_2C*H*$_2$CH$_3$), 2.75 (s, 3H, CH_3), 2.35 (s, 3H, CH_3), 1.41 (t, *J* = 7.1 Hz, 3H, CO_2-CH$_2$C*H*$_3$), 1.37 (t, *J* = 7.1 Hz, 3H, CO_2CH$_2$C*H*$_3$). ^{13}C{^{1}H}-APT NMR (100 MHz, $CDCl_3$): δ 175.9 (C), 165.1 (C), 163.4 (C), 153.2 (C), 151.7 (C), 150.8 (C), 145.8 (CH), 144.1 (C), 120.3 (CH), 118.6 (C), 117.7 (C), 111.5 (CH), 60.8 (CH_2), 60.6 (CH_2), 21.3 (CH_3), 15.6 (CH_3), 14.5 (CH_3), 14.3 (CH_3). HRMS (ESI$^+$): m/z $[M + H]^+$ calculated for $C_{18}H_{22}N_3O_4S_2$ 408.1056, found 408.1051.

Diethyl 5-(3-(4-(((*tert*-butyldimethylsilyl)oxy)methyl)pyridin-2-yl)thioureido)-3-methylthiophene-2,4-dicarboxylate (8)

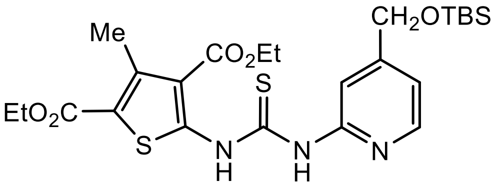

Starting from diethyl 5-isothiocyanato-3-methylthiophene-2,4-dicarboxylate (**3**) (120 mg, 0.40 mmol) and 4-(((tert-butyldimethylsilyl)oxy)methyl)pyridin-2-amine (**6**) (191 mg, 0.80 mmol) and following general procedure 197 mg (92% yield) of compound **8** were obtained as a yellow solid (Eluent column chromatography, CH_2Cl_2). M. p. = 157.0−157.2 ºC. ^{1}H-NMR (400 MHz, $CDCl_3$): δ 15.87 (s, 1H, N*H*), 8.41(s, 1H, N*H*), 8.37 (d, *J* = 5.3 Hz, 1H, $H_{6\text{-py}}$), 6.97 (d, *J* = 5.3 Hz, $H_{5\text{-py}}$) 6.77 (s, 1H, $H_{3\text{-py}}$), 4.73 (s, 2H, C*H*$_2$OTBS), 4.43 (q, *J* = 7.1 Hz, 2H, CO_2C*H*$_2$CH$_3$), 4.33 (q, *J* = 7,1 Hz, 2H, CO_2C*H*$_2$CH$_3$), 2.76 (s, 3H, C*H*$_3$), 1.41 (t, *J* = 7,1 Hz, 3H, CO_2CH$_2$C*H*$_3$), 1.37 (t, *J* = 7,1 Hz, 3H, CO_2CH$_2$C*H*$_3$), 0.96 (s, 9H, (C*H*$_3$)$_3$C-Si), 0.12 (s, 6H, (C*H*$_3$)$_2$-Si).^{13}C{^{1}H}-APT (100 MHz, $CDCl_3$): δ 175.9 (C), 165.1 (C), 163.3 (C), 154.6 (C), 153.2 (C), 151.8 (C), 145.9 (CH), 144.1 (C), 118.6 (C), 117.7 (C), 116.0 (CH), 107.6 (CH), 63.3 (CH_2), 60.6

(CH_2), 60.6 (CH_2), 25.9 (CH_3), 18.4 (C), 15.6 (CH_3), 14.5 (CH_3), 14.3 (CH_3), -5.4 (CH_3). HRMS (ESI$^+$): m/z [M + H]$^+$ calculated for $C_{24}H_{36}N_3O_5S_2Si$ 538.1860, found 538.1849.

Ethyl 2-(3-(4-(((*tert*-butyldimethylsilyl)oxy)methyl)pyridin-2-yl)thioureido)-4,5-dimethylthiophene-3-carboxylate (9)

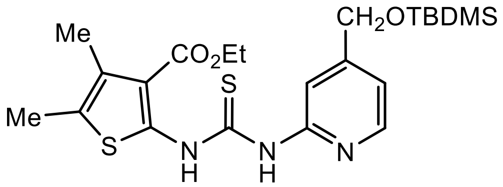

Starting from ethyl 2-isothiocyanato-4,5-dimethylthiophene-3-carboxylate (**4**) (96.5 mg, 0.40 mmol) and 4-(((tert-butyldimethylsilyl)oxy)methyl)pyridin-2-amine (**6**) (191 mg, 0.80 mmol) and following general procedure 173 mg (90% yield) of compound **9** were obtained as a yellow solid (Eluent column chromatography, CH_2Cl_2). M. p. = 172.7−173.0 ºC. ^{1}H-NMR (400 MHz, $CDCl_3$): δ 15.56 (s, 1H, N*H*), 8.35 (d, *J* = 5.3 Hz, 1H, H_6-py), 8.33 (s, 1H, N*H*), 6.94 (d, *J* = 5.3 Hz, 1H, H_5-py), 6.74 (br s, 1H, H_3-py), 4.72 (s, 2H, CH_2OTBS), 4.39 (q, *J* = 7.1 Hz, 2H, $CO_2CH_2CH_3$), 2.28 (s, 3H, CH_3), 2.26 (s, 3H, CH_3), 1.38 (t, *J* = 7.1 Hz, 3H, $CO_2CH_2CH_3$), 0.95 (s, 9H, $(CH_3)_3$C-Si), 0.12 (s, 6H, $(CH_3)_2$-Si). ^{13}C{^{1}H}-APT (100 MHz, $CDCl_3$): δ 174.8 (C), 165.2 (C), 154.3 (C), 152.1 (C), 146.7 (C), 145.9 (CH), 129.4 (C), 124.7 (C), 115.7 (CH), 107.6 (CH), 63.3 (CH_2), 60.3 (CH_2), 25.9 (CH_3), 18.4 (C), 14.5 (CH_3), 14.4 (CH_3), 12.5 (CH_3), -5,4 (CH_3). HRMS (ESI$^+$): m/z [M + H]$^+$ calculated for $C_{22}H_{34}N_3O_3S_2Si$ 480.1805, found 480.1820.

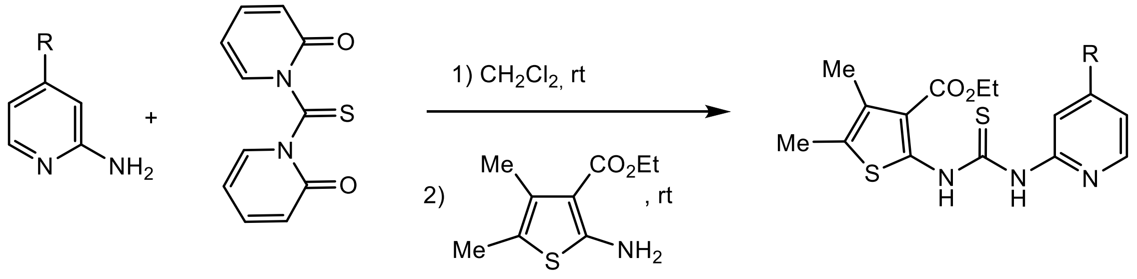

10, R = Cl
11, R = OMe
12, R = CN

2

13, R = Cl
14, R = OMe
15, R = CN

General procedure for the synthesis of (pyridin-2-yl)thioureidothiophene carboxylates (13−15)

To a solution of 1,1'-thiocarbonylbis(pyridin-2(1*H*)-one) (0.80 mmol) in dry dichloromethane (20 mL), the corresponding pyridin-2-amine (0.90 mmol) was added and the resulting solution was stirred at room temperature until TLC analysis in

hexane/EtOAc 1:1 shows almost complete disappearance of of 1,1'-thiocarbonylbis(pyridin-2(1*H*)-one) (2-10 h). Then ethyl 2-amino-4,5-dimethylthiophene-3-carboxylate (**2**) (0.70 mmol) was added and the resulting solution was stirred at room temperature until TLC analysis in hexane/EtOAc 8:2 shows almost complete disappearance of thiophene (20-40 h). Then solvent was removed under reduced pressure and the resulting residue was purified by column chromatography to afford the corresponding 3-(pyridin-2-yl)-2-thioxo-2,3-dihydrothieno[2,3-d]pyrimidin-4(1H)-one.

Ethyl 2-(3-(4-chloropyridin-2-yl)thioureido)-4,5-dimethylthiophene-3-carboxylate (13)

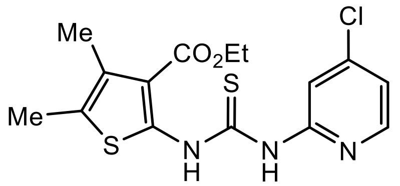

Starting from 4-chloropyridin-2-amine (**10**) (116 mg, 0.90 mmol) and following general procedure 239 mg (92% yield) of compound **13** were obtained as a yellow solid (Eluent column chromatography, CH_2Cl_2). M. p. = 208.8−209.1 ºC. ^{1}H-NMR (400 MHz, DMSO-d_6): δ 15.38 (s, 1H, N*H*), 11.27 (s, 1H, N*H*), 8.31 (d, *J* = 5.6 Hz, 1H, H_6-py), 7.27 (d, *J* = 1.6 Hz, 1H, H_3-py), 7.25 (dd, *J* = 5.5, 1.6 Hz, 1H, H_5-py), 4.33 (q, *J*= 7.1 Hz, 2H, $CO_2CH_2CH_3$), 2.24 (s, 3H, *CH3*), 2.20 (s, 3H, *CH3*), 1.30 (t, *J* = 7.1 Hz, 3H, $CO_2CH_2CH_3$). ^{13}C{^{1}H}-APT NMR (100 MHz, DMSO-d_6): δ 174.4 (C), 164.3 (C), 153.0 (C), 147.2 (CH), 145.5 (C), 144.6 (C), 128.6 (C), 124.1 (C), 118.6 (CH), 116.7 (C), 111.6 (CH), 60.2 (CH_2), 14.1 (CH_3), 14.1 (CH_3), 12.0 (CH_3). HRMS (ESI$^+$): m/z [M + Na]$^+$ calculated for $C_{15}H_{16}ClN_3NaO_2S_2$ 392.0265, found 392.0271.

Ethyl 2-(3-(4-methoxypyridin-2-yl)thioureido)-4,5-dimethylthiophene-3-carboxylate (14)

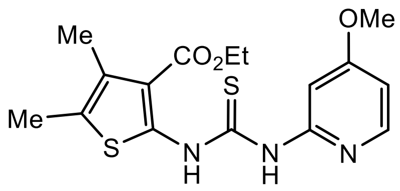

Starting from 4-methoxypyridin-2-amine (**11**) (116 mg, 0.75 mmol) and following general procedure 164 mg (85% yield) of compound **14** were obtained as a yellow solid (Eluent column chromatography, 1st CH_2Cl_2, 2nd hexane/EtOAc 2:1). M. p. = 183.2–183.5 ºC. ^{1}H-NMR (400 MHz, DMSO-d_6) δ 15.66 (s, 1H, N*H*), 11.01 (s, 1H, N*H*), 8.16 (d, *J* = 6.7 Hz, 1H, H_6-py), 6.79–6.73 (m, 2H, H_5-py, H_3-py), 4.33 (q, *J* = 7.1 Hz, 2H, $CO_2CH_2CH_3$), 3.82 (s, 3H, OCH_3), 2.23 (s, 3H, CH_3), 2.20 (s, 3H, CH_3), 1.29 (t, *J* = 7.1 Hz, 3H, $CO_2CH_2CH_3$). ^{13}C{^{1}H}-APT NMR (100 MHz, DMSO-d_6): δ 174.7 (C), 167.1 (C), 164.2 (C), 153.9 (C), 146.7 (CH), 145.6 (C), 128.5

(C), 124.0 (C), 116.8 (C), 106.6 (CH), 96.2 (CH), 60.1 (CH_2), 55.5 (CH_3), 14.1 (CH_3), 14.0 (CH_3), 12.1 (CH_3). HRMS (ESI^+): m/z $[M + Na]^+$ calculated for $C_{16}H_{19}N_3NaO_3S_2$ 388.0760, found 388.0769.

Ethyl 2-(3-(4-cyanopyridin-2-yl)thioureido)-4,5-dimethylthiophene-3-carboxylate (15)

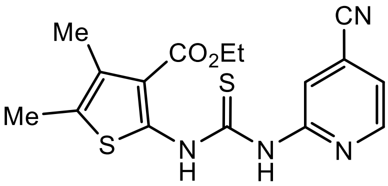

Starting from 4-cyanopyridin-2-amine (**12**) (89 mg, 0.75 mmol) and following general procedure 167 mg (88% yield) of compound **15** were obtained as a yellow solid (Eluent column chromatography, 1st CH_2Cl_2, 2nd hexane/EtOAc 1:1). M. p. = 215.4–215.7 ºC. ^{1}H-NMR (400 MHz, DMSO-d_6) δ 15.24 (s, 1H, N*H*), 11.48 (s, 1H, N*H*), 8.54 (d, J = 5.5 Hz, 1H, H_6-py), 7.54 (d, J = 5.5 Hz, 1H, H_5-py), 7.51 (s, 1H, H_3-py), 4.35 (q, J = 7.1 Hz, 2H, $CO_2CH_2CH_3$), 2.25 (s, 3H, CH_3), 2.21 (s, 3H, CH_3), 1.31 (t, J = 7.1 Hz, 3H, $CO_2CH_2CH_3$). $^{13}C\{^1H\}$-APT NMR (100 MHz, DMSO-d_6): δ 174.2 (C), 164.4 (C), 152.3 (C), 147.3 (CH), 145.5 (C), 128.7 (C), 124.3 (C), 121.6 (C), 119.3 (CH), 116.7 (C), 116.5 (C), 114.9 (CH), 60.3 (CH_2), 14.1 (CH_3), 14.1 (CH_3), 12.1 (CH_3). HRMS (ESI^+): m/z $[M + Na]^+$ calculated for $C_{16}H_{16}N_4NaO_2S_2$ 383.0607, found 383.0603.

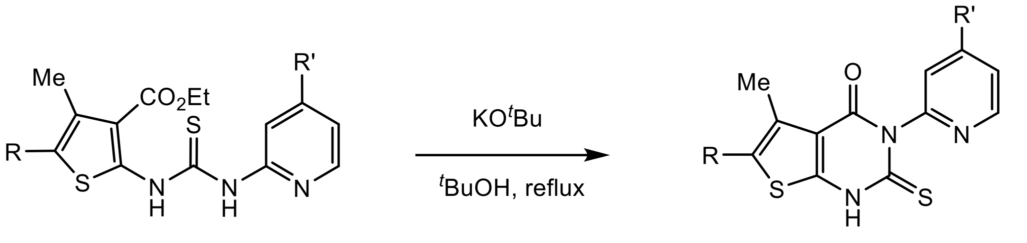

7, R = CO_2Et, R' = Me
8, R = CO_2Et, R' = $CH_2OTBDMS$
9, R = Me, R' = $CH_2OTBDMS$
13, R = Me, R' = Cl
14, R = Me, R' = OMe
15, R = Me, R' = CN

IVc, R = CO_2Et, R' = Me
16, R = CO_2Et, R' = $CH_2OTBDMS$
17, R = Me, R' = $CH_2OTBDMS$
IVe, R = Me, R' = Cl
IVf, R = Me, R' = OMe
IVg, R = Me, R' = CN

General procedure for the synthesis of 3-(pyridin-2-yl)-2-thioxo-2,3-dihydrothieno[2,3-d]pyrimidin-4(1H)-ones

To a solution of the corresponding (pyridin-2-yl)thioureidothiophene carboxylate (0.5 mmol) in *tert*-butanol (5 mL), potassium *tert*-butoxide (56 mg, 0.5 mmol) was added and the resulting mixture was stirred under reflux conditions for 2–15 hours. The reaction mixture was cooled to room temperature and evaporated under reduced pressure. The

resulting residue was dissolved in water and the solution was neutralized with 1M HCl aqueous solution. The obtained solid was filtered, washed with cool water and dried to afford the corresponding 3-(pyridin-2-yl)-2-thioxo-2,3-dihydrothieno[2,3-d]pyrimidin-4(1H)-one.

Ethyl 5-methyl-3-(4-methylpyridin-2-yl)-4-oxo-2-thioxo-1,2,3,4-tetrahydrothieno[2,3-d]pyrimidine-6-carboxylate (IVc)

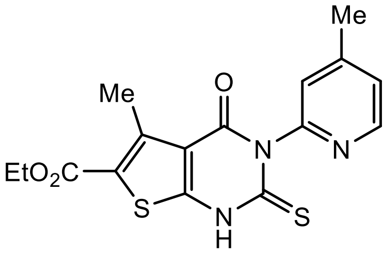

Upon heating of (pyridin-2-yl)thioureidothiophene carboxylate (**7**) (140 mg, 0.34 mmol) for 15 hours and following general procedure 78 mg (63% yield) of compound **IVc** were obtained as a yellowish solid. M. p. = 183–184 ºC. ^{1}H-NMR (400 MHz, DMSO-*d*$_6$): δ 13.95 (br s, 1H, N*H*), 8.38 (d, *J* = 5.1 Hz, 1H, H$_6$-py), 7.25 (d, *J* = 5.1 Hz, 1H, H$_5$-py), 7.23 (s, 1H, H$_3$-py), 4.24 (q, *J* = 7.3 Hz, 3H, CO$_2$C*H*$_2$CH$_3$), 2.64 (s, 3H, C*H*$_3$), 2.32 (s, 3H, C*H*$_3$), 1.25 (t, *J* = 7.3 Hz, 3H, CO$_2$CH$_2$C*H*$_3$). ^{13}C{^{1}H} NMR (100 MHz, DMSO-*d*$_6$): δ 175.0 (C), 161.7 (C), 157.0 (C), 153.5 (C), 151.6 (C), 149.6 (CH), 148.9 (C), 144.1 (C), 125.0 (CH), 124.7 (CH), 118.2 (C), 117.2 (C), 61.2 (CH$_2$), 20.3 (CH$_3$), 14.2 (CH$_3$), 14.1 (CH$_3$). HRMS (ESI$^+$): m/z [M + Na]$^+$ calculated for $C_{16}H_{15}N_3NaO_3S_2$ 384.0447, found 384.0441.

Ethyl 3-(4-(((*tert*-butyldimethylsilyl)oxy)methyl)pyridin-2-yl)-5-methyl-4-oxo-2-thioxo-1,2,3,4-tetrahydrothieno[2,3-d]pyrimidine-6-carboxylate (16)

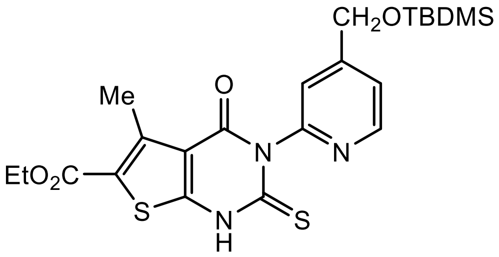

Upon heating of (pyridin-2-yl)thioureidothiophene carboxylate (**8**) (175 mg, 0.33 mmol) for 6 hours and following general procedure 154 mg (95% yield) of compound **16** were obtained as a yellowish solid. M. p. > 300 ºC . ^{1}H-NMR (400 MHz, DMSO-*d*$_6$): δ 8.41 (d, *J* = 5.1 Hz, 1H, H$_6$-py), 7.22 (d, *J* = 5.1 Hz, 1H, H$_5$-py), 7.06 (s, 1H, H$_3$-py), 4.77 (s, 2H, C*H*$_2$OTBS), 4.23 (q, *J* = 7.1 Hz, 2H, CO$_2$C*H*$_2$CH$_3$), 2.68 (s, 3H, CH$_3$), 1.28 (t, *J* = 7.1 Hz, 3H, CO$_2$CH$_2$C*H*$_3$), 0.92 (s, 9H, (C*H*$_3$)$_3$C-Si), 0.10 (s, 6H, (C*H*$_3$)$_2$-Si). ^{13}C{^{1}H}-APT NMR (100 MHz, DMSO-d$_6$, 298 K): δ 178.6 (C), 168.3 (C), 162.7 (C), 160.2 (C), 154.7 (C), 151.7 (C), 148.4 (CH), 144.8 (C), 121.5 (CH), 119.4 (CH), 116.1 (C), 113.3 (C), 62.8 (CH$_2$), 60.2

(CH_2), 25.8 (CH_3), 18.0 (C), 14.8 (CH_3), 14.3 (CH_3), -5.4 (CH_3). HRMS (ESI^+): m/z [M + H]$^+$ calculated for $C_{22}H_{30}N_3O_4S_2Si$ 492.1442, found 492.1444.

3-(4-(((*tert*-Butyldimethylsilyl)oxy)methyl)pyridin-2-yl)-5,6-dimethyl-2-thioxo-2,3-dihydrothieno[2,3-d]pyrimidin-4(1H)-one (17)

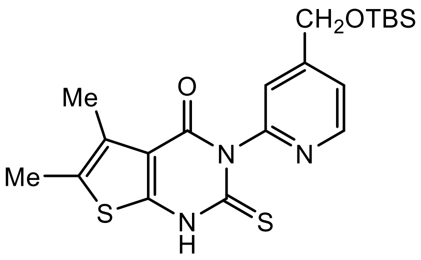

Upon heating of (pyridin-2-yl)thioureidothiophene carboxylate (**9**) (230 mg, 0.48 mmol) for 6 hours and following general procedure 106 mg (51% yield) of compound **17** were obtained as a yellowish solid. ^{1}H-NMR (400 MHz, $CDCl_3$): δ ^{1}H-NMR (400 MHz, $CDCl_3$): δ 11.65 (br s, 1H, N*H*), 8.65 (d, *J* = 5.2, 0.8 Hz, 1H, H_6-py), (dd, *J* = 5.2, 1.4 Hz, 1H), H_5-py), 7.28 (br s, 1H, H_3-py), 4.83 (s, 2H, CH_2OTBS), 2.33 (s, 3H, CH_3), 2.29 (s, 3H, CH_3), 0.95 (s, 9H, (CH_3)$_3$C-Si), 0.12 (s, 6H, (CH_3)$_2$-Si). ^{13}C{1H}-APT NMR (100 MHz, $CDCl_3$): δ 174.3 (C), 157.5 (C), 154.3 (C), 151.7 (C), 149.8 (CH), 148.2 (C), 130.6 (C), 125.5 (C), 121.2 (CH), 121.0 (CH), 118.2 (C), 63.3 (CH_2), 25.9 (CH_3), 18.4 (C), 12.7 (CH_3), 12.5 (CH_3), −5.3 (CH_3). HRMS (ESI^+): m/z [M + H]$^+$ calculated for $C_{20}H_{28}N_3O_2S_2Si$ 434.1387, found 434.1392.

3-(4-Chloropyridin-2-yl)-5,6-dimethyl-2-thioxo-2,3-dihydrothieno[2,3-d]pyrimidin-4(1H)-one (IVe)

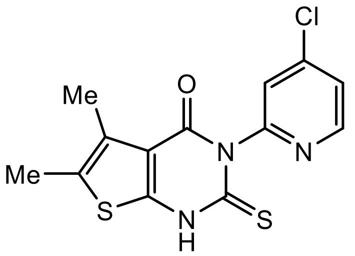

Upon heating of (pyridin-2-yl)thioureidothiophene carboxylate (**13**) (160 mg, 0.43 mmol) for 4 hours and following general procedure 85 mg (61% yield) of compound **IVe** were obtained as a pale yellow solid. M. p. = 192–193 ºC. ^{1}H-NMR (400 MHz, DMSO-d_6) δ 13.82 (s, 1H, N*H*), 8.58 (d, *J* = 5.4 Hz, 1H, H_6-py), 7.73 (d, *J* = 1.9 Hz, 1H, H_3-py), 7.63 (dd, *J* = 5.4, 2.0 Hz, 1H, H_5-py), 2.32 (s, 3H, CH_3), 2.26 (s, 3H, CH_3).^{13}C{^{1}H} NMR (100 MHz, DMSO-d_6): δ 173.4 (C), 156.9 (C), 153.2 (C), 150.5 (C), 149.2 (C), 144.1 (C), 128.9 (C), 125.8 (CH), 125.0 (CH), 124.2 (CH), 116.8 (C), 12.4 (CH_3), 12.0 (CH_3). HRMS (ESI^+): m/z [M + Na]$^+$ calculated for $C_{13}H_{10}ClN_3NaOS_2$ 345.9846, found 345.9834.

3-(4-Methoxypyridin-2-yl)-5,6-dimethyl-2-thioxo-2,3-dihydrothieno[2,3-d]pyrimidin-4(1H)-one (IVf)

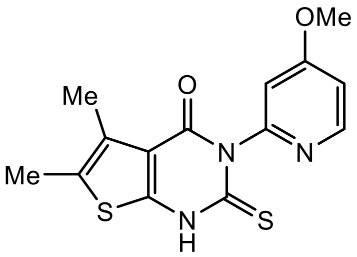

Upon heating of (pyridin-2-yl)thioureidothiophene carboxylate (**14**) (130 mg, 0.36 mmol) for 4 hours and following general procedure 85 mg (74% yield) of compound **IVf** were obtained as beige solid. M. p. = 213–214 ºC. ^{1}H-NMR (400 MHz, DMSO-*d*$_6$): δ 13.70 (s, 1H, N*H*), 8.38 (d, *J* = 5.5 Hz, 1H, H$_6$-py), 7.06 (br s, 1H, H$_3$-py), 7.05 (dd, *J* = 5.5, 2.6 Hz, H$_5$-py), 3.85 (s, 3H, OC*H*$_3$), 2.31 (s, 3H, C*H*$_3$), 2.26 (s, 3H, C*H*$_3$). ^{13}C{^{1}H} NMR (100 MHz, DMSO-*d*$_6$): δ 173.6 (C), 167.0 (C), 157.0 (C), 153.6 (C), 150.1 (CH), 128.9 (C), 125.3 (C), 116.7 (C), 110.5 (CH), 110.3 (CH), 55.7 (CH$_3$), 12.5 (CH$_3$), 12.0 (CH$_3$). HRMS (ESI$^+$): m/z [M + Na]$^+$ calculated for $C_{14}H_{13}N_3NaO_2S_2$ 342.0341, found 342.0348.

2-(5,6-Dimethyl-4-oxo-2-thioxo-1,4-dihydrothieno[2,3-d]pyrimidin-3(2H)-yl)isonicotinonitrile (IVg)

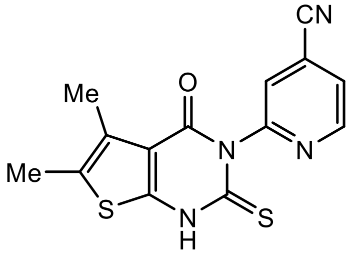

Upon heating of (pyridin-2-yl)thioureidothiophene carboxylate (**15**) (75 mg, 0.21 mmol) for 2 hours and following general procedure 50 mg (74% yield) of compound **IVg** were obtained as a beige solid. M. p. = 234–235 ºC. ^{1}H-NMR (400 MHz, DMSO-*d*$_6$) δ 13.92 (s, 1H, N*H*), 8.87 (d, *J* = 5.1 Hz, 1H, H$_6$-py), 8.09 (d, *J* = 1.1 Hz, 1H, H$_3$-py), 7.97 (dd, *J* = 5.1, 1.5 Hz, 1H, H$_5$-py), 2.33 (s, 3H, C*H*$_3$), 2.26 (s, 3H, C*H*$_3$). ^{13}C{^{1}H} NMR (100 MHz, DMSO-*d*$_6$): δ 173.4 (C), 156.9 (C), 153.0 (C), 151.0 (C), 149.3 (C), 129.0 (C), 127.2 (C), 126.0 (C), 125.8 (CH), 121.4 (CH), 116.8 (CN), 116.1 (CH), 12.4 (CH$_3$), 12.0 (CH$_3$). HRMS (ESI$^+$): m/z [M + Na]$^+$ calculated for $C_{14}H_{10}N_4NaOS_2$ 337.0188, found 337.0195.

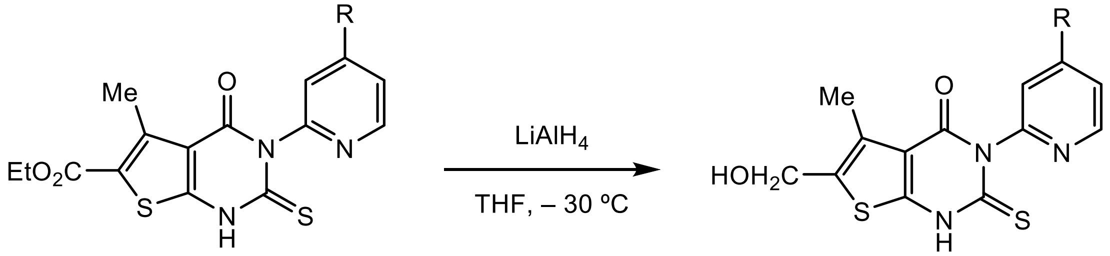

16, R = CH$_2$OTBS
IVc, R = Me

18, R = CH$_2$OTBS
IVa, R = Me

3-(4-(((*tert*-Butyldimethylsilyl)oxy)methyl)pyridin-2-yl)-6-(hydroxymethyl)-5-methyl-2-thioxo-2,3-dihydrothieno[2,3-d]pyrimidin-4(1H)-one (18)

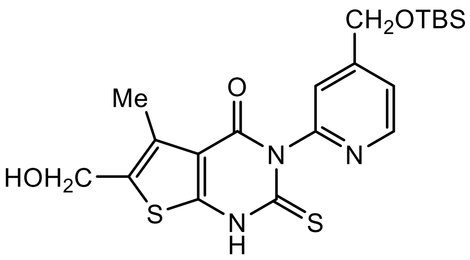

To a stirred solution of ethyl 3-(4-(((*tert*-butyldimethylsilyl)oxy)methyl)pyridin-2-yl)-5-methyl-4-oxo-2-thioxo-1,2,3,4-tetrahydrothieno[2,3-d]pyrimidine-6-carboxylate (**16**) (140 mg, 0.28 mmol) in dry THF (3 mL) at –78 ºC under argon $LiAlH_4$ 1M in THF (0.28 mL, 0.28 mmol) was added dropwise. The mixture was allowed to reach –30 ºC, stirred at that temperature for 15 h and quenched with 1M potassium carbonate aqueous solution (2.0 mL, 2.0 mmol). After evaporation of the solvent under reduced pressure acetone (10 mL) was added. The resulting suspension was filtered through a short path of silicagel and the solvent was evaporated reduced pressure to afford 74 mg (59% yield) of compound **18** as a yellow solid. M. p. = decomposes before melting. ^{1}H-NMR (400 MHz, DMSO-d_6): δ 8.39 (dd, J = 5.1, 0.8 Hz, 1H, H_6-py), 7.18 (dd, J = 5.1, 1.7 Hz, 1H, H_5-py), 7.00 (br s, 1H, H_3-py), 5.22 (t, J = 5.6 Hz, 1H, CH_2OH) 4.76 (s, 2H, CH_2OTBS), 4.40 (d, J = 5.5 Hz, 2H, CH_2OH), 2.25 (s, 3H, CH_3), 0.92 (s, 9H, $(CH_3)_3$C-Si), 0.11 (s, 6H, $(CH_3)_2$-Si). HRMS (ESI$^+$): m/z [M + H]$^+$ calculated for $C_{20}H_{28}N_3O_3S_2Si$ 450.1336, found 450.1329.

6-(Hydroxymethyl)-5-methyl-3-(4-methylpyridin-2-yl)-2-thioxo-2,3-dihydrothieno[2,3-d]pyrimidin-4(1H)-one (IVa)

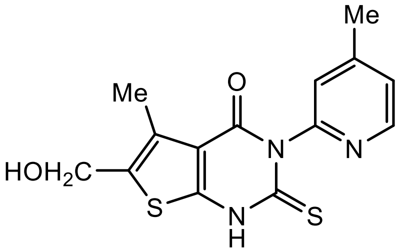

To a stirred solution of ethyl 5-methyl-3-(4-methylpyridin-2-yl)-4-oxo-2-thioxo-1,2,3,4-tetrahydrothieno[2,3-d]pyrimidine-6-carboxylate (**IVc**) (50 mg, 0.14 mmol) in dry THF (3 mL) at –78 ºC under argon $LiAlH_4$ 1 M *in* THF (0.14 mL, 0.14 mmol) was added dropwise. The mixture was allowed to reach –30 ºC, stirred at that temperature for 15 h and quenched with 1M potassium carbonate aqueous solution (1.4 mL, 1.4 mmol). After evaporation of the solvent under reduced pressure acetone (10 mL) was added. The resulting suspension was filtered through a short path of silicagel and the solvent was evaporated reduced pressure to afford 14 mg (31% yield) of compound **IVa** as an orange oil. M. p. > 300. ^{1}H-NMR (300 MHz, DMSO-d_6): δ 8.30 (d, J = 5.1 Hz, 1H, H_6-py), 7.09 (dd, J = 5.1, 1.5 Hz, 1H, H_5-py), 6.92 (br s, 1H, H_3-py), 5.46 (br s, 1H, CH_2OH), 4.50 (s, 2H, CH_2OH), 2.31 (s, 3H, CH_3), 2.25 (s, 3H, CH_3). ^{13}C{^{1}H}-APT NMR (100 MHz, DMSO-d_6): δ 174.0 (C), 160.4 (C), 155.3 (C), 147.7 (C), 128.4 (C), 127.9 (C), 125.5 (CH), 123.1 (CH), 115.5 (C), 114.1 (C), 55.9

(CH_2), 20.3 (CH_3), 13.1 (CH_3). HRMS (ESI^+): m/z $[M + Na]^+$ calculated for $C_{14}H_{13}N_3NaO_2S_2$ 342.0341, found 342.0351.

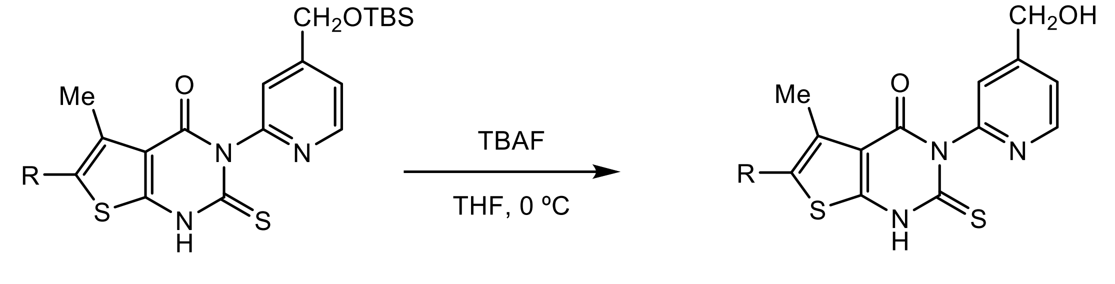

17 R = Me
18, R = CH_2OH

IVb, R = Me
IVd, R = CH_2OH

General procedure for removal of *tert*-butyldimethylsilyl protecting group

To a solution of the corresponding ((*tert*-butyldimethylsilyl)oxy)methyl derivative (0.5 mmol) in dry THF (5 mL), at 0 ºC tetrabutylammonium fluoride 1 M in THF (1.0 mL, 1.0 mmol) was added dropwise and the resulting mixture was stirred at room temperature for 1 hour. The reaction mixture was partitioned between water (2 mL) and ether (2 mL). The organic layer was separated and the aqueous layer extracted with ether (3 x 10 mL). The combined organic layers were dried over anhydrous magnesium sulphate, filtered and evaporated under reduced pressure to afford the corresponding hydroxymethyl derivative.

3-(4-(Hydroxymethyl)pyridin-2-yl)-5,6-dimethyl-2-thioxo-2,3-dihydrothieno[2,3-d]pyrimidin-4(1H)-one (IVb)

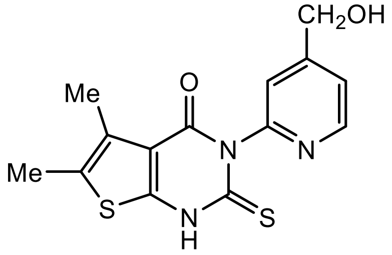

Starting from ((*tert*-butyldimethylsilyl)oxy)methyl derivative (**17**) (250 mg, 0.58 mmol) and following general procedure 146 mg (79% yield) of compound **IVb** were obtained as a white solid. M. p. = 229–230 ºC. ^{1}H-NMR (400 MHz, DMSO-d_6): δ 8.49 (d, J = 5.1 Hz, H_6-py), 7.37 (d, J = 5.1 Hz, H_5-py), 7.32 (s, 1H, H_3-py), 5.56 (t, 1H, J = 5.8 Hz, CH_2OH), 4.60 (d, J = 4.7 Hz, CH_2OH), 2.31 (s, 3H, CH_3), 2.25 (s, 3H, CH_3). $^{13}C\{^1H\}$NMR (100 MHz, DMSO-d_6): δ 173.6, 157.1, 154.5, 152.1, 149.3, 148.9, 128.9, 125.5, 121.3, 121.1, 116.8, 61.2, 12.5, 12.1. HRMS (ESI^+): m/z $[M + Na]^+$ calculated for $C_{14}H_{13}N_3NaO_2S_2$ 342.0341, found 342.0337.

6-(Hydroxymethyl)-3-(4-(hydroxymethyl)pyridin-2-yl)-5-methyl-2-thioxo-2,3-

dihydrothieno[2,3-d]pyrimidin-4(1H)-one (IVd)

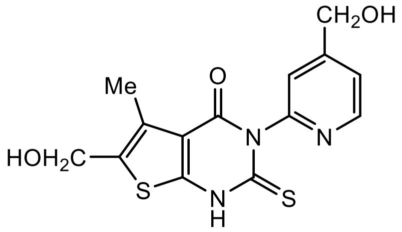

Starting from ((*tert*-butyldimethylsilyl)oxy)methyl derivative (**18**) (71 mg, 0.13 mmol) and following general procedure 44 mg (83% yield) of compound **IVd** were obtained as a yellow solid. M. p. = decomposes before melting. ^{1}H-NMR (400 MHz, DMSO-d_6): 13.8 (br s 1H, N*H*), 8.49 (d, *J* = 5.1 Hz, 1H, H_6-py), 7.38 (dd, *J* = 5.1, 1.4 Hz, 1H H_5-py), 7.33 (br s, 1H, H_3-py), 5.68 (t, *J* = 5.6 Hz, 1H, CH_2OH), 5.58 (t, *J* = 5.3 Hz, 1H, CH_2OH), 4.60 (br d, 4H, CH_2OH, CH_2OH), 2.27 (s, 3H, CH_3). ^{13}C{^{1}H}-APT NMR (100 MHz, DMSO-d_6): δ 161.2 (C), 157.6 (C), 155.6 (C), 155.4 (C), 149.5 (CH), 148.7 (C), 136.4 (C), 128.0 (C), 122.7 (CH), 121.5 (CH), 119.4 (C), 61.1 (CH_2), 56.1 (CH_2), 13,5 (CH_3). HRMS (ESI^+): m/z $[M + Na]^+$ calculated for $C_{14}H_{13}N_3NaO_3S_2$ 358.0291, found 358.0302.

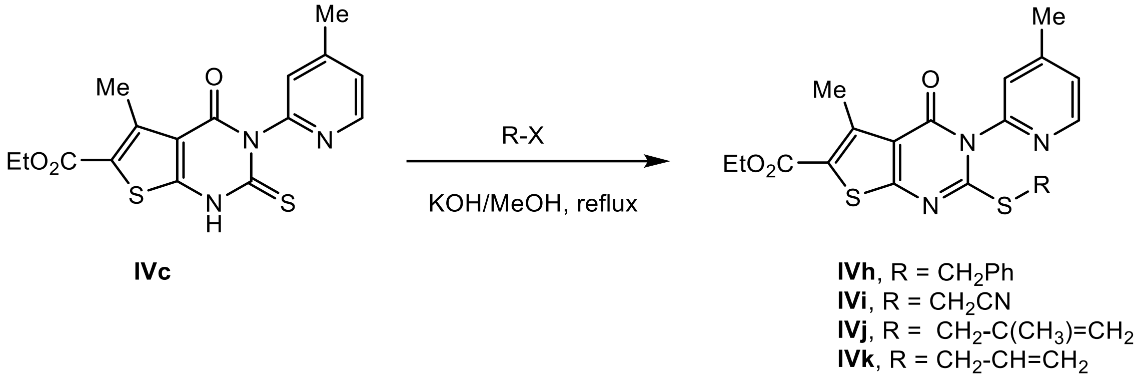

General procedure for alkylation of ethyl 5-methyl-3-(4-methylpyridin-2-yl)-4-oxo-2-thioxo-1,2,3,4-tetrahydrothieno[2,3-d]pyrimidine-6-carboxylate (IVc)

To a stirred solution of ethyl 5-methyl-3-(4-methylpyridin-2-yl)-4-oxo-2-thioxo-1,2,3,4-tetrahydrothieno[2,3-d]pyrimidine-6-carboxylate **IVc** (0.1 mmol) in 0.02 N potassium hydroxide in 70% v/v ethanol (3 mL) the corresponding alkyl halide (0.1 mmol) was added and the resulting mixture was stirred under reflux conditions for 1 hour. Then the reaction mixture was allowed to cool at room temperature and evaporated under reduced pressure to afford the corresponding S-alkyl derivative.

Ethyl 2-(benzylthio)-5-methyl-3-(4-methylpyridin-2-yl)-4-oxo-3,4-dihydrothieno[2,3-d]pyrimidine-6-carboxylate (IVh)

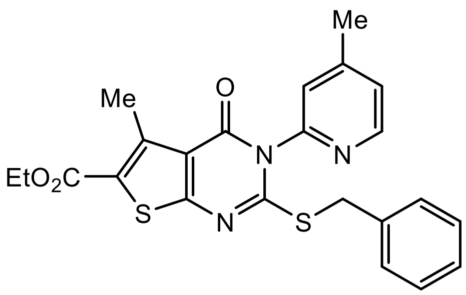

Alkylation of compound **IVc** (20 mg, 0.06 mmol) with benzyl bromide (7 µL, 10 mg, 0.06 mmol) according to general procedure yielded 25 mg (92% yield) of compound **IVh** as a white solid. M. p. = 119–120 ºC. ^{1}H-NMR (400 MHz, $CDCl_3$): δ 8.45 (d, *J* = 5.1 Hz, 1H, H_6-py), 7.29 – 7.10 (m, 7H H_5-py, H_3-py, Ph), 4.32 (s, 2H, SCH_2Ph), 4.30 (q, *J* = 7.1 Hz, 2H, $CO_2CH_2CH_3$), 2.80 (s, 3H, CH_3), 2.35, (s, 3H, CH_3), 1.33 (t, *J* = 7.1 Hz, 3H, $CO_2CH_2CH_3$).^{13}C{^{1}H}-APT NMR (100 MHz, $CDCl_3$): δ 165.4 (C), 162.7 (C), 160.0 (C), 158.7 (C), 150.7 (C), 149.9 (CH), 149.1 (C), 144.6 (C), 135.3 (C), 129.4 (CH), 128.6 (CH), 127.7 (CH), 126.1 (CH), 125.1 (CH), 121.8 (C), 120.3 (C), 61.2 (CH_2), 37.2 (CH_2), 21.0 (CH_3), 14.9 (CH_3), 14.3 (CH_3). HRMS (ESI$^+$): m/z $[M + H]^+$ calculated for $C_{23}H_{22}N_3O_3S_2$ 452.1097, found 452.1085.

Ethyl 2-((cyanomethyl)thio)-5-methyl-3-(4-methylpyridin-2-yl)-4-oxo-3,4-dihydrothieno[2,3-d]pyrimidine-6-carboxylate (IVi)

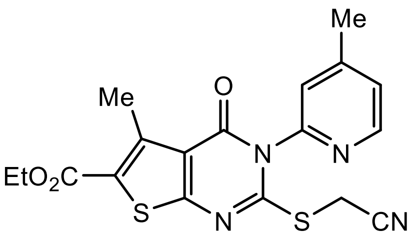

Alkylation of compound **IVc** (20 mg, 0.06 mmol) with iodoacetonitrile (4 µL, 9.2 mg, 0.06 mmol) according to general procedure yielded 22 mg (91% yield) of compound **IVi** as a white solid. M. p. = 180–181 ºC .^{1}H-NMR (400 MHz, $CDCl_3$): δ 8.55 (d, *J* = 5.1 Hz, 1H, H_6-py), 7.34 (dd, *J* = 5.1, 1.5 Hz, 1H, H_5-py), 7.26 (br s, 1H, H_3-py), 4.38 (q, *J* = 7.1 Hz, 2H, $CO_2CH_2CH_3$), 3.95 (s, 2H, SCH_2CN), 2.89 (s, 3H, CH_3), 2.50 (s, 3H, CH_3), 1.41 (t, *J* = 7.1 Hz, 3H, $CO_2CH_2CH_3$). ^{13}C{^{1}H}-APT NMR (100 MHz, $CDCl_3$): δ 164.5 (C), 162.5 (C), 158.2 (C), 156.1 (C), 151.2 (C), 149.9 (CH), 148.3 (C), 144.4 (C), 126.7 (CH), 125.1 (CH), 123.1 (C), 120.8 (C), 115.3 (C), 61.4 (CH_2), 21.1 (CH_3), 17.9 (CH_2), 14.9 (CH_3), 14.3 (CH_3). HRMS (ESI$^+$): m/z $[M + H]^+$ calculated for $C_{18}H_{17}N_4O_3S_2$ 401.0737, found 401.0729.

Ethyl 5-methyl-2-((2-methylallyl)thio)-3-(4-methylpyridin-2-yl)-4-oxo-3,4-dihydrothieno[2,3-d]pyrimidine-6-carboxylate (IVj)

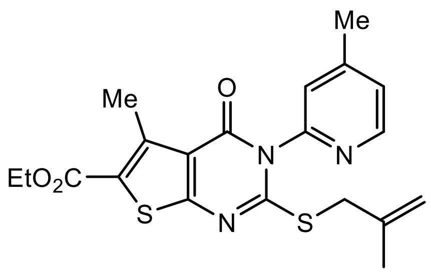

Alkylation of compound **IVc** (20 mg, 0.06 mmol) with 3-chloro-2-methyl-1-propene (6 µL, 5,5 mg, 0.06 mmol) according to general procedure yielded 21 mg (84% yield) of compound **IVj** as a yellowish solid. M. p. = 124–125 ºC. ^{1}H-NMR (400 MHz, DMSO-d_6) δ 8.54 (d, *J* = 5.1 Hz, 1H, H_6-py), 7.53 (s, 1H, H_3-py), 7.46 (d, *J* = 5.1 Hz, 1H, H_5-py), 5.02 (br s, 1H, $SCH_2C(CH_3)=C\mathit{H}H$), 4.88 (br s, 1H, $SCH_2C(CH_3)=CH\mathit{H}$), 4.32 (q, *J* = 7.1 Hz, 2H, $CO_2C\mathit{H}_2CH_3$), 3.85 (s, 2H, $SC\mathit{H}_2C(CH_3)=CH_2$), 2.77 (s, 3H, $C\mathit{H}_3$), 2.43 (s, 3H, $C\mathit{H}_3$), 1.72 (s, 3H, $SCH_2C(C\mathit{H}_3)=CH_2$), 1.32 (t, *J* = 7.1 Hz, 3H, $CO_2CH_2C\mathit{H}_3$). ^{13}C{^{1}H}-APT NMR (100 MHz, DMSO-d_6): δ 164.4 (C), 161.8 (C), 160.8 (C), 157.7 (C), 150.7 (C), 149.5 (CH), 148.8 (C), 143.7 (C), 139.3 (C), 126.4 (CH), 125.0 (CH), 120.4 (C), 119.8 (C), 115.4 (CH_2), 61.2 (CH_2), 38.5 (CH_2), 21.2 (CH_3), 20.4 (CH_3), 14.7 (CH_3), 14.1 (CH_3). HRMS (ESI$^+$): m/z $[M + H]^+$ calculated for $C_{20}H_{22}N_3O_3S_2$ 416.1097, found 416.1107.

Ethyl 2-(allylthio)-5-methyl-3-(4-methylpyridin-2-yl)-4-oxo-3,4-dihydrothieno[2,3-d]pyrimidine-6-carboxylate (IVk)

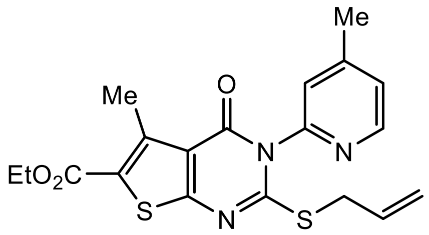

Alkylation of compound **IVc** (20 mg, 0.06 mmol) with allyl bromide (5 µL, 7 mg, 0.06 mmol) according to general procedure yielded 22 mg (91% yield) of compound **IVk** as a yellowish solid. M. p. = 127–128 ºC. ^{1}H-NMR (400 MHz, DMSO-d_6) δ 8.53 (d, *J* = 5.1 Hz, 1H, H_6-py), 7.52 (s, 1H, H_3-py), 7.46 (d, *J* = 5.1 Hz, 1H, H_5-py), 5.87 (ddt, *J* = 17.0, 10.0, 6.9 Hz, 1H, $SCH_2C\mathit{H}=CH_2$), 5.31 (dd, *J* = 17.0, 1.7 Hz, 1H, $SCH_2CH=C\mathit{H}H$), 5.15 (dd, *J* = 10.0, 1.7 Hz, 1H, $SCH_2CH=CH\mathit{H}$), 4.32 (q, *J* = 7.1 Hz, 2H, $CO_2C\mathit{H}_2CH_3$), 3.82 (d, *J* = 6.9 Hz, 2H, $SC\mathit{H}_2CH= CH_2$), 2.78 (s, 3H, $C\mathit{H}_3$), 2.42 (s, 3H, $C\mathit{H}_3$), 1.32 (t, *J* = 7.1 Hz, 3H, $CO_2CH_2C\mathit{H}_3$). ^{13}C{^{1}H}-APT NMR (100 MHz, DMSO-d_6): δ 164.5 (C), 161.8 (C), 160.6 (C), 157.7 (C), 150.7 (C), 149.5 (CH), 148.8 (C), 143.7 (C), 132.2 (CH), 126.4 (CH), 125.0 (CH), 120.5 (C), 119.8 (C), 119.4 (CH_2), 61.2 (CH_2), 34.5 (CH_2), 20.4 (CH_3), 14.7 (CH_3), 14.1 (CH_3). HRMS (ESI$^+$): m/z $[M + H]^+$ calculated for $C_{19}H_{20}N_3O_3S_2$ 402.0941, found 402.0947.

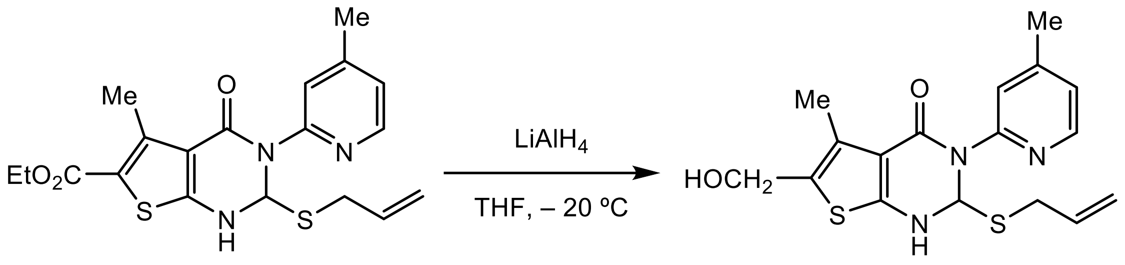

IVk **IVl**

2-(Allylthio)-6-(hydroxymethyl)-5-methyl-3-(4-methylpyridin-2-yl)thieno[2,3-d]pyrimidin-4(3H)-one (IVl)

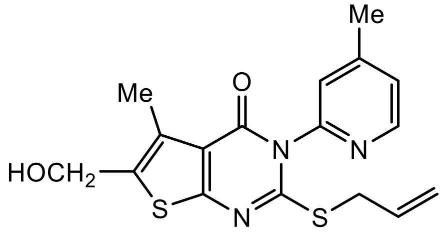

To a stirred solution ethyl 2-(allylthio)-5-methyl-3-(4-methylpyridin-2-yl)-4-oxo-3,4-dihydrothieno[2,3-d]pyrimidine-6-carboxylate (**IVk**) (30 mg, 0.07 mmol) in dry THF (3 mL) at −78 ºC under argon $LiAlH_4$ 1M in THF (0.07 mL, 0.07 mmol) was added dropwise. The mixture was allowed to reach −20 ºC, stirred at that temperature for 4 h and quenched with 1M potassium carbonate aqueous solution (2.0 mL, 2.0 mmol). The obtained solution was extracted with ethyl acetate (3 x 10 mL). The combined organic layers were dried over anhydrous magnesium sulphate, filtered and evaporated under reduced pressure to afford the corresponding to afford 24 mg (95% yield) of compound **IVl** as a yellow solid. ^{1}H-NMR (400 MHz $CDCl_3$) δ 8.55 (d, J = 5.1 Hz, 1H, H_6-py), 7.29 (dd, J = 5.1, 1.6 Hz, 1H, H_5-py, 7.21 (br s, 1H, H_3-py), 5.90 (ddt, J = 17.0, 10.0, 7.1 Hz, 1H, $SCH_2CH{=}CH_2$), 5.28 (ddd, J =17.0, 1.4, 1.1 Hz, 1H, $SCH_2CH{=}CHH$), 5.14 (dd, J =10.0, 1.4, 1.1 Hz, 1H, $SCH_2CH{=}CHH$), 4.80 (s, 2H, CH_2OH), 3.82 (ddd, J = 7.1, 1.1, 1.1 Hz, 2H, $SCH_2CH{=}CH_2$), 2.46 (s, 3H, CH_3), 2.44 (s, 3H, CH_3). HRMS (ESI^+): m/z $[M + H]^+$ calculated for $C_{17}H_{18}N_3O_3S_2$ 360.0835, found 360.0827.

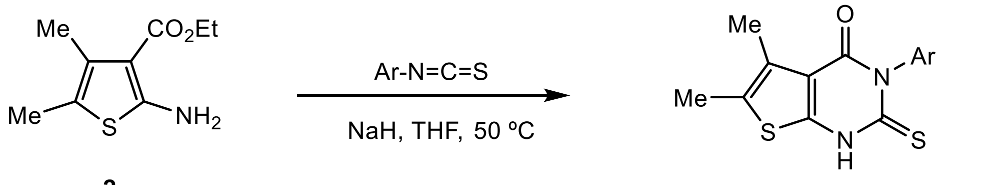

2

IVm, Ar = 3,5-$(CH_3)_2C_6H_3$
IVn, Ar = 1-naphthyl
IVo, Ar = 5,6,7,8-tetrahydronaphthalen-1-yl)

General procedure for the synthesis of 3-aryl-5,6-dimethyl-2-thioxo-2,3-dihydrothieno[2,3-d]pyrimidin-4(1H)-ones

A predried Schlenk tube was charged with ethyl 2-amino-4,5-dimethylthiophene-3-carboxylate (**2**) (1 mmol), sodium hydride 60% dispersion in mineral oil (1 mmol) and the corresponding isothiocyanate (1 mmol). THF (8 mL) was added under argon and the solution was stirred for 2 hours at 50 ºC until the reaction was complete (TLC). After this solvent was evaporated under reduced pressure and the resulting residue was purified by column chromatography to afford the corresponding 3-aryl-5,6-dimethyl-2-thioxo-2,3-dihydrothieno[2,3-d]pyrimidin-4(1H)-one.

3-(3,5-Dimethylphenyl)-5,6-dimethyl-2-thioxo-2,3-dihydrothieno[2,3-d]pyrimidin-4(1H)-one (IVm)

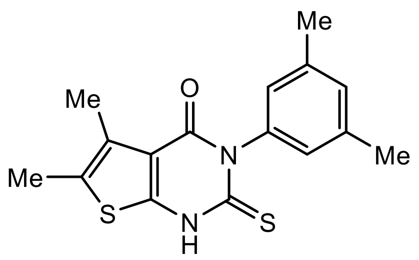

Starting from thiophene **12 (**200 mg, 1.00 mmol) and 1-isothiocyanato-3,5-dimethylbenzene (163 mg, 1.00 mmol) and following general procedure 250 mg (79% yield) of compound **IVm** were obtained as a white solid. (Eluent column chromatography, Et_2O/hexane 2:3). M. p. = decomposes before melting. ^{1}H-NMR (400 MHz, DMSO-d_6): δ 13.60 (s, 1H, N*H*), 7.02 (s, 1H, H_4-Ph), 6.81 (s, 2H, $H_{2,6}$-Ph), 2.30 (s, 3H, tf-CH_3), 2.29 (s, 6H, Ph-CH_3), 2.24 (s, 3H, tf-CH_3). ^{13}C{^{1}H}-APT NMR (100 MHz, DMSO-d_6): δ 174.3 (C), 157.1 (C), 148.6 (C), 139.1 (C), 138.0 (C), 129.5 (CH), 129.0 (C), 126.3 (CH), 125.2 (C), 116.8 (C), 20.7 (CH_3), 12.5 (CH_3), 12.0 (CH_3). HRMS (ESI^+): m/z $[M + Na]^+$ calculated for $C_{16}H_{16}N_2NaOS_2$ 339.0596, found 339.0581.

5,6-Dimethyl-3-(naphthalen-1-yl)-2-thioxo-2,3-dihydrothieno[2,3-d]pyrimidin-4(1H)-one (IVn)

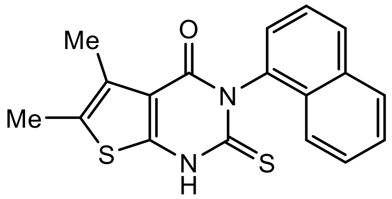

Starting from thiophene **2 (**200 mg, 1.00 mmol) and 1-isothiocyanatonaphthalene (189 mg, 1.00 mmol) and following general procedure 270 mg (80% yield) of compound **IVn** were obtained as a yellow solid (Eluent column chromatography, Et_2O/hexane 1:4). M. p. : decomposes before melting. ^{1}H-NMR (400 MHz, DMSO-d_6): δ 13.79 (br s, 1H, NH), 8.00 (t, *J* = 8.0 Hz, 2H, Naphthyl), 7.79–7.21 (m, 5H, Naphthyl),2,33 (s, 3H, CH_3), 2,24 (s, 3H, CH_3). ^{13}C{^{1}H}-APT NMR (100 MHz, DMSO-d_6): δ 174.4 (C), 157.3 (C), 149.3

(C), 136.0 (C), 133.9 (C), 129.6 (C), 129.0 (C), 128.4 (CH), 128.3 (CH), 127.1 (CH), 126.8 (CH), 126.1 (CH), 125.8 (CH), 125.4 (C), 122.3 (CH), 116.8 (C), 12.5 (CH_3), 12.1 (CH_3). HRMS (ESI^+): m/z $[M + Na]^+$ calculated for $C_{18}H_{14}N_2NaOS_2$ 361.0440, found 361.0431.

5,6-Dimethyl-3-(5,6,7,8-tetrahydronaphthalen-1-yl)-2-thioxo-2,3-dihydrothieno[2,3-d]pyrimidin-4(1H)-one (IVo)

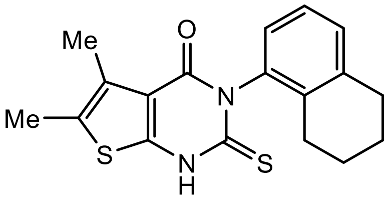

Starting from thiophene **2 (**125 mg, 0.63 mmol) and 1-5-isothiocyanato-1,2,3,4-tetrahydronaphthalene[1] (120 mg, 0.63 mmol) and following general procedure 160 mg (74% yield) of compound **IVo** were obtained as a beige solid (Eluent column chromatography, Et_2O/hexane 2:3). M. p. : decomposes before melting. ^{1}H-NMR (400 MHz, DMSO-d_6): δ ^{1}H-NMR (400 MHz, DMSO-d_6): δ 13.66 (s, 1H, NH), 7.17 (dd, J = 7.6, 7.6 Hz, 1H, H_3-Naphthyl), 7.11 (d, J = 7.6 Hz, 1H, H_4-Naphthyl), 6.93 (d, J = 7.6 Hz, 1H, H_2-Naphthyl), 2.81 – 2.73 (m, 2H, H_8-Naphthyl), 2.46–2.40 (m, 1H, HH$_5$-Naphthyl), 2.30 (s, 3H, CH_3), 2.25 (s, 3H, CH_3), 2.26 – 2.20 (m, 1H, HH_5-Naphthyl), 1.74 – 1.60 (m, 4H, H_7,H_6-Naphthyl). ^{13}C{^{1}H}-APT NMR (100 MHz, DMSO-d_6): δ 173.6 (C), 156.7 (C), 148.9 (C), 138.0 (C), 137.9 (C), 133.8 (C), 129.0 (CH), 126.0 (CH), 126.0 (CH), 125.4 (C), 116.6 (C), 28.9 (CH_2), 23.7 (CH_2), 22.3 (CH_2), 22.0 (CH_2), 12.5 (CH_3), 12.0 (CH_3). HRMS (ESI^+): m/z $[M + Na]^+$ calculated for $C_{18}H_{18}N_2NaOS_2$ 365.0753, found 365.0755.

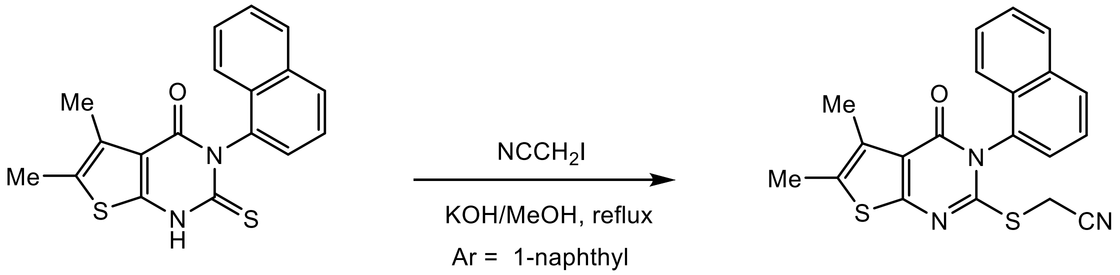

IVn **IVp**

2-((5,6-Dimethyl-3-(naphthalen-1-yl)-4-oxo-1,2,3,4-tetrahydrothieno[2,3-d]pyrimidin-2-yl)thio)acetonitrile (IVp)

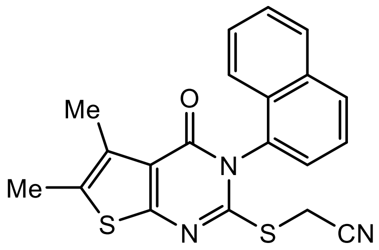

To a stirred solution of 5,6-dimethyl-3-(naphthalen-1-yl)-2-thioxo-2,3-dihydrothieno[2,3-d]pyrimidin-4(1H)-one (**IVn**) (45 mg, 0.13 mmol) in 0.02 M potassium hydroxide 70% in ethanol (3 mL) iodoacetonitrile (10 μL, 23 mg, 0.14 mmol) was added and the resulting mixture was stirred under reflux conditions for 6 hours. Then the reaction mixture was allowed to cool at room temperature and evaporated under reduced pressure to afford 33 mg (67% yield) of compound **IVp** as a white solid. M. p. = 235–236 ºC. ^{1}H-NMR (400 MHz, DMSO-d_6): δ 8.20 (d, J = 8.2 Hz, 1H, H_8-Naphthyl), 8.11 (d, J = 8.0 Hz, 1H, H_5-Naphthyl), 7.77 (d, J = 7.3 Hz, 1H, H_4-Naphthyl), 7.71 (dd, J = 8.3, 7.3 Hz, 1H, H_3-Naphthyl), 7.63 (dd, J = 8.2, 7.5 Hz, 1H, H_7-Naphthyl), 7.58 (dd, J = 8.0, 7.5 Hz, 1H, H_6-Naphthyl), 7.46 (d, J = 8.3 Hz, 1H, H_2-Naphthyl), 4.10 (s, 2H, SCH_2CN), 2.42 (s, 3H), 2.36 (s, 3H). ^{13}C{^{1}H}-APT NMR (100 MHz, DMSO-d_6): δ 160.5 (C), 157.5 (C), 155.0 (C), 133.9 (C), 131.5 (C), 130.9 (CH), 129.2 (C), 129.2 (C), 129.0 (C), 128.6 (CH), 128.5 (CH), 128.1 (CH), 126.9 (CH), 125.8 (CH), 121.4 (CH), 119.8 (C), 117.2 (C), 17.8 (CH_2), 12.7 (CH_3), 12.6 (CH_3). HRMS (ESI$^+$): m/z [M + H]$^+$ calculated for $C_{20}H_{18}N_3OS_2$ 380.0886, found 380.0893.

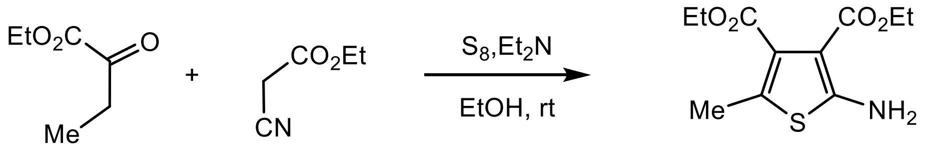

19

Diethyl 2-amino-5-methylthiophene-3,4-dicarboxylate (19)

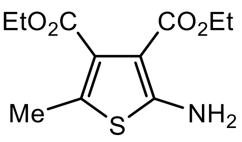

To a stirred mixture of ethyl 2-oxobutanoate (651 mg, 5.0 mmol), ethyl 2-cyanoacetate (565 mg, 5.0 mmol) and powdered sulfur (181 mg, 0,7 mmol) in ethanol (20 mL) a solution of diethylamine (361 mg, 5.0 mmol) in ethanol (10 mL) was added dropwise and the resulting mixture was stirred for 20 h at room temperature. After this solvent was evaporated under reduced pressure and the resulting residue was purified by column chromatography (Eluent, hexane/ Et_2O 7:3) to afford 400 mg (31% yield) of compound **19** as a white solid. M. p. = 116–117 ºC. IR (KBr) 1672, 1703, 3344, 3456. ^{1}H-NMR (400 MHz, $CDCl_3$): δ 5.70 (br s, 2H, NHH), 4.28 (q, J = 7.2 Hz, 2H, OCH_2CH$_3$), 4.21 (q, J = 7.2 Hz, 2H, OCH_2CH$_3$), 2.24 (s, 3H, CH_3), 1.33 (t, J = 7.2 Hz, 3H, OCH$_2$CH_3), 1.27 (t, J = 7.2 Hz, 3H, OCH$_2$CH_3).

$^{13}C\{^1H\}$-APT NMR (100 MHz, $CDCl_3$): δ 165.9 (C), 164.3 (C), 160.7 (C), 128.5 (C), 122.3 (C), 104.6 (C), 61.1 (CH_2), 59.9 (CH_2), 14.2 (CH_3), 14.1 (CH_3), 13.0 (CH_3). HRMS (ESI^+): m/z $[M + Na]^+$ calculated for $C_{11}H_{15}NNaO_4S$ 280.0615, found 280.0617.

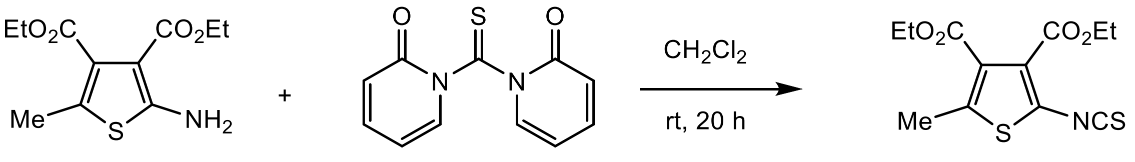

19 **20**

Diethyl 2-isothiocyanato-5-methylthiophene-3,4-dicarboxylate (20)

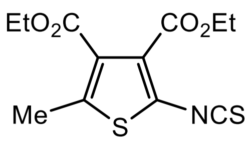

To a solution of diethyl 2-amino-5-methylthiophene-3,4-dicarboxylate (**19**) (386 mg, 1.5 mmol) in dry dichloromethane (25 mL), 1,1'-thiocarbonylbis(pyridin-2(1*H*)-one) (348 mg, 1.5 mmol) was added and the resulting solution was stirred at room temperature for 20 hours. Then solvent was removed under reduced pressure and the resulting residue was purified by column chromatography (Eluent, hexane/ Et_2O 9:1) to afford 302 mg (67% yield) of compound **20** as a yellowish oil. IR (NaCl) 1721, 2093. ^1H-NMR (400 MHz, $CDCl_3$): δ 4.34 (q, J = 7.2 Hz, 2H, OCH_2CH_3), 4.30 (q, J = 7.2 Hz, 2H, OCH_2CH_3), 2.50 (s, 3H, CH_3), 1.37 (t, J = 7.2 Hz, 3H, OCH_2CH_3), 1.32 (t, J = 7.2 Hz, 3H, OCH_2CH_3). $^{13}C\{^1H\}$-APT NMR (100 MHz, $CDCl_3$): δ 162.9 (C), 161.5 (C), 139.9 (C), 139.3 (C), 130.5 (C), 128.3 (C), 128.2 (C), 61.6 (CH_2), 61.3 (CH_2), 14.2 (CH_3), 14.1 (CH_3), 14.0 (CH_3). HRMS (ESI^+): m/z $[M + Na]^+$ calculated for $C_{12}H_{13}NNaO_4S_2$ 322.0179, found 322.0169.

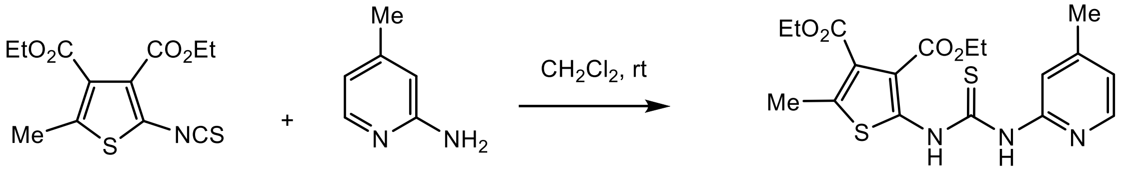

20 **5** **21**

Diethyl 2-methyl-5-(3-(4-methylpyridin-2-yl)thioureido)thiophene-3,4-dicarboxylate (21)

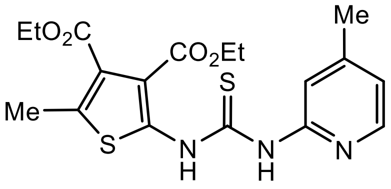

To a solution of diethyl 2-isothiocyanato-5-methylthiophene-3,4-dicarboxylate (**20**) (239 mg, 0.80 mmol) in dry dichloromethane (20 mL), 4-methylpyridin-2-amine (173 mg, 1.60 mmol) was added and the resulting solution was stirred at room temperature for 20 h. Then solvent

was removed under reduced pressure and the resulting residue was purified by column chromatography (Eluent, hexane/ Et_2O 3:2) to afford 311 mg (95% yield) of compound **21** as a yellow solid. M. p. 161–162 ºC. IR (KBr) 1189, 1709, 3237, 3299. ^{1}H-NMR (400 MHz, $CDCl_3$): δ 9.10 (br s, 1H, N*H*), 8.23 (d, *J* = 5.2 Hz, 1H, H_6-py), 6.80 (d, *J* = 5.2 Hz, 1H, H_5-py), 6.68 (br s, 1H, H_3-py), 4.31 (q, *J* = 7.1 Hz, 2H, CO_2-C*H*$_2$CH$_3$), 4.31 (q, = 7.1 Hz, 2H, CO_2-C*H*$_2$CH$_3$), 2.41 (s, 3H, CH_3), 2.27 (s, 3H, CH_3), 1.33 (t, *J* = 7.1 Hz, 3H, CO_2-CH$_2$C*H*$_3$), 1.29 (t, *J* = 7.1 Hz, 3H, CO_2-CH$_2$C*H*$_3$). ^{13}C{^{1}H}-APT NMR (100 MHz, $CDCl_3$): δ 175.4 (C), 165.5 (C), 163.6 (C), 151.9 (C), 150.6 (C), 145.9 (C), 145.1 (CH), 132.4 (C), 126.9 (C), 119.9 (CH), 115.8 (C), 111.6 (CH), 61.0 (CH_2), 60.7 (CH_2), 21.1 (CH_3), 14.1 (CH_3), 14.0 (CH_3), 13.1 (CH_3). HRMS (ESI$^+$): m/z [M + Na]$^+$ calculated for $C_{18}H_{21}N_3NaO_4S_2$ 430.0866, found 430.0870.

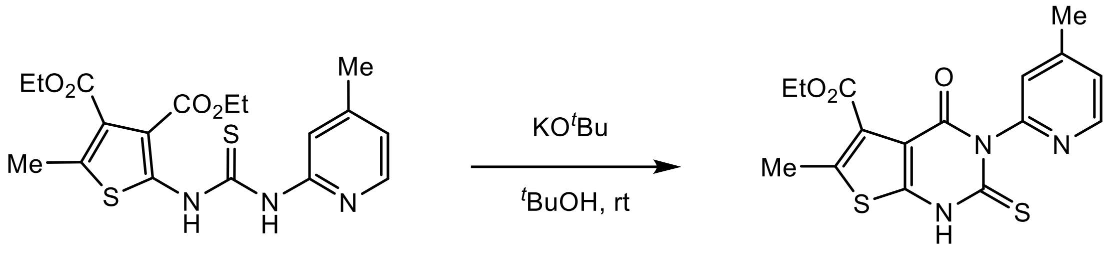

21 **IVq**

Ethyl 6-methyl-3-(4-methylpyridin-2-yl)-4-oxo-2-thioxo-1,2,3,4-tetrahydrothieno[2,3-d]pyrimidine-5-carboxylate (IVq)

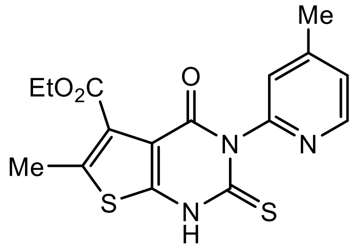

To a suspension of diethyl 2-methyl-5-(3-(4-methylpyridin-2-yl)thioureido)thiophene-3,4-dicarboxylate (**21**) (407 mg, 1.0 mmol) in *tert*-butanol (25 mL), potassium *tert*-butoxide (135 mg, 1.2 mmol) was added and the resulting mixture was stirred at room temperature until the disappearance of the yellow colour (1.5 hours). The reaction mixture was diluted with ethanol until complete dissolution of the solid and then concentrated under reduced pressure and adsorbed on siligacel. Subsequent purification by column chromatography (1st eluent, 2nd Et_2O, eluent, AcOEt/MeOH 9:1) afforded 306 mg (85% yield) of compound **22** as a white solid. M. p. 198-199 ºC (decomposes). IR (KBr) 1219, 1703, 1722. ^{1}H-NMR (400 MHz, DMSO-*d*$_6$): δ 13.82 (br s, 1H, N*H*) 8.42 (d, *J* = 5.2 Hz, 1H, H_6-py), 7.29 (d, *J* = 5.2 Hz, 1H, H_5-py), 7.27 (s, 1H, H_3-py), 4.22 (q, *J* = 7.2 Hz, 3H, CO_2-C*H*$_2$CH$_3$), 2.42 (s, 3H, C*H*$_3$), 2.37 (s, 3H, C*H*$_3$), 1.22 (t, *J* = 7.2 Hz, 3H, CO_2-CH$_2$C*H*$_3$). ^{13}C{^{1}H}-APT NMR (100 MHz, DMSO-*d*$_6$): δ 174.2 (C), 163.3 (C), 155.1 (C),

151.8 (C), 149.7 (C), 149.6 (C), 148.9 (CH), 133.9 (C), 126.1 (C), 125.0 (CH), 124.8 (CH), 115.3 (C), 61.2 (CH_2), 20.3 (CH_3), 14.0 (CH_3), 13.1 (CH_3). HRMS (ESI^+): m/z $[M + Na]^+$ calculated for $C_{16}H_{15}N_3NaO_3S_2$ 384.0448, found 384.0462.

[1] 1-5-Isothiocyanato-1,2,3,4-tetrahydronaphthalene was prepared as follows. To a stirred solution of 5,6,7,8-tetrahydronaphthalen-1-amine (147 mg, 1.0 mmol) in dichloromethane 1,1’-thiocarbonyldiimidazole (206 mg, 1.0 mmol) was added and the resulting mixture was stirred at room temperature for 2 hours. Then the reaction mixture was evaporated under reduced pressure to afford 140 mg (74% yield) of isothiocyanate as a yellow oil.

References

1. Torreblanca, R.; Lira-Navarrete, E.; Sancho, J.; Hurtado-Guerrero, R. Structural and mechanistic basis of the interaction between a pharmacological chaperone and human phenylalanine hydroxylase. *Chembiochem : a European journal of chemical biology* **2012**, *13*, 1266-1269, doi:10.1002/cbic.201200188.
2. Daina, A.; Zoete, V. A BOILED-Egg To Predict Gastrointestinal Absorption and Brain Penetration of Small Molecules. *ChemMedChem* **2016**, *11*, 1117-1121, doi:https://doi.org/10.1002/cmdc.201600182.
3. Daina, A.; Michielin, O.; Zoete, V. SwissADME: a free web tool to evaluate pharmacokinetics, drug-likeness and medicinal chemistry friendliness of small molecules. *Scientific Reports* **2017**, *7*, 42717, doi:10.1038/srep42717.
4. Delaney, J.S. ESOL: estimating aqueous solubility directly from molecular structure. *Journal of chemical information and computer sciences* **2004**, *44*, 1000-1005, doi:10.1021/ci034243x.
5. Ertl, P.; Rohde, B.; Selzer, P. Fast calculation of molecular polar surface area as a sum of fragment-based contributions and its application to the prediction of drug transport properties. *J Med Chem* **2000**, *43*, 3714-3717, doi:10.1021/jm000942e.
6. Lipinski, C.A.; Lombardo, F.; Dominy, B.W.; Feeney, P.J. Experimental and computational approaches to estimate solubility and permeability in drug discovery and development settings. *Advanced drug delivery reviews* **2001**, *46*, 3-26, doi:10.1016/s0169-409x(00)00129-0.
7. Veber, D.F.; Johnson, S.R.; Cheng, H.-Y.; Smith, B.R.; Ward, K.W.; Kopple, K.D. Molecular Properties That Influence the Oral Bioavailability of Drug Candidates. *Journal of Medicinal Chemistry* **2002**, *45*, 2615-2623, doi:10.1021/jm020017n.
8. Egan, W.J.; Merz, K.M., Jr.; Baldwin, J.J. Prediction of drug absorption using multivariate statistics. *J Med Chem* **2000**, *43*, 3867-3877, doi:10.1021/jm000292e.

NMR spectra

Diethyl 5-isothiocyanato-3-methylthiophene-2,4-dicarboxylate (3)

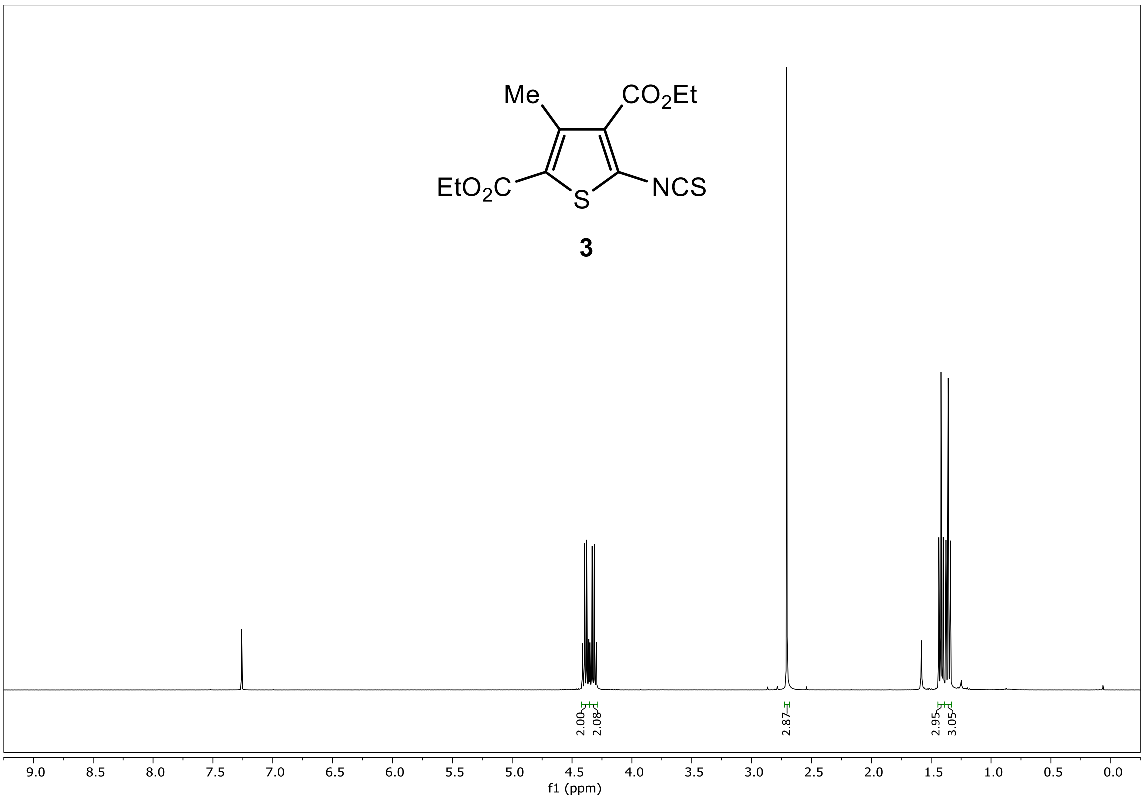

^{1}H-NMR (400 MHz, $CDCl_3$)

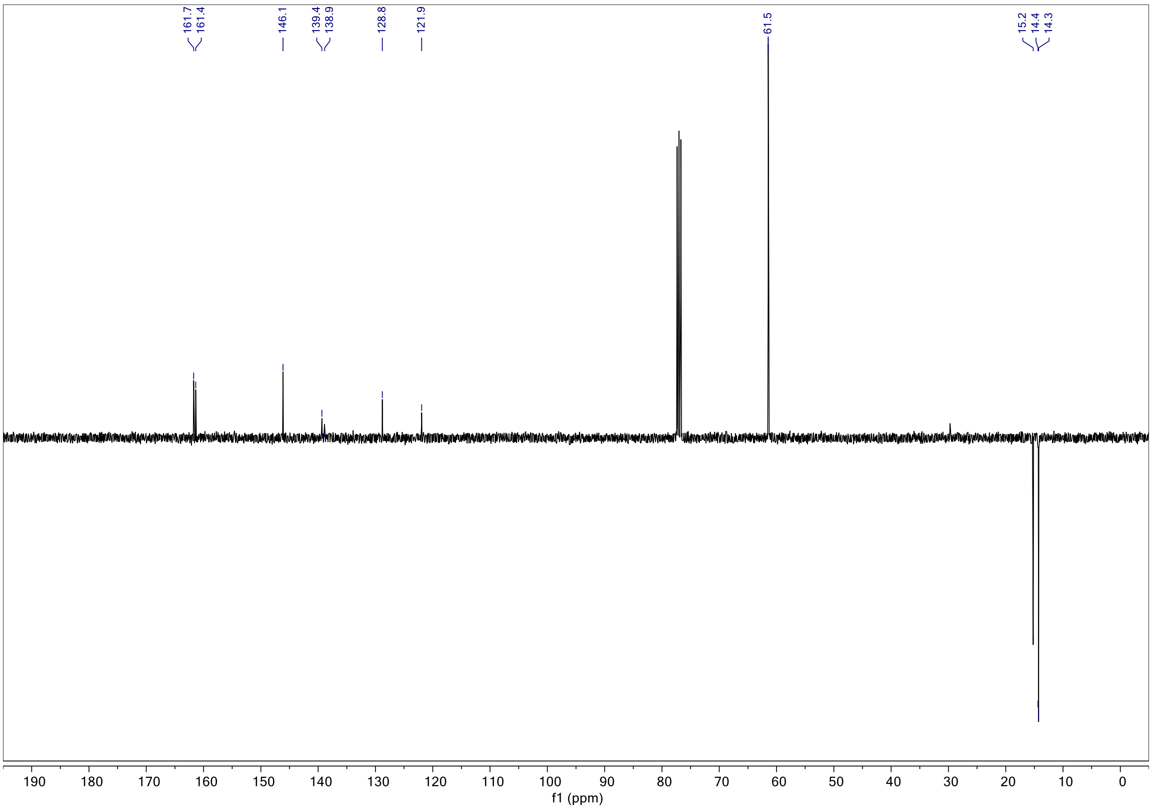

^{13}C{^{1}H}-APT NMR (100 MHz, $CDCl_3$)

Ethyl 2-isothiocyanato-4,5-dimethylthiophene-3-carboxylate (4)

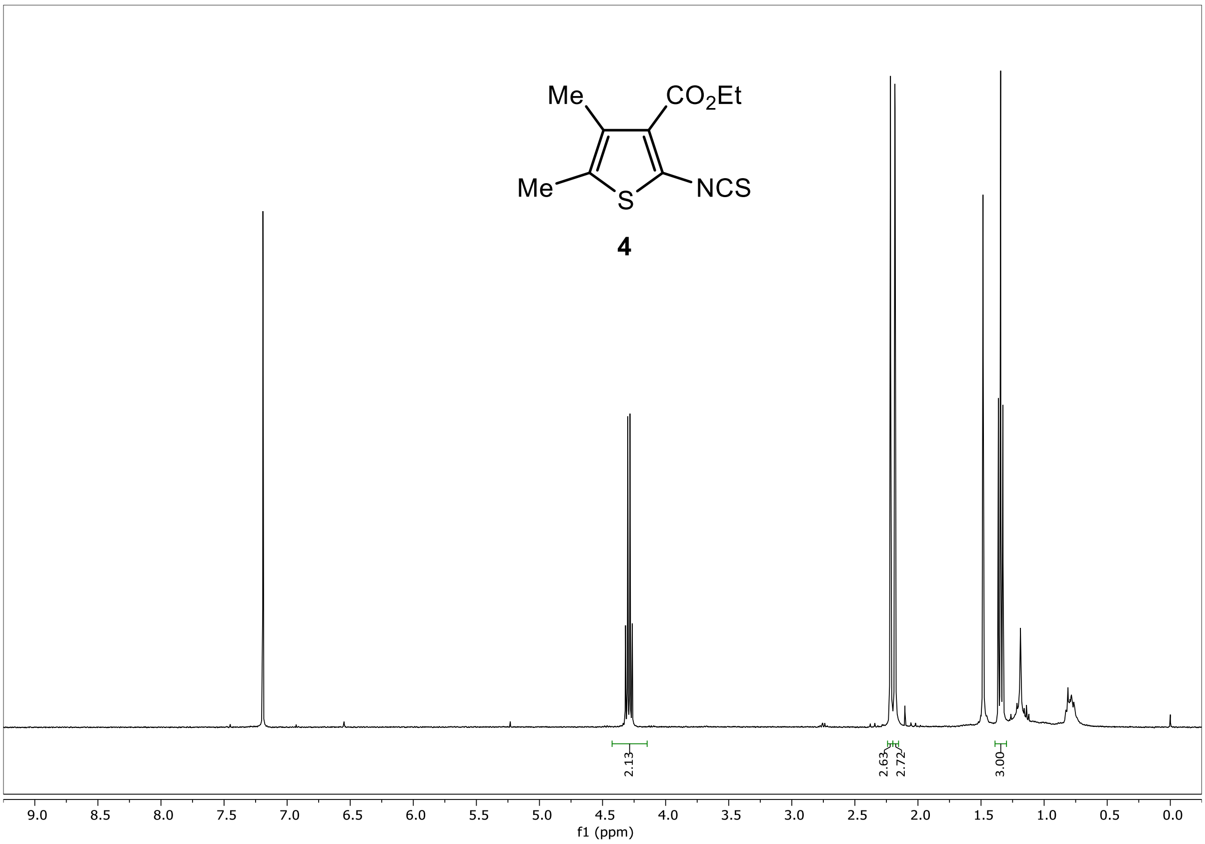

^{1}H-NMR (400 MHz, $CDCl_3$)

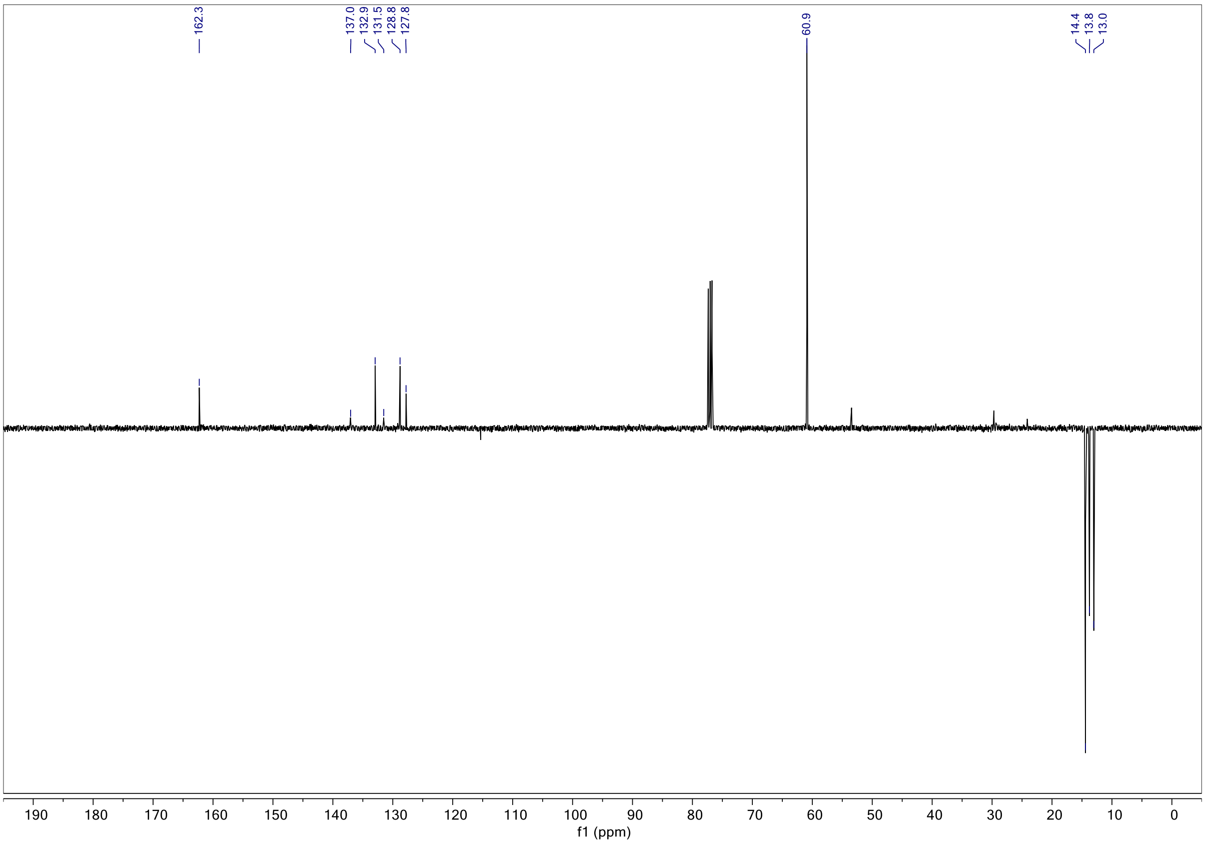

^{13}C{^{1}H}-APT NMR (100 MHz, $CDCl_3$)

Diethyl 3-methyl-5-(3-(4-methylpyridin-2-yl)thioureido)thiophene-2,4-dicarboxylate (7)

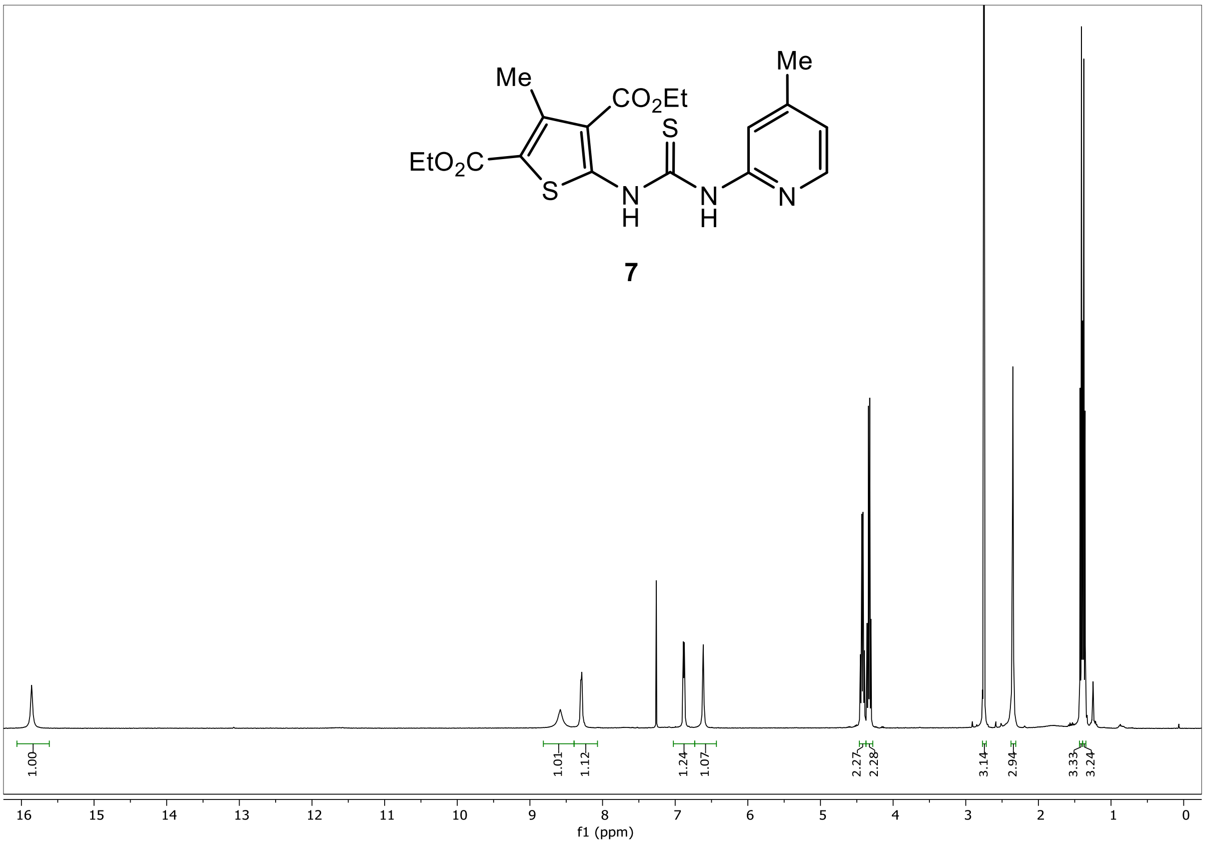

^{1}H-NMR (400 MHz, $CDCl_3$)

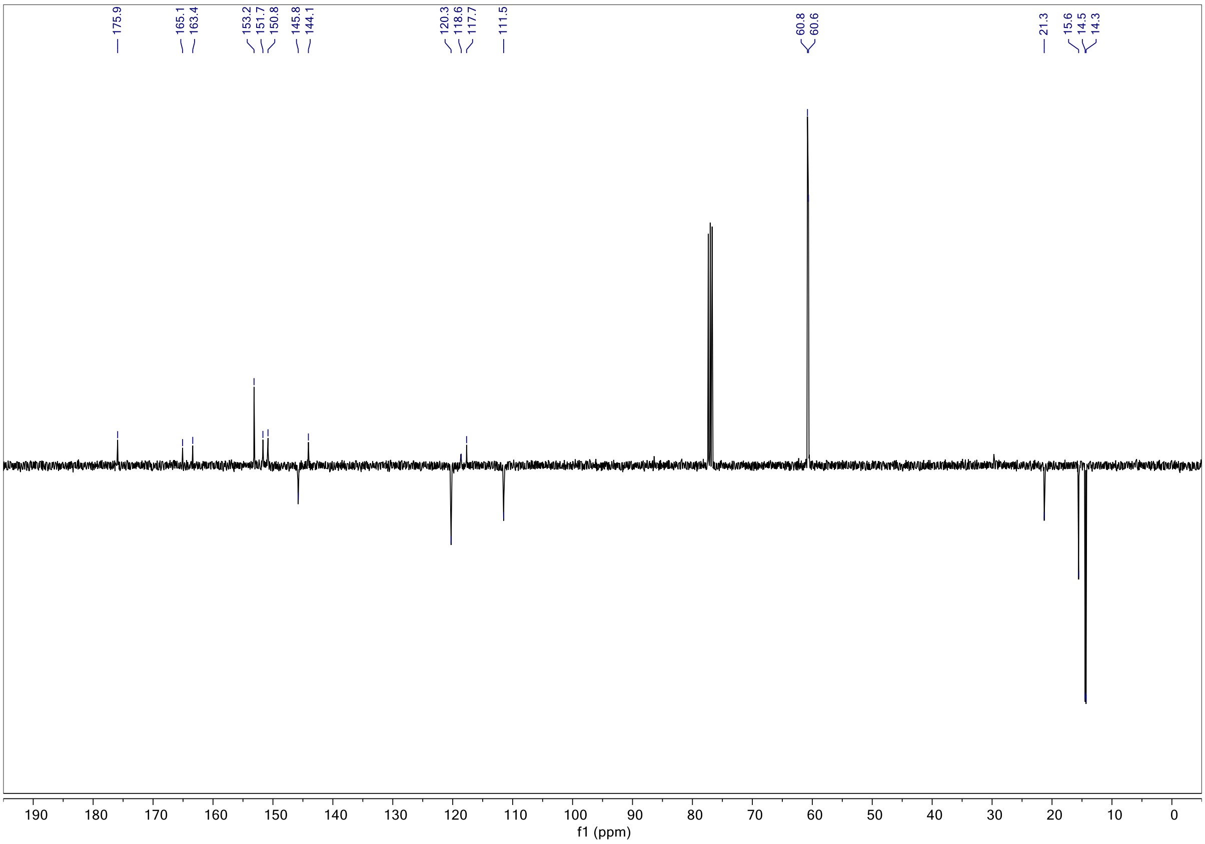

^{13}C{^{1}H}-APT NMR (100 MHz, $CDCl_3$)

Diethyl 5-(3-(4-(((*tert*-butyldimethylsilyl)oxy)methyl)pyridin-2-yl)thioureido)-3-methylthiophene-2,4-dicarboxylate (8)

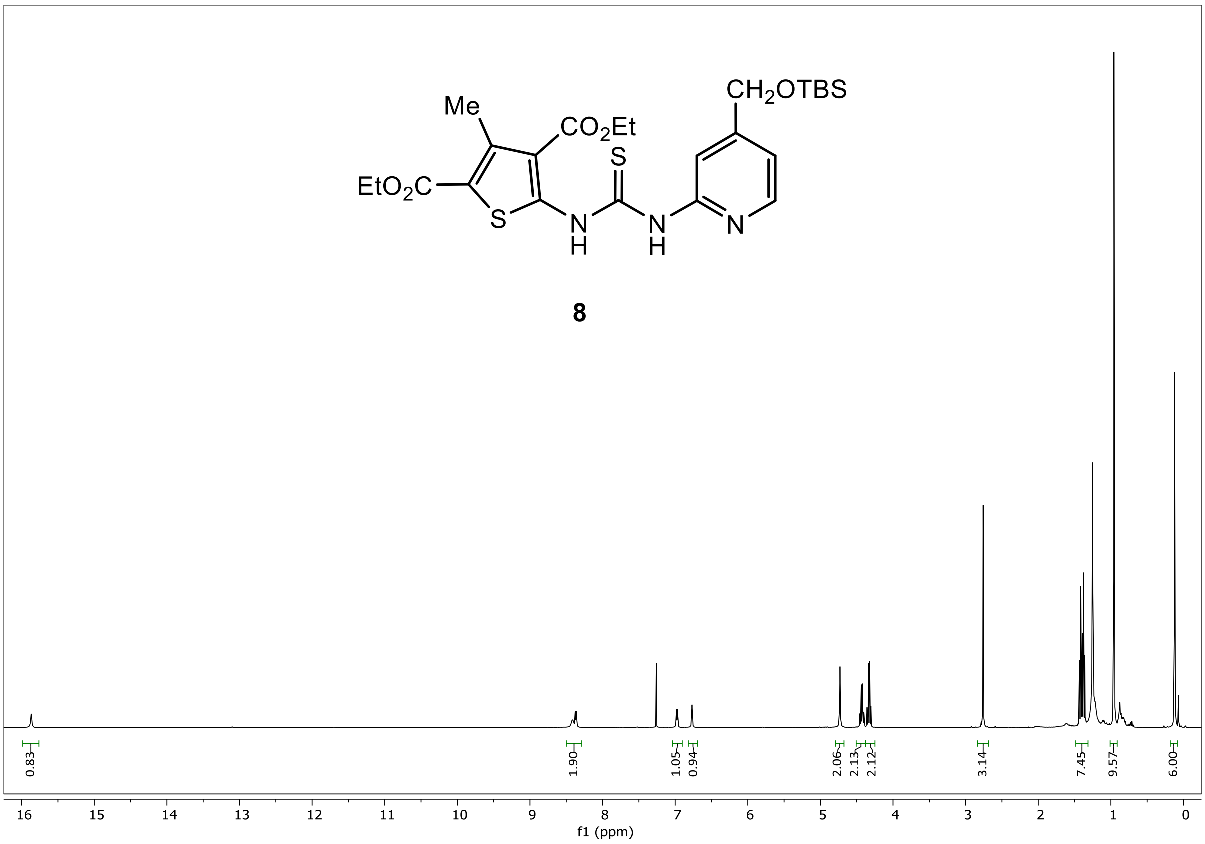

^{1}H-NMR (400 MHz, $CDCl_3$)

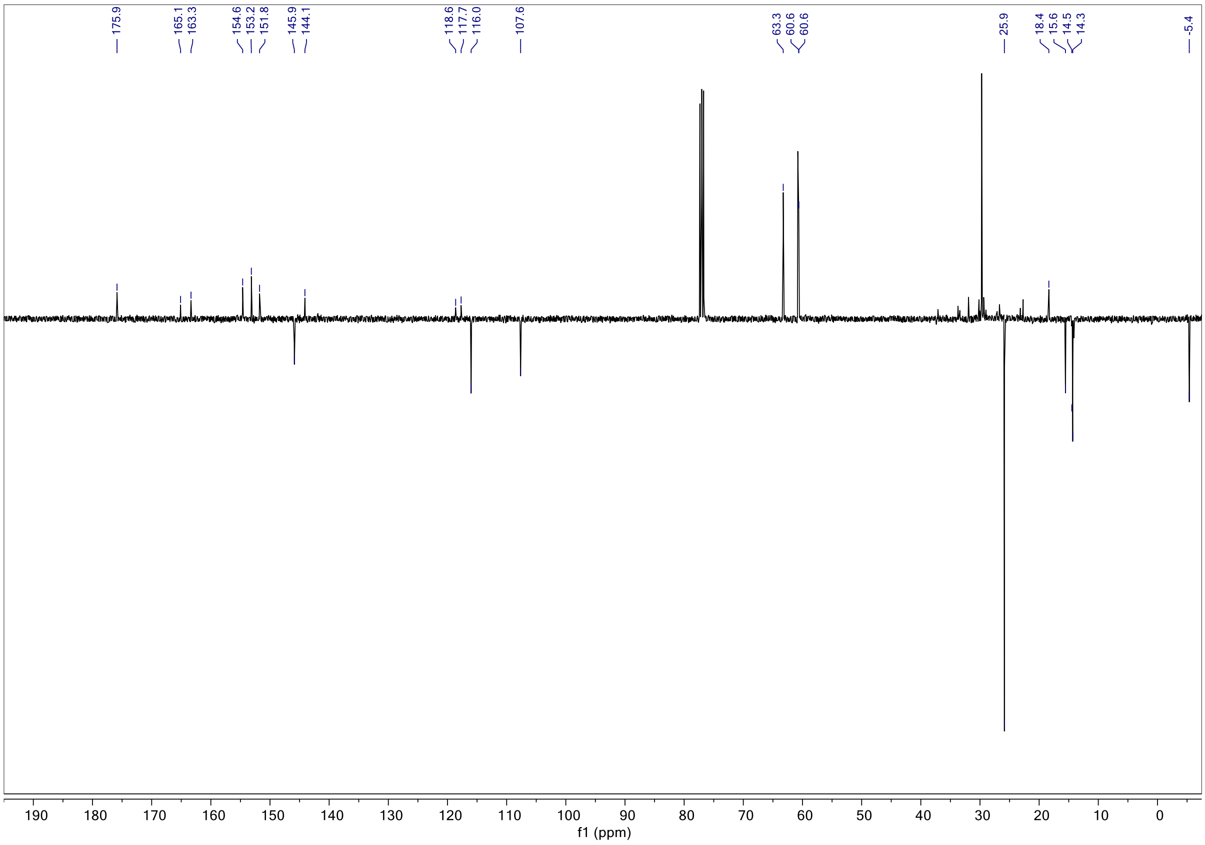

$^{13}C\{^{1}H\}$-APT NMR (100 MHz, $CDCl_3$)

Ethyl 2-(3-(4-(((tert-butyldimethylsilyl)oxy)methyl)pyridin-2-yl)thioureido)-4,5-dimethylthiophene-3-carboxylate (9)

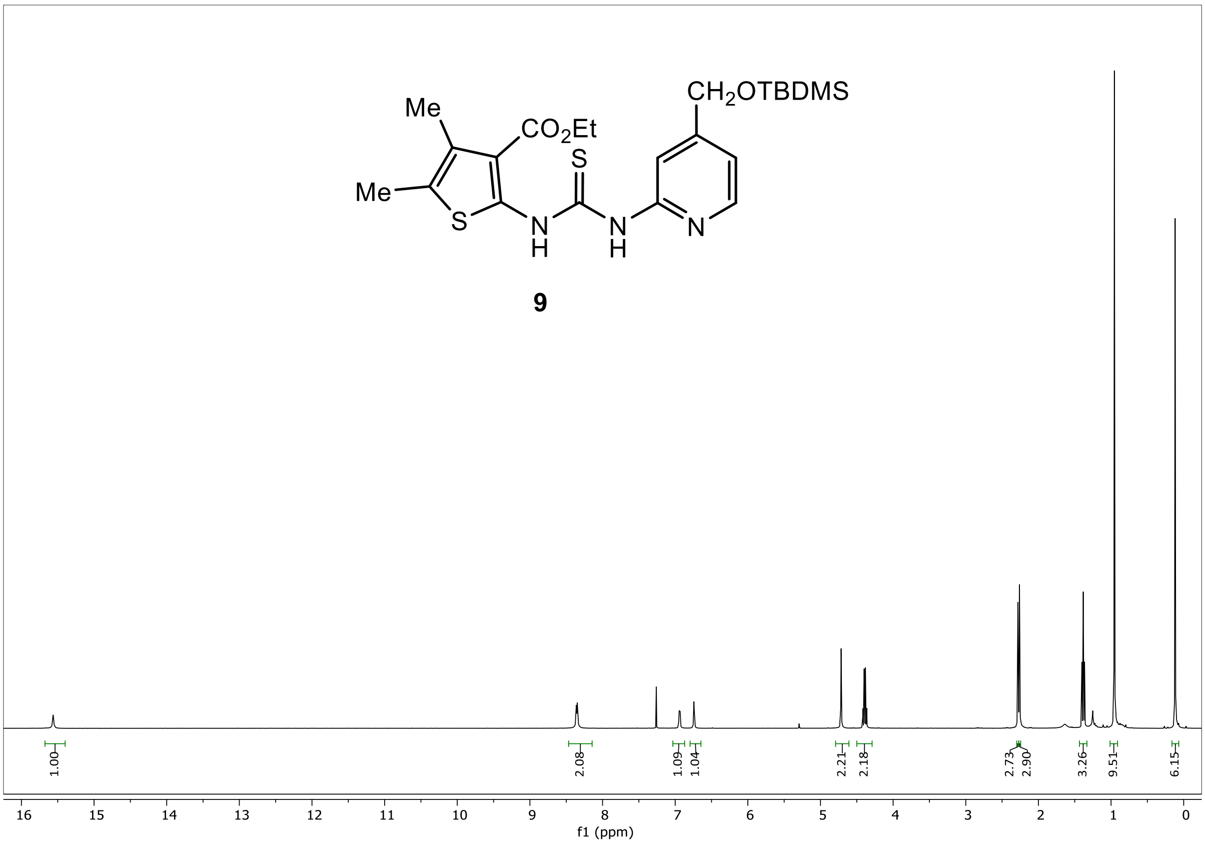

^{1}H-NMR (400 MHz, $CDCl_3$)

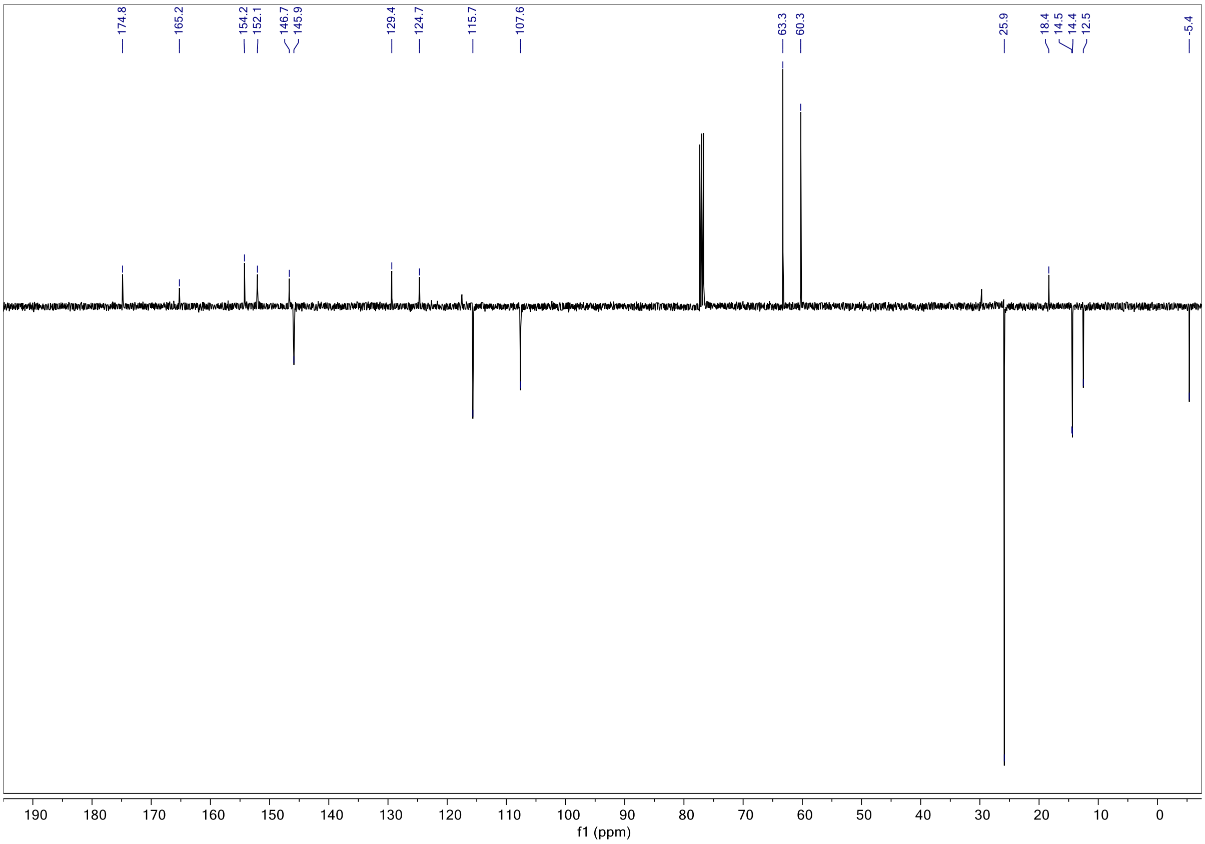

$^{13}C\{^{1}H\}$-APT NMR (100 MHz, $CDCl_3$)

Ethyl 2-(3-(4-chloropyridin-2-yl)thioureido)-4,5-dimethylthiophene-3-carboxylate (13)

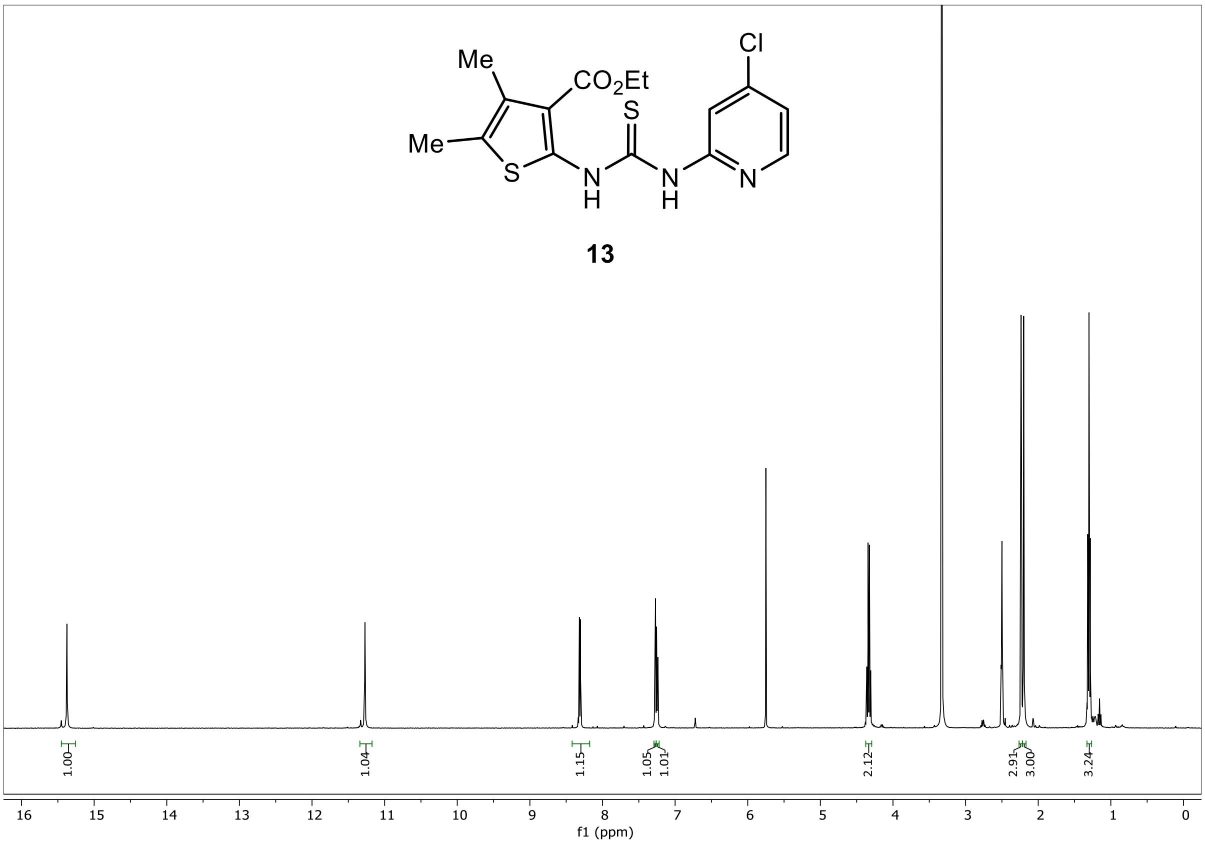

^{1}H-NMR (400 MHz, DMSO-d_6)

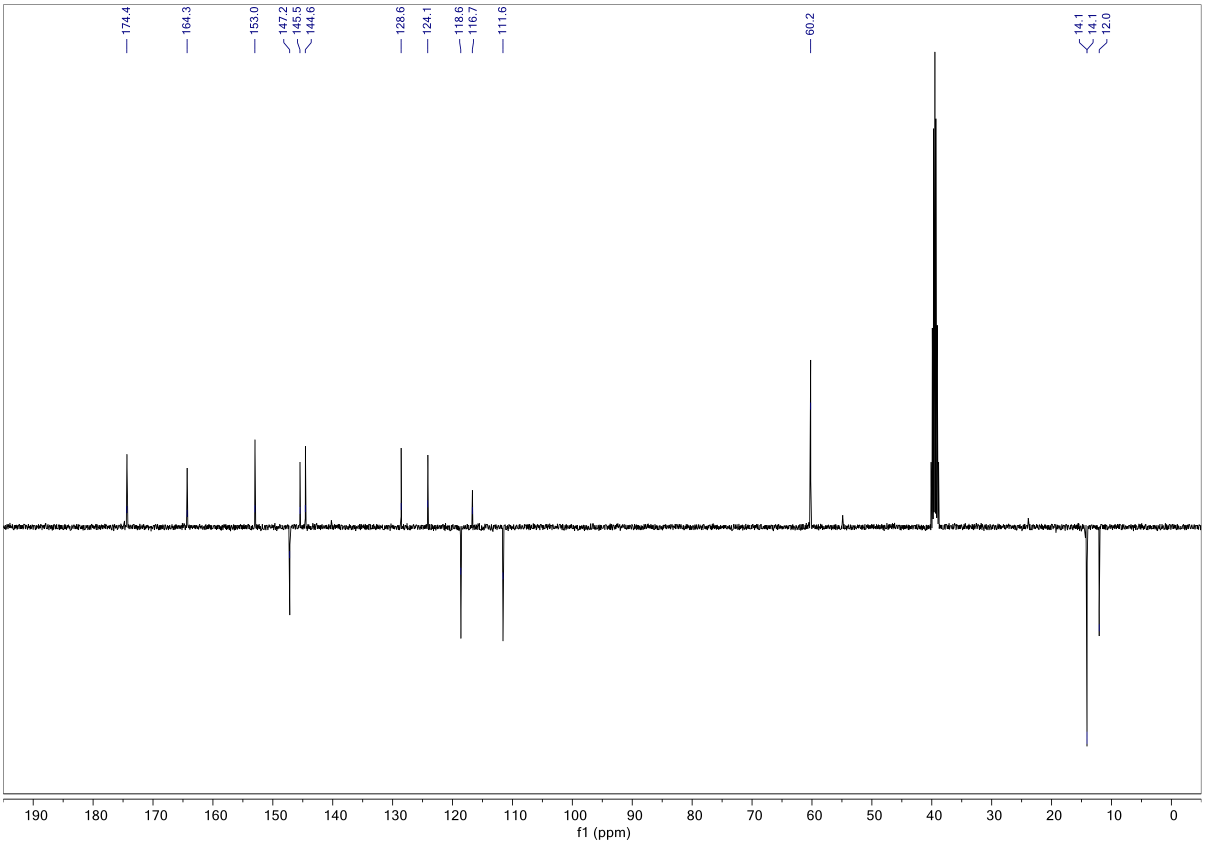

^{13}C{^{1}H}-APT NMR (100 MHz, DMSO-d_6)

Ethyl 2-(3-(4-methoxypyridin-2-yl)thioureido)-4,5-dimethylthiophene-3-carboxylate (14)

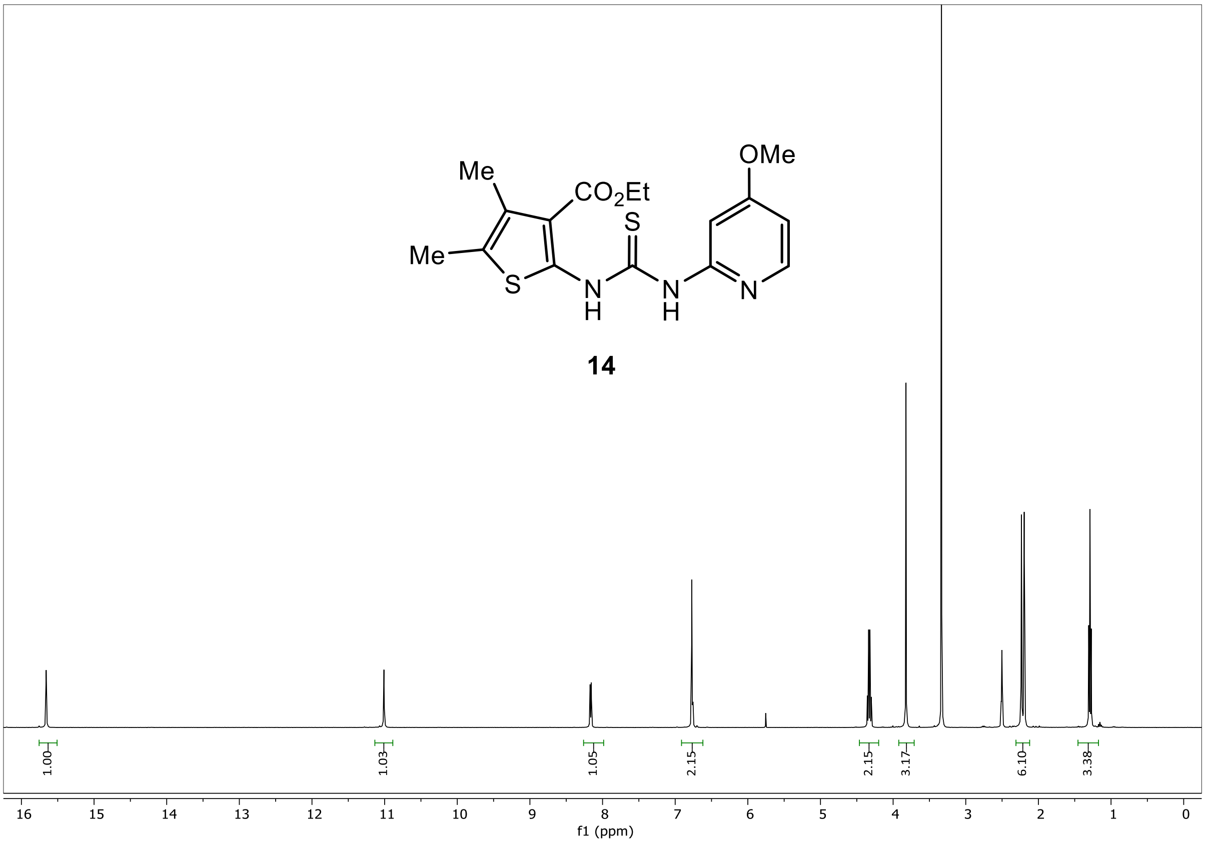

^{1}H-NMR (400 MHz, DMSO-d_6)

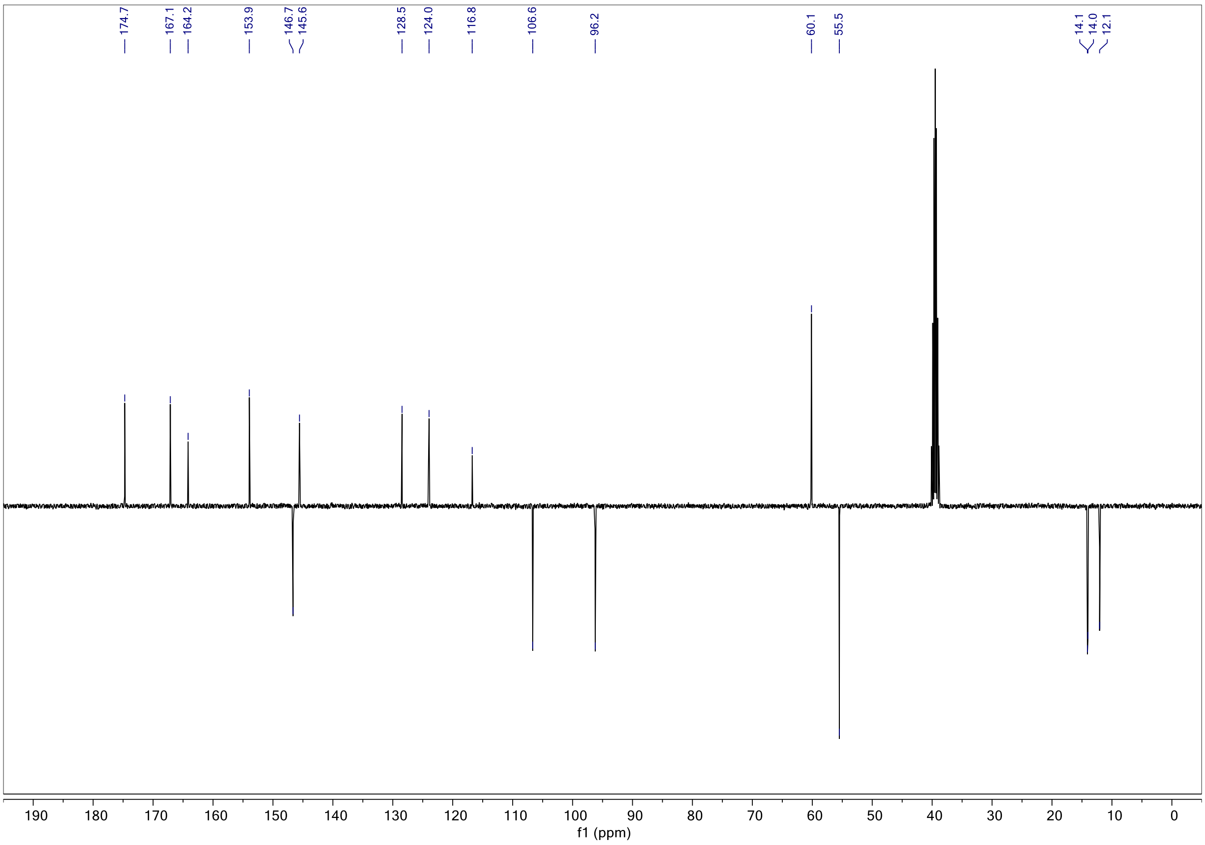

^{13}C{^{1}H} NMR (100 MHz, DMSO-d_6)

Ethyl 2-(3-(4-cyanopyridin-2-yl)thioureido)-4,5-dimethylthiophene-3-carboxylate (15)

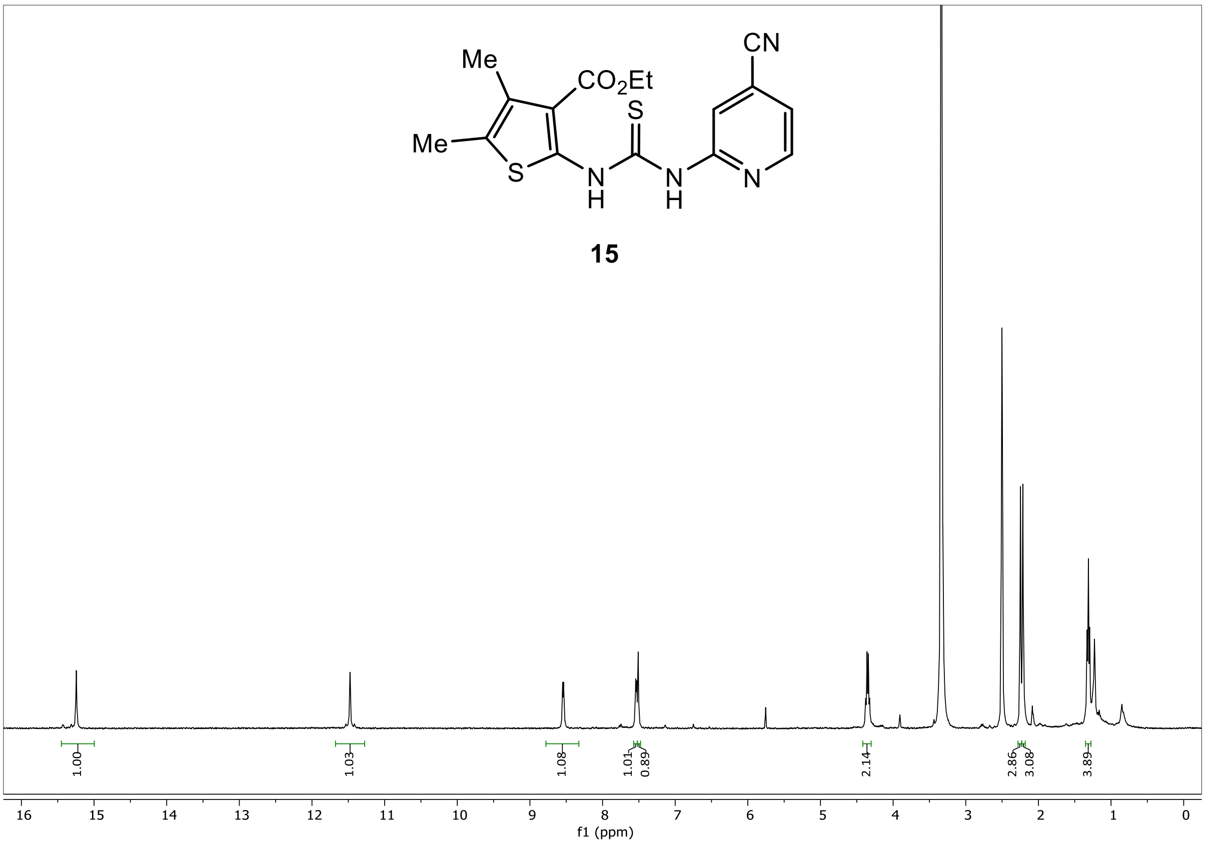

^{1}H-NMR (400 MHz, DMSO-d_6)

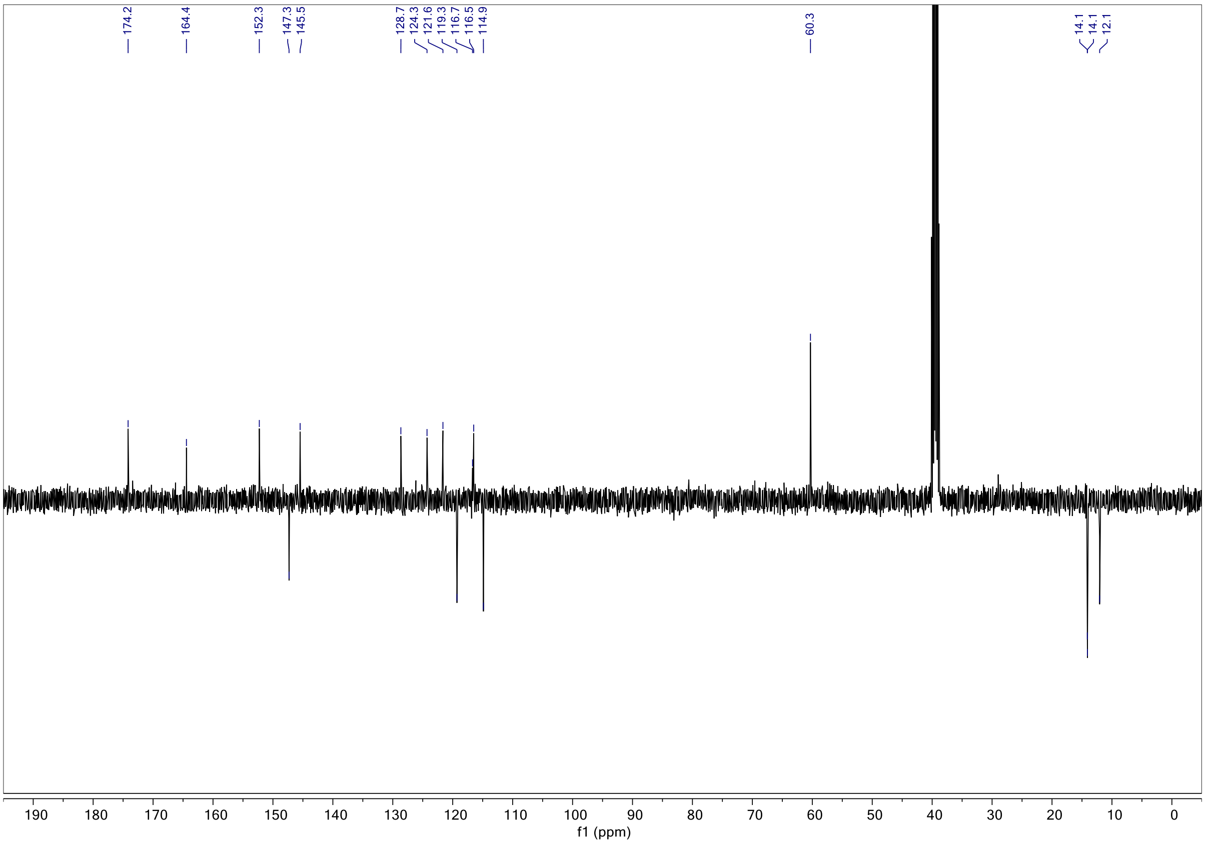

^{13}C{^{1}H}-APT NMR (100 MHz, DMSO-d_6)

Ethyl 5-methyl-3-(4-methylpyridin-2-yl)-4-oxo-2-thioxo-1,2,3,4-tetrahydrothieno[2,3-d] pyrimidine-6-carboxylate (IVc)

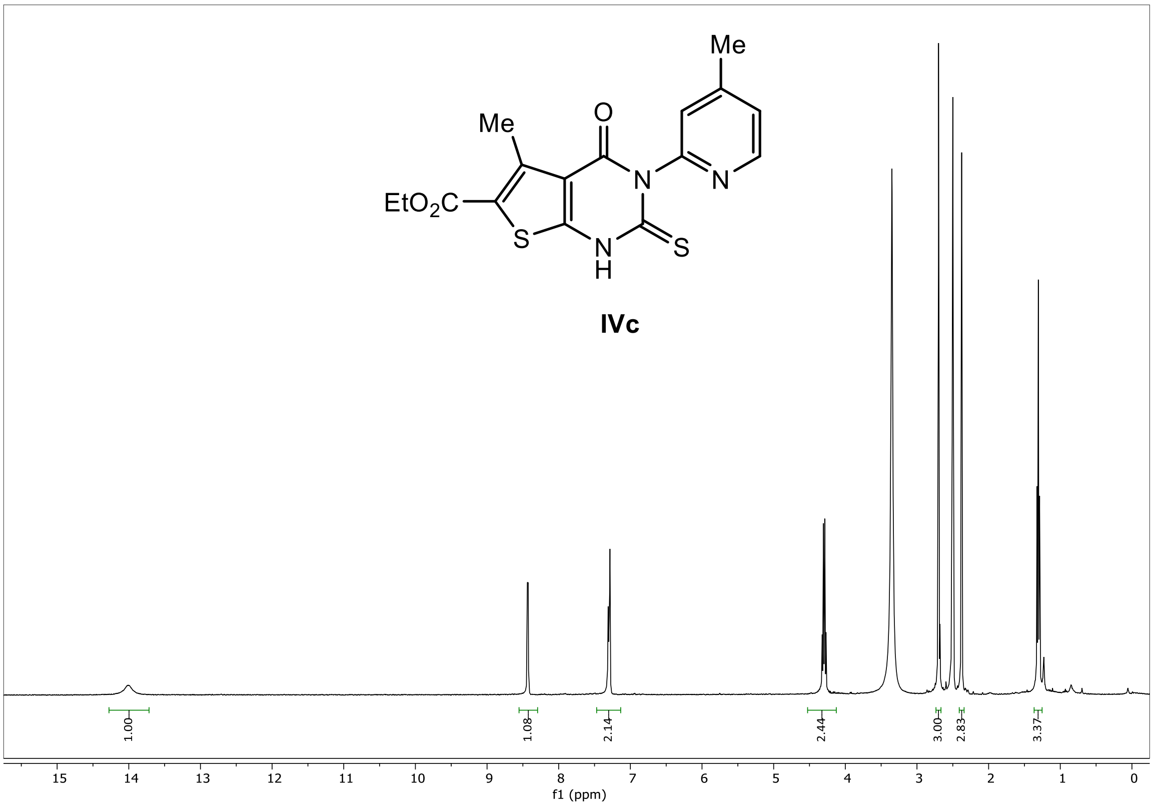

^{1}H-NMR (400 MHz, DMSO-d_6)

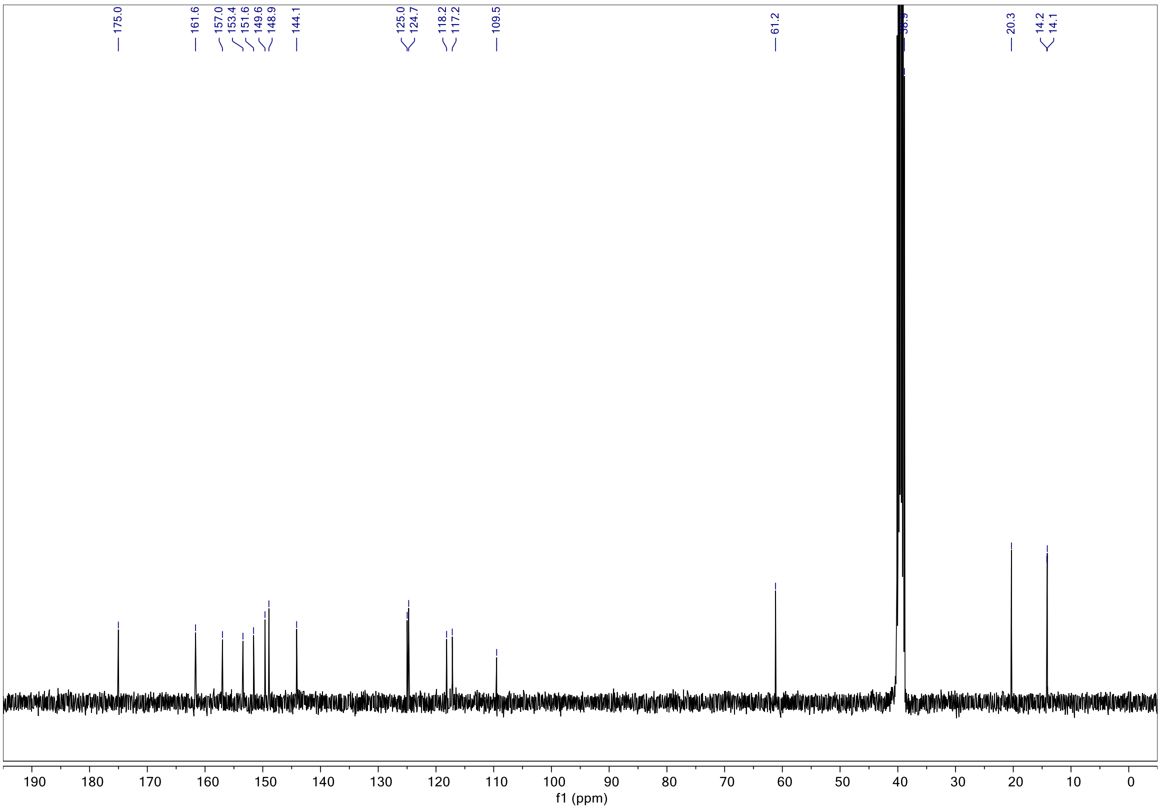

^{13}C{^{1}H}-NMR (100 MHz, DMSO-d_6)

Ethyl 3-(4-(((*tert*-butyldimethylsilyl)oxy)methyl)pyridin-2-yl)-5-methyl-4-oxo-2-thioxo-1,2,3,4-tetrahydrothieno[2,3-d]pyrimidine-6-carboxylate (16)

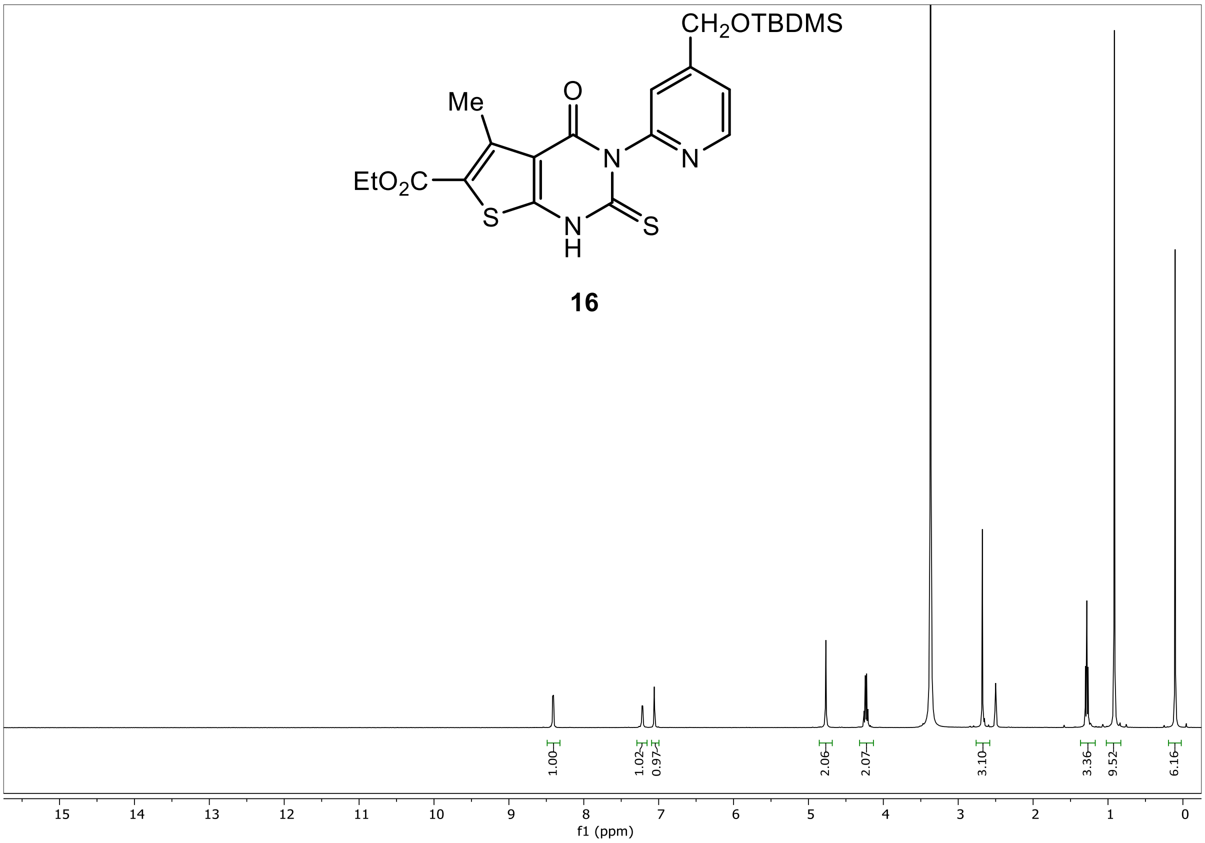

^{1}H-NMR (400 MHz, DMSO-d_6)

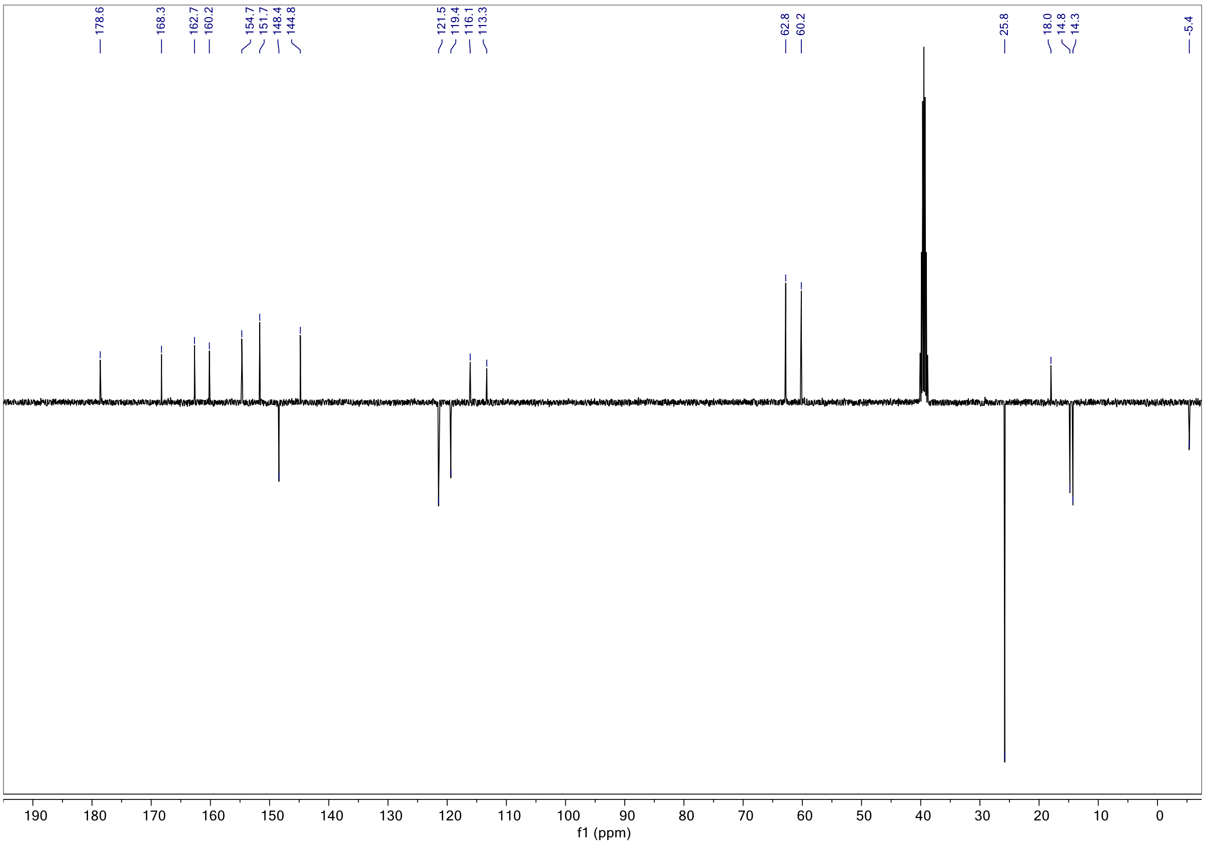

^{13}C{^{1}H}-APT NMR (100 MHz, DMSO-d_6)

3-(4-(((tert-Butyldimethylsilyl)oxy)methyl)pyridin-2-yl)-5,6-dimethyl-2-thioxo-2,3-dihydrothieno[2,3-d]pyrimidin-4(1H)-one (17)

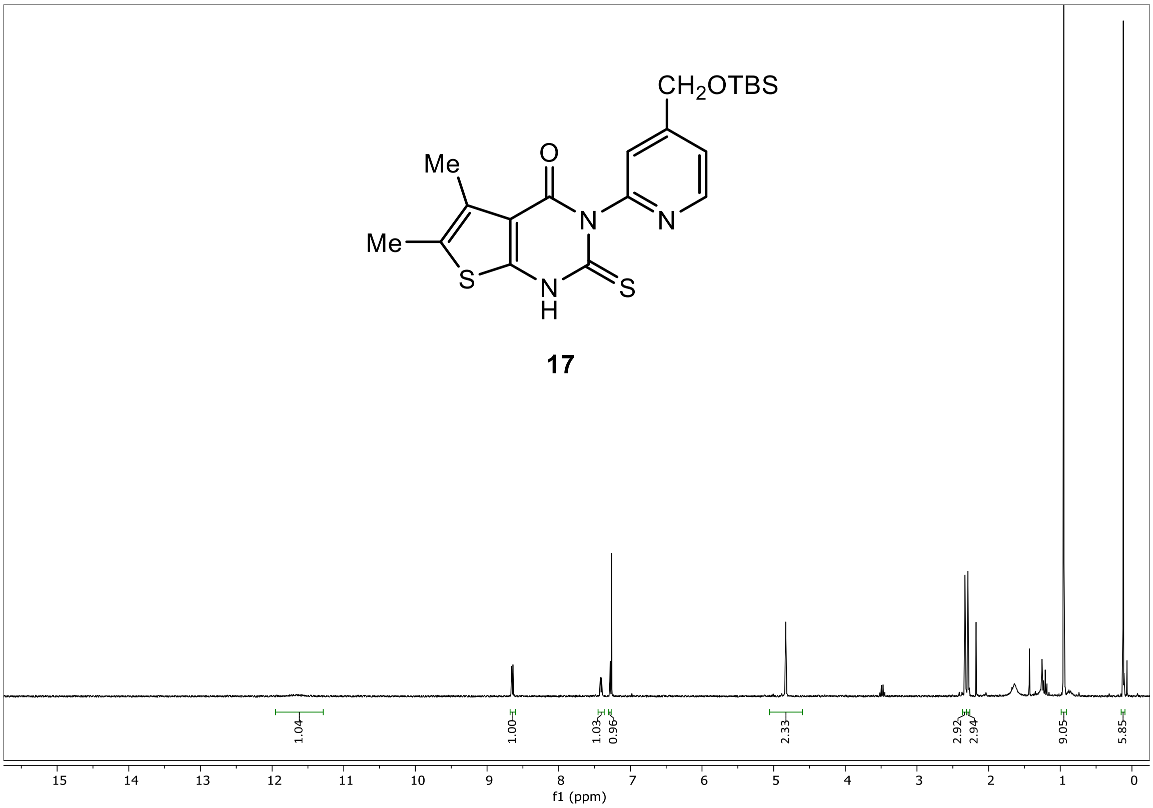

^{1}H-NMR (400 MHz, CDCl$_3$)

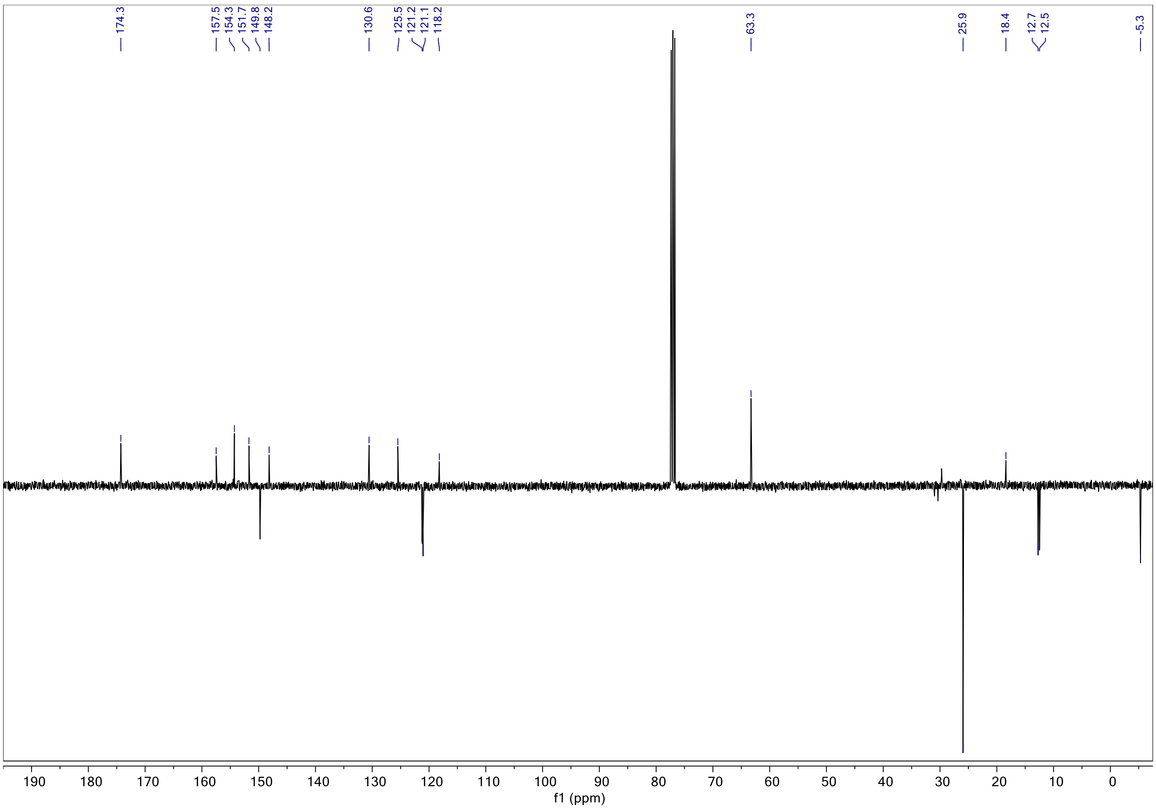

^{13}C{^{1}H}-APT NMR (100 MHz, CDCl$_3$)

3-(4-Chloropyridin-2-yl)-5,6-dimethyl-2-thioxo-2,3-dihydrothieno[2,3-d]pyrimidin-4(1H)-one (IVe)

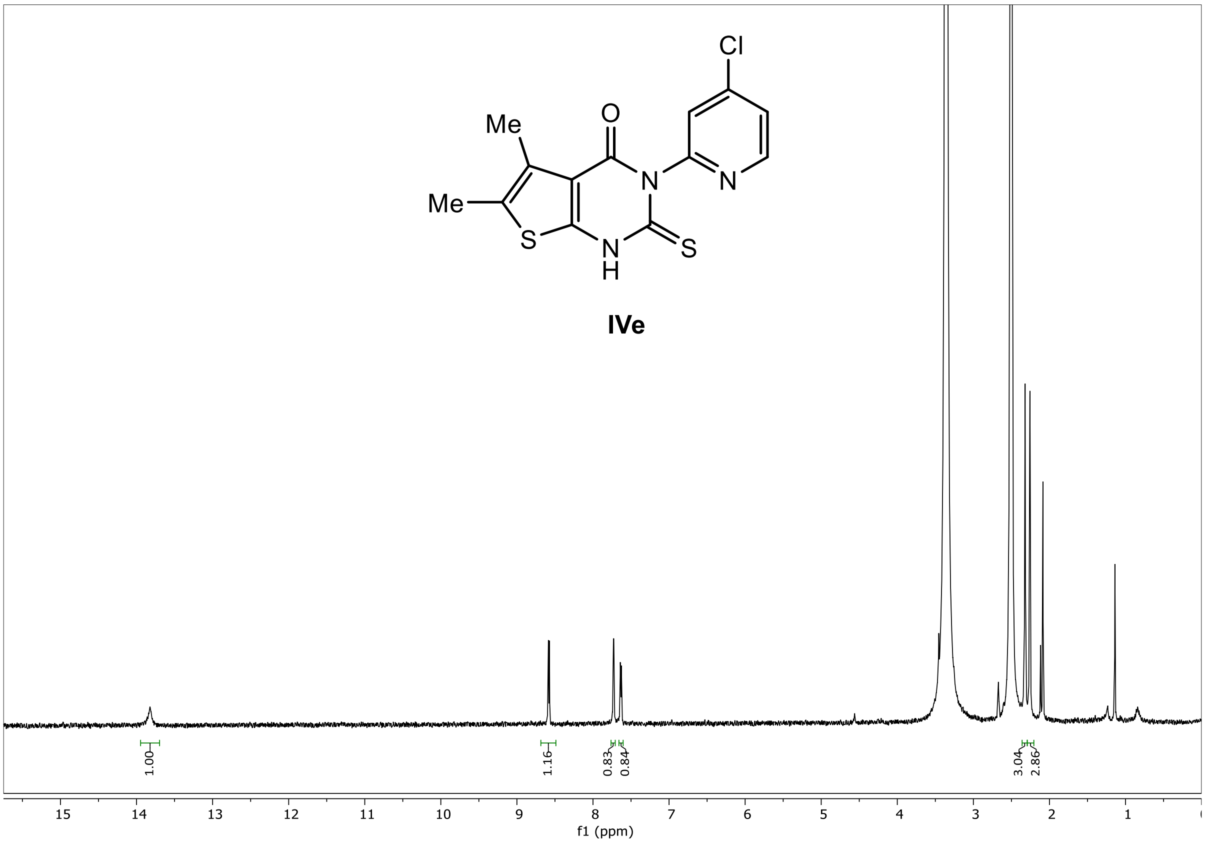

^{1}H-NMR (400 MHz, DMSO-d_6)

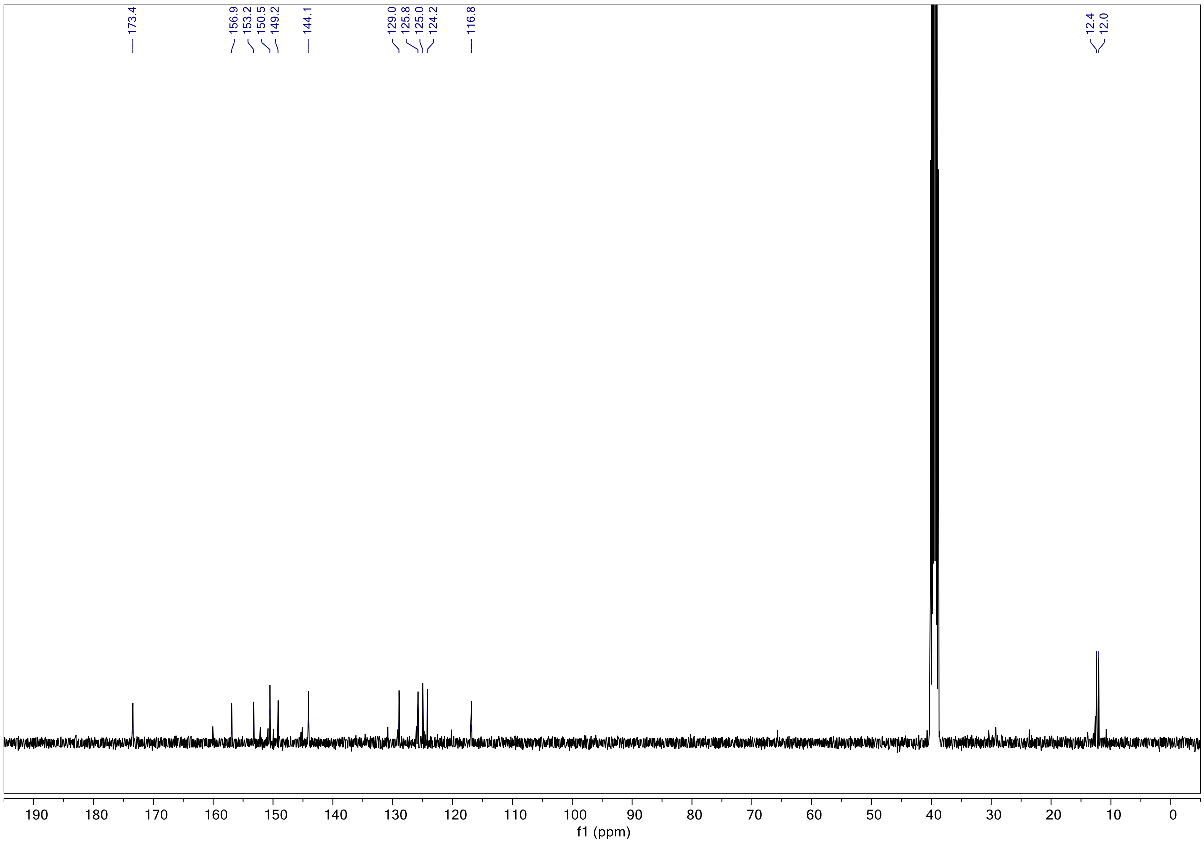

^{13}C{^{1}H}-NMR (100 MHz, DMSO-d_6)

3-(4-Methoxypyridin-2-yl)-5,6-dimethyl-2-thioxo-2,3-dihydrothieno[2,3-d]pyrimidin-4(1H)-one (IVf)

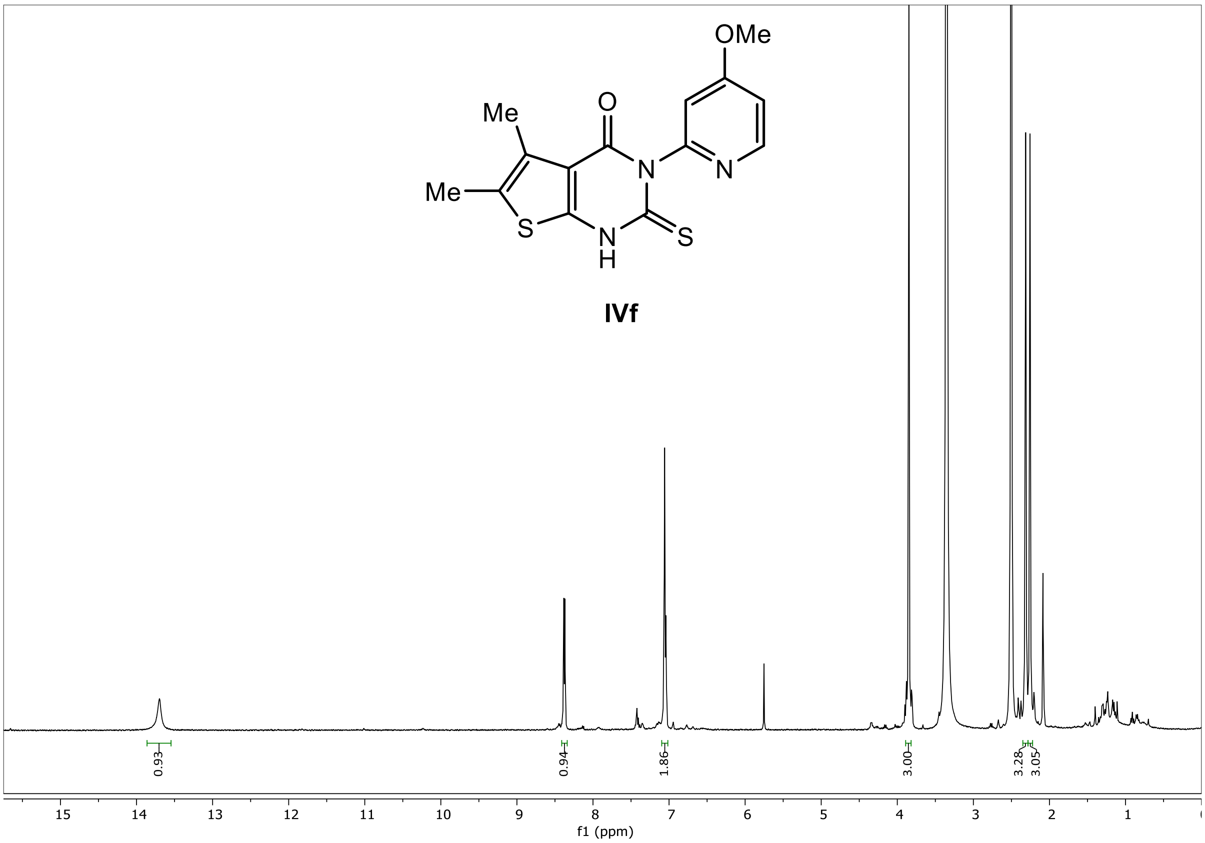

^{1}H-NMR (400 MHz, DMSO-d_6)

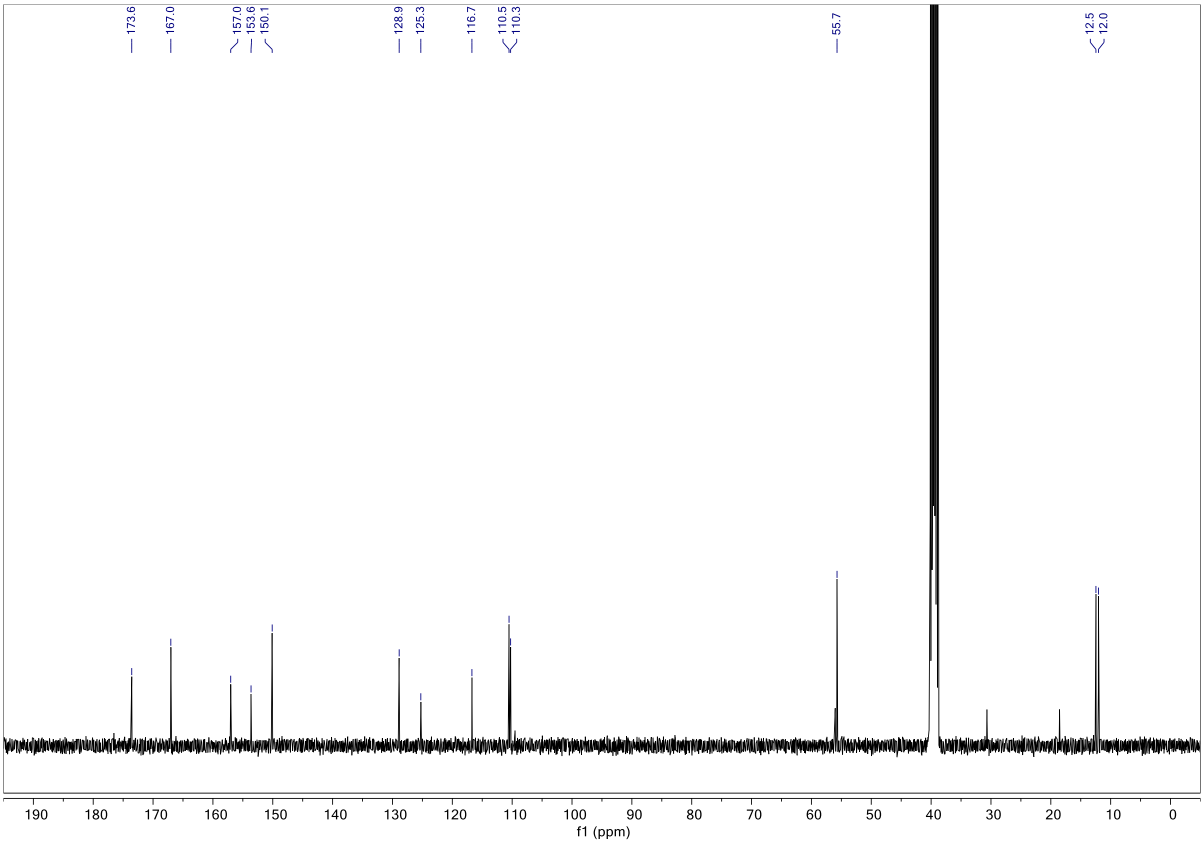

^{13}C{^{1}H}-NMR (100 MHz, DMSO-d_6)

2-(5,6-Dimethyl-4-oxo-2-thioxo-1,4-dihydrothieno[2,3-d]pyrimidin-3(2H)-yl)isonicotinonitrile (IVg)

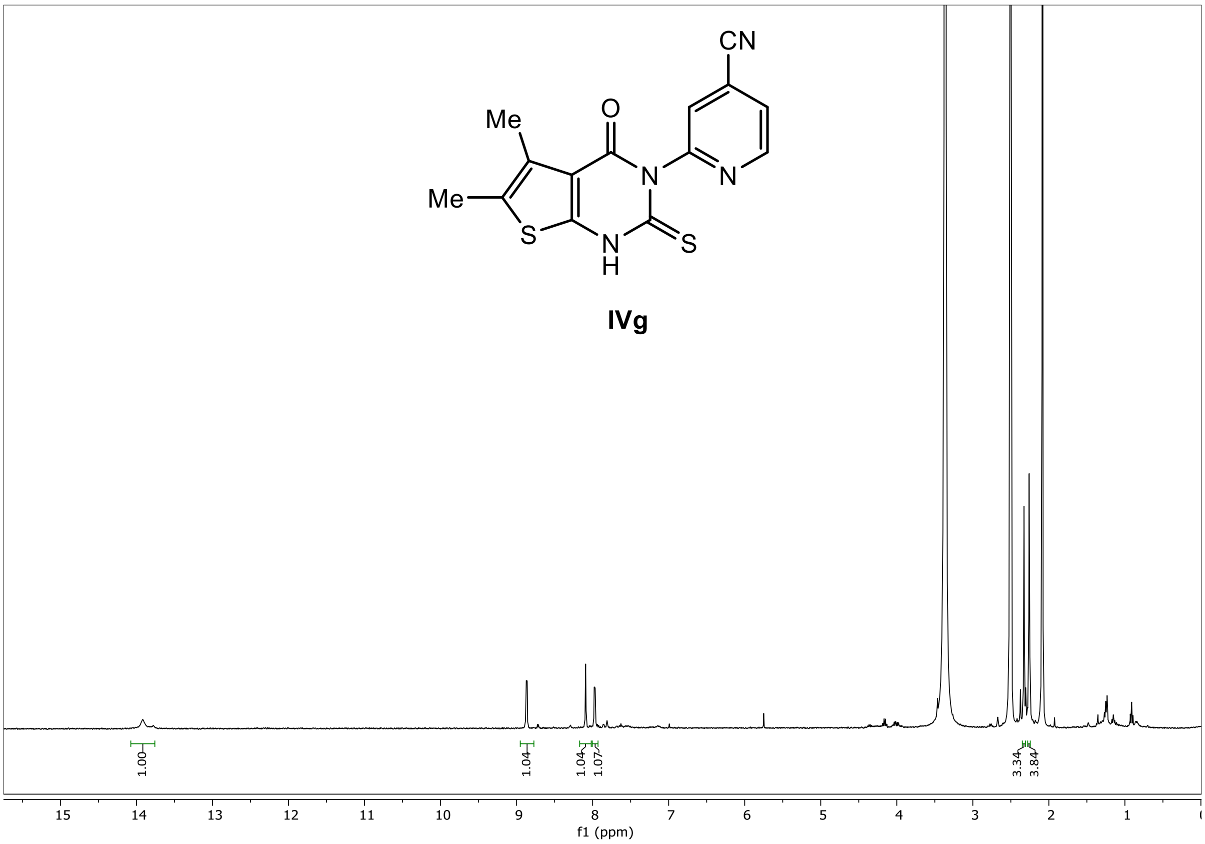

^{1}H-NMR (400 MHz, DMSO-d_6)

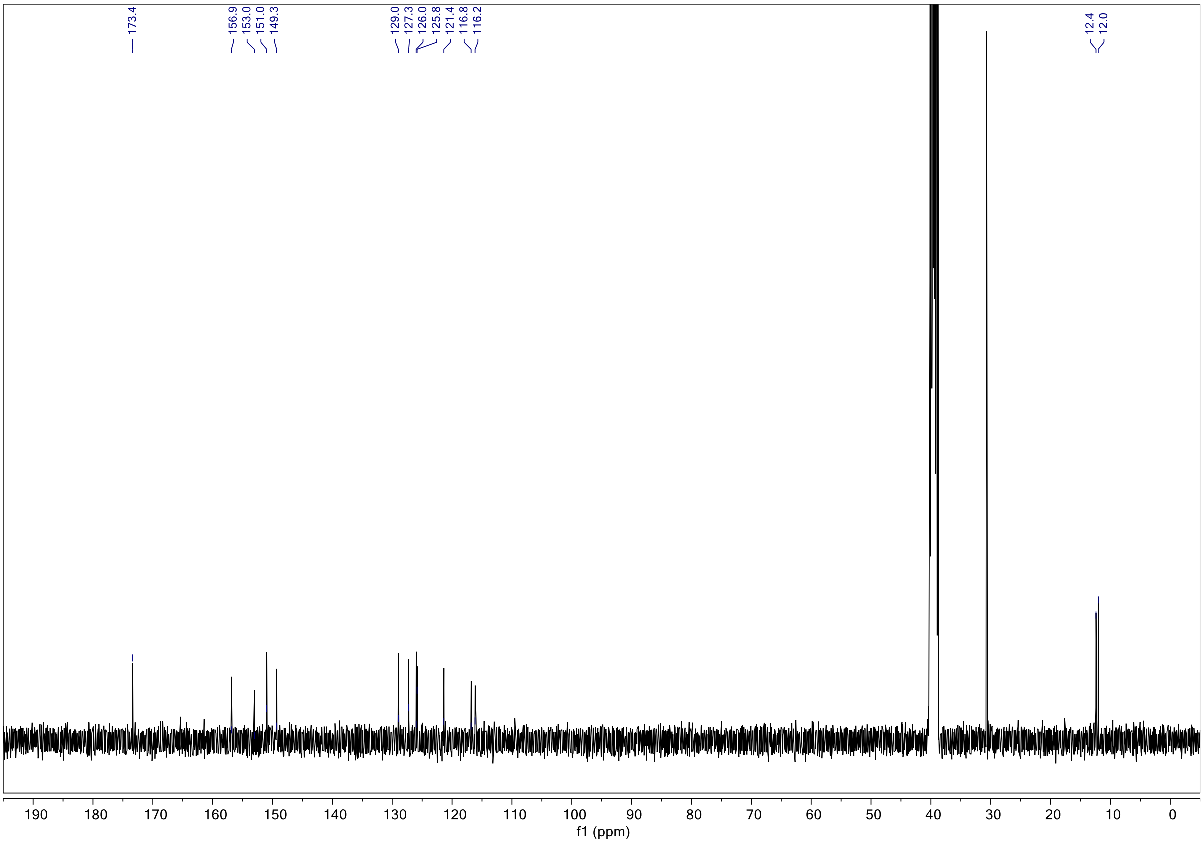

^{13}C{^{1}H}-NMR (100 MHz, DMSO-d_6)

3-(4-(((*tert*-Butyldimethylsilyl)oxy)methyl)pyridin-2-yl)-6-(hydroxymethyl)-5-methyl-2-thioxo-2,3-dihydrothieno[2,3-d]pyrimidin-4(1H)-one (18)

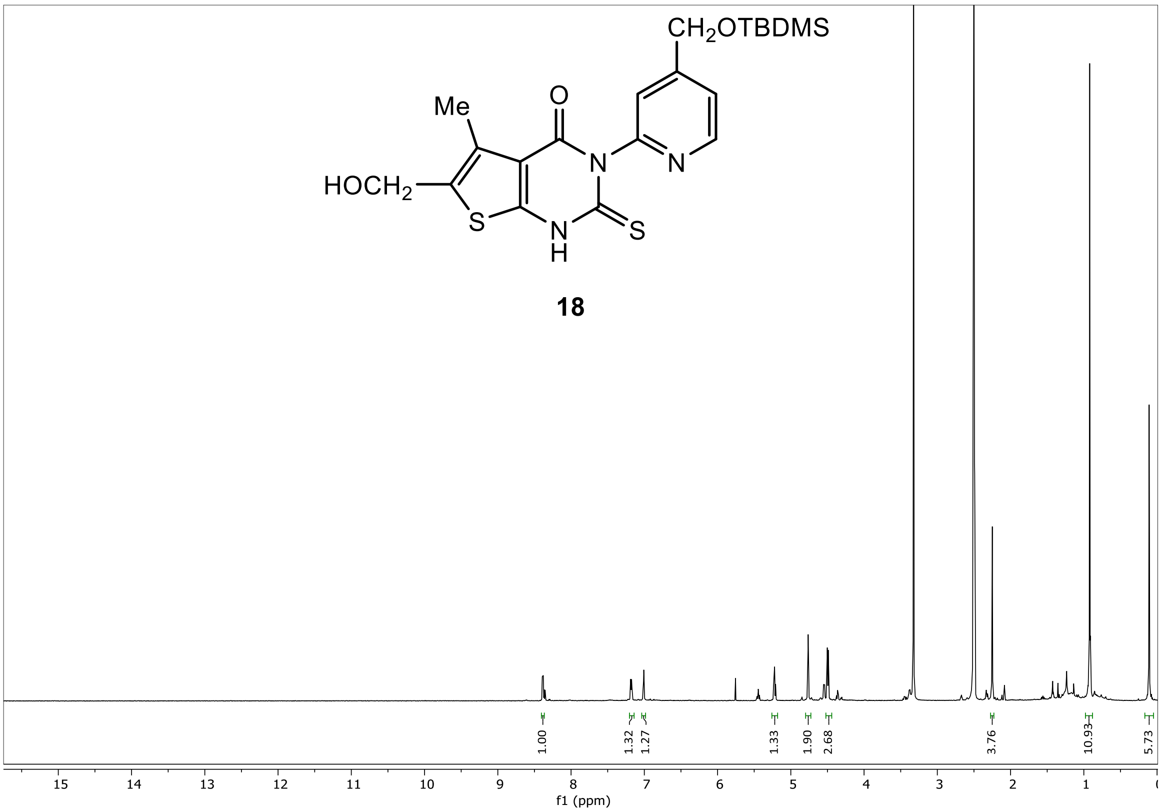

^{1}H-NMR (400 MHz, DMSO-d_6)

6-(Hydroxymethyl)-5-methyl-3-(4-methylpyridin-2-yl)-2-thioxo-2,3-dihydrothieno[2,3-d]pyrimidin-4(1H)-one (IVa)

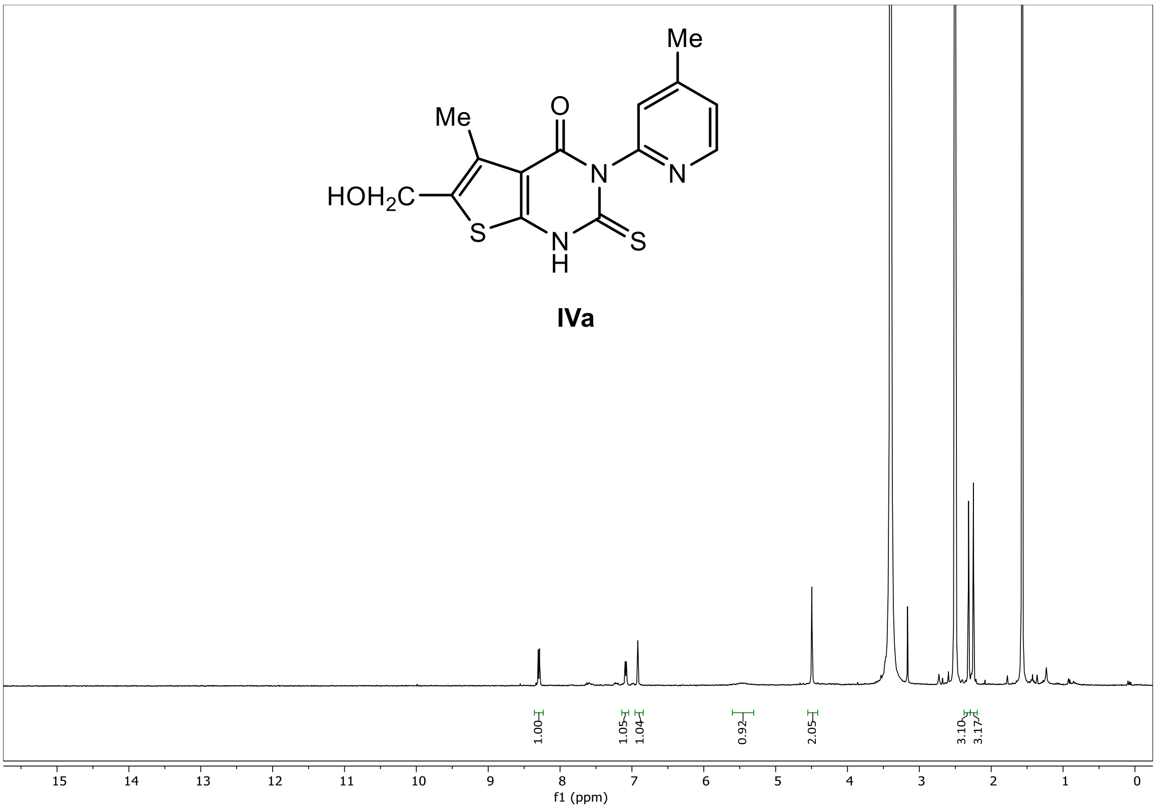

^{1}H-NMR (300 MHz, DMSO-d_6)

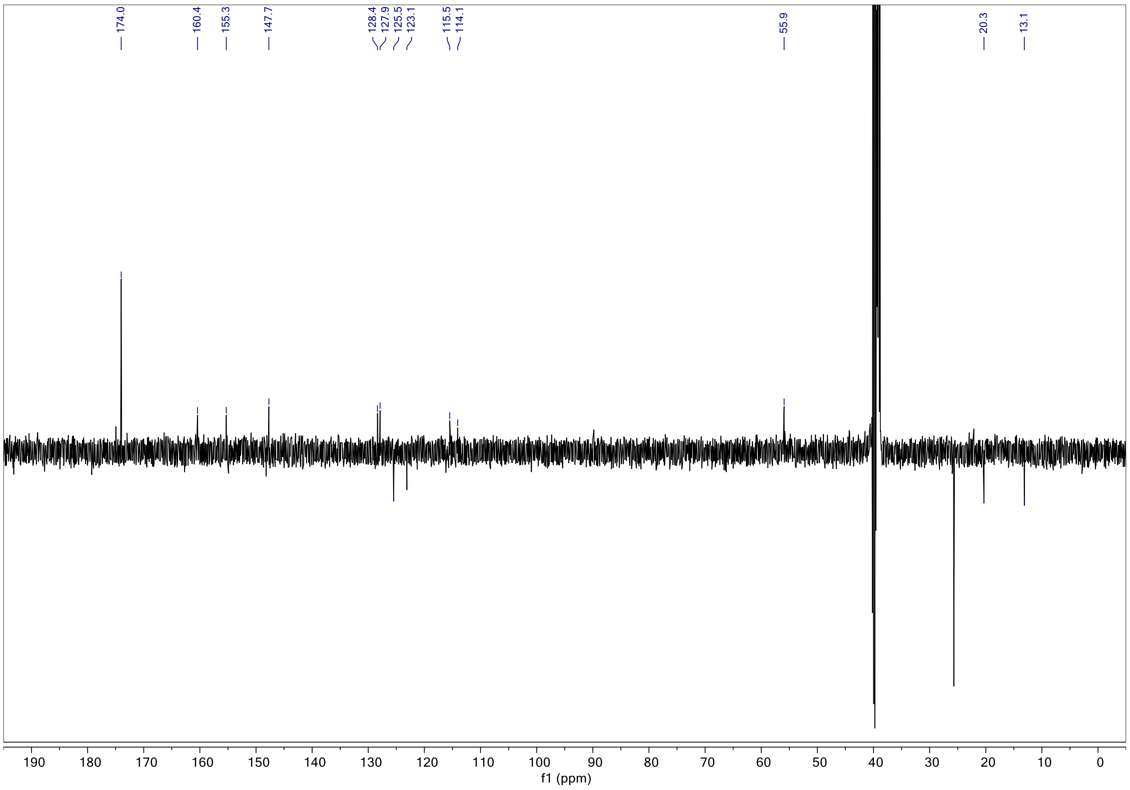

$^{13}C\{^{1}H\}$-APT NMR (100 MHz, DMSO-d_6)

3-(4-(Hydroxymethyl)pyridin-2-yl)-5,6-dimethyl-2-thioxo-2,3-dihydrothieno[2,3-d]pyrimidin-4(1H)-one (IVb)

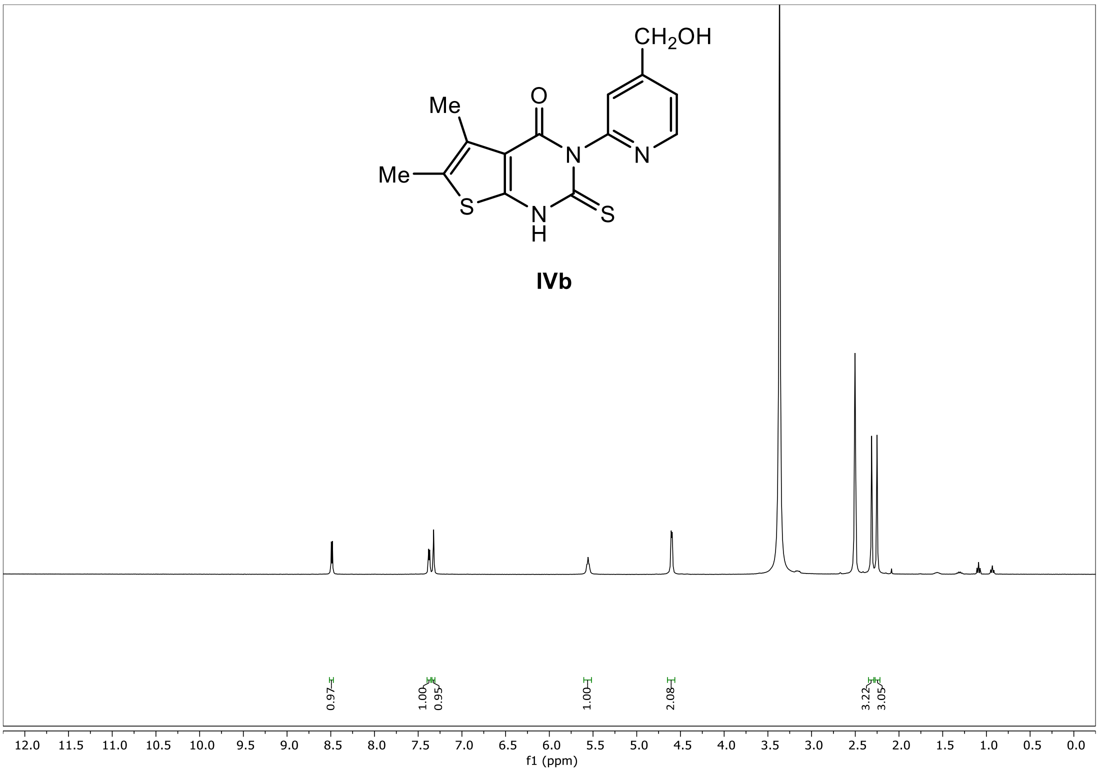

^{1}H-NMR (400 MHz, DMSO-d_6)

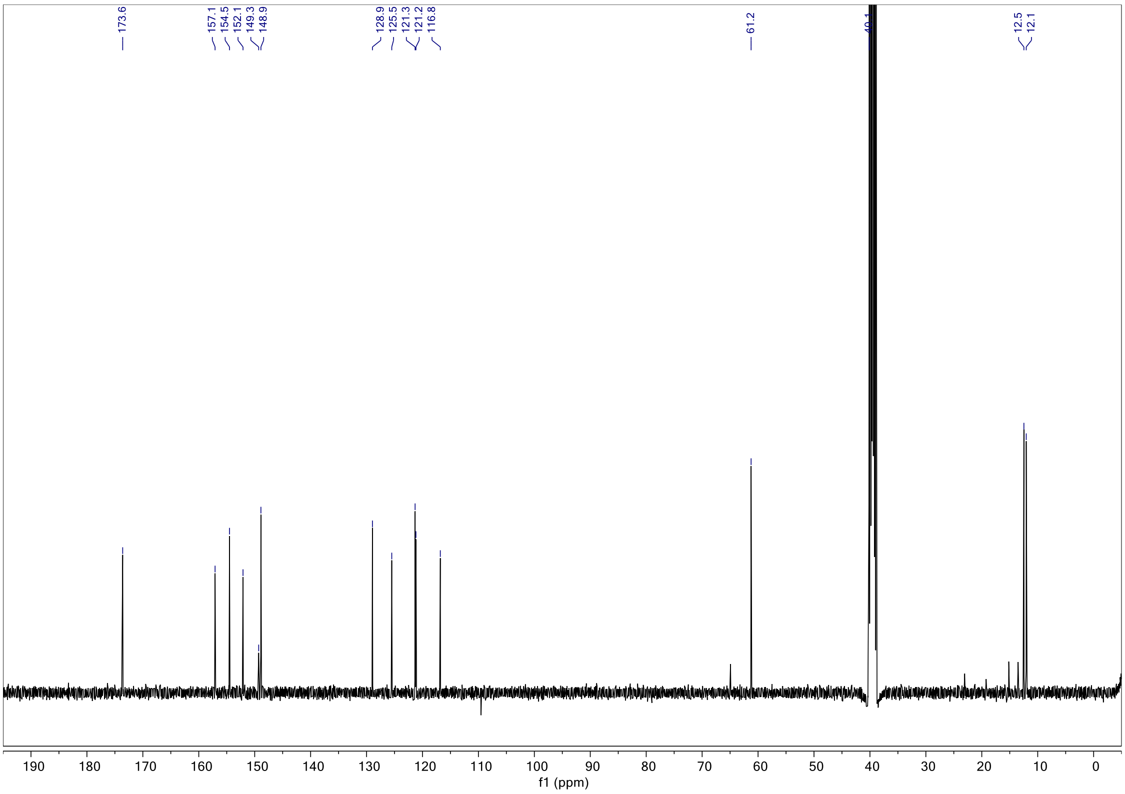

^{13}C{^{1}H} NMR (100 MHz, DMSO-d_6)

6-(Hydroxymethyl)-3-(4-(hydroxymethyl)pyridin-2-yl)-5-methyl-2-thioxo-2,3-dihydrothieno[2,3-d]pyrimidin-4(1H)-one (IVd)

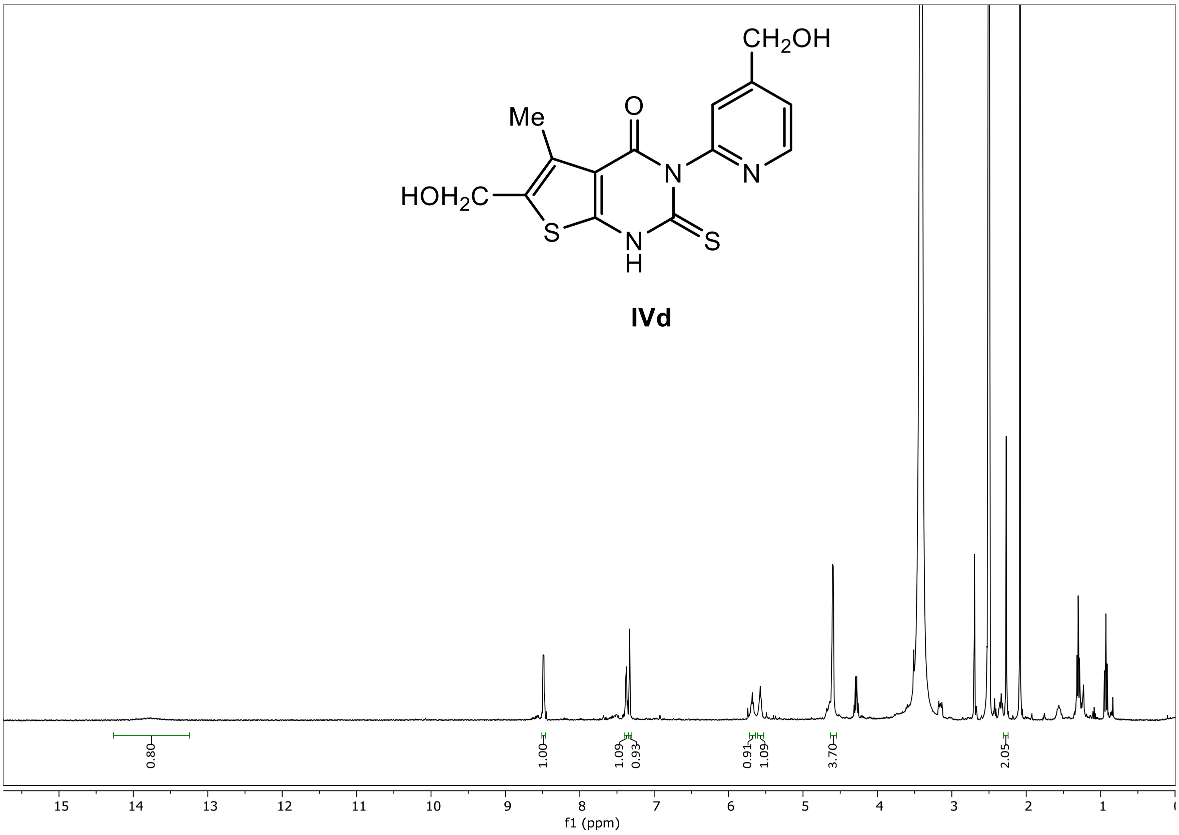

^{1}H-NMR (400 MHz, DMSO-d_6)

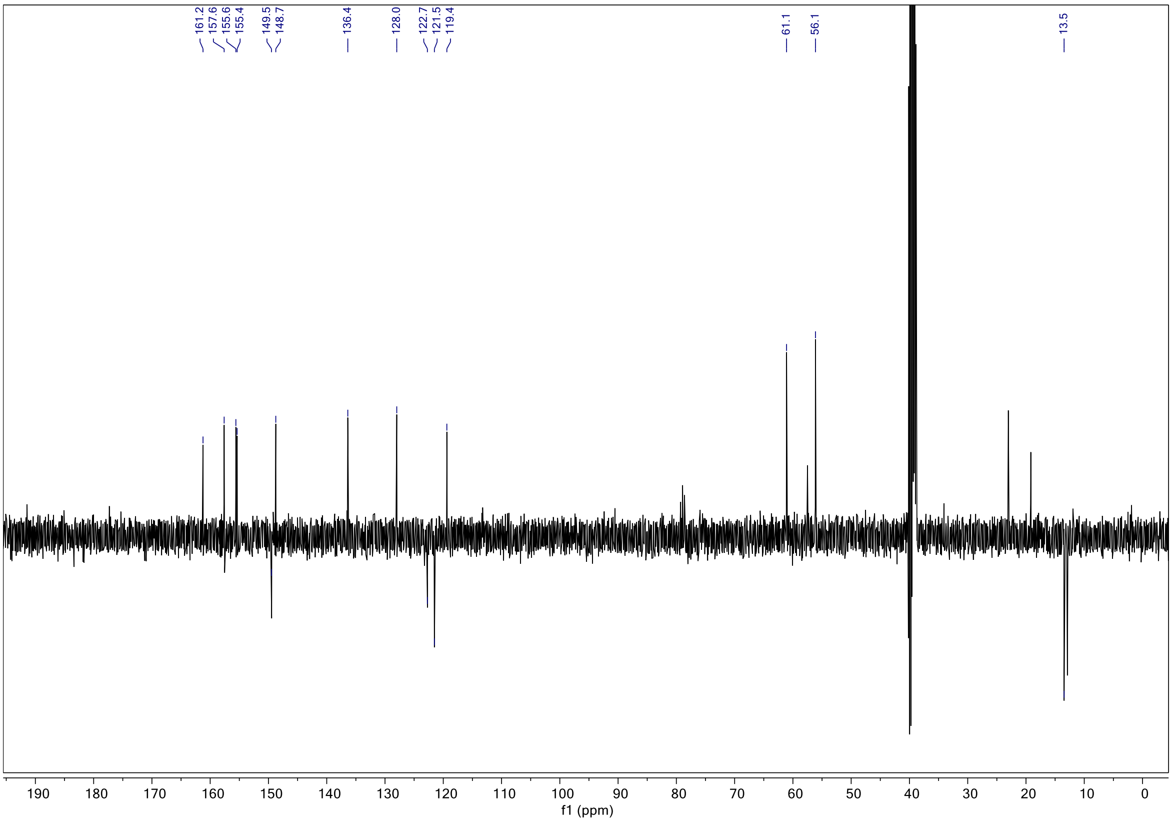

^{13}C{^{1}H}-APT NMR (100 MHz, DMSO-d_6)

Ethyl 2-(benzylthio)-5-methyl-3-(4-methylpyridin-2-yl)-4-oxo-3,4-dihydrothieno[2,3-d]pyrimidine-6-carboxylate (IVh)

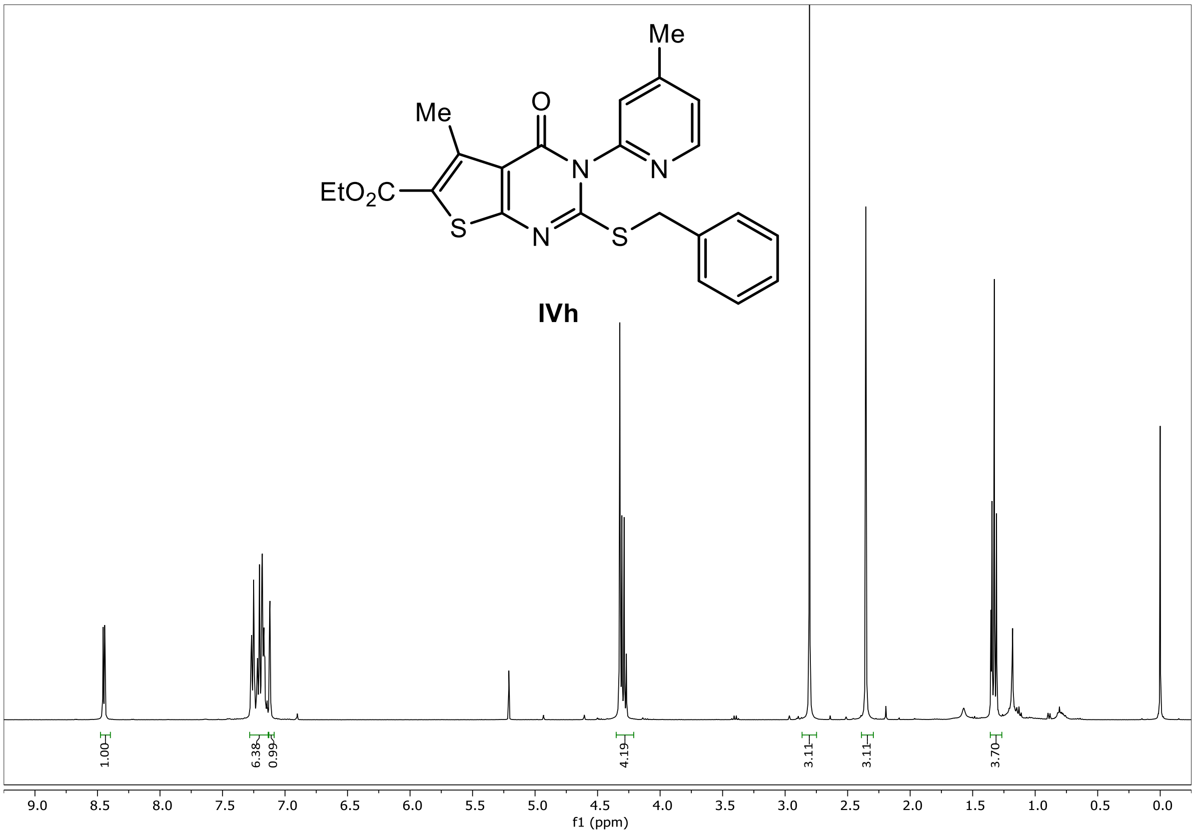

^{1}H-NMR (400 MHz, $CDCl_3$)

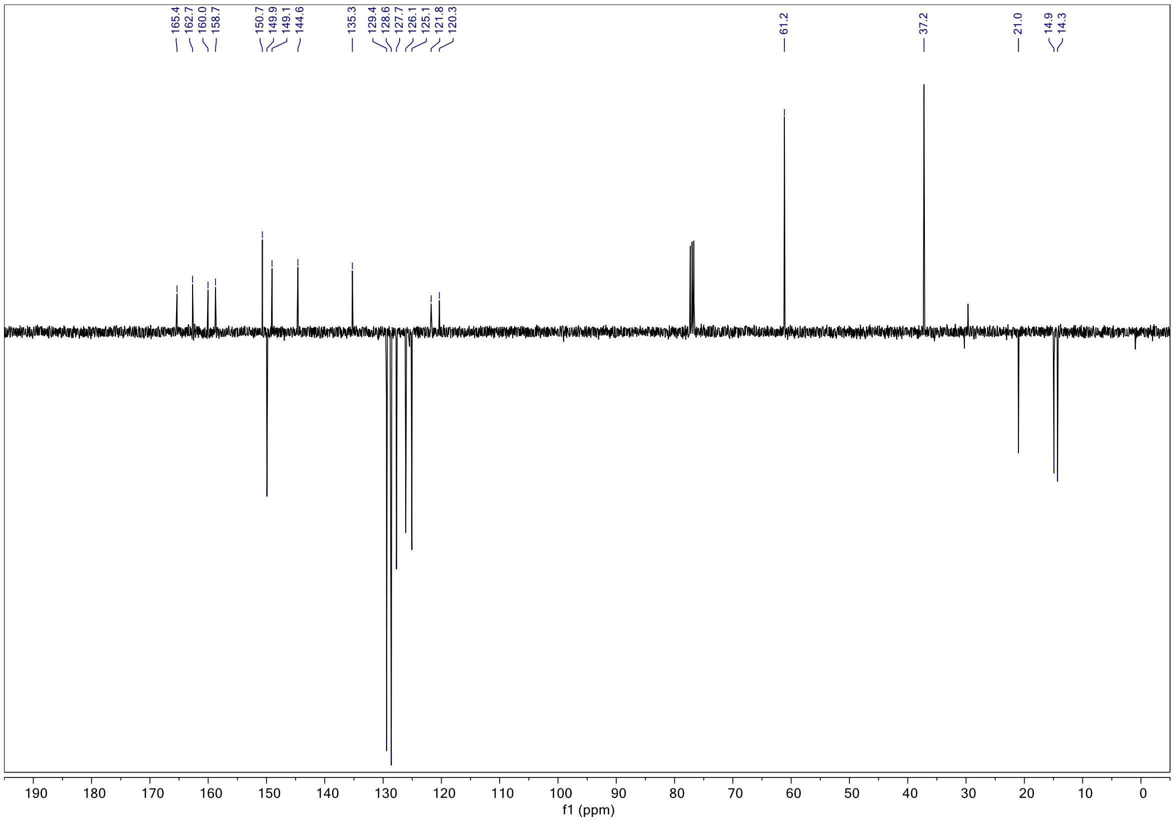

$^{13}C\{^1H\}$-APT NMR (100 MHz, $CDCl_3$)

Ethyl 2-((cyanomethyl)thio)-5-methyl-3-(4-methylpyridin-2-yl)-4-oxo-3,4-dihydrothieno[2,3-d]pyrimidine-6-carboxylate (IVi)

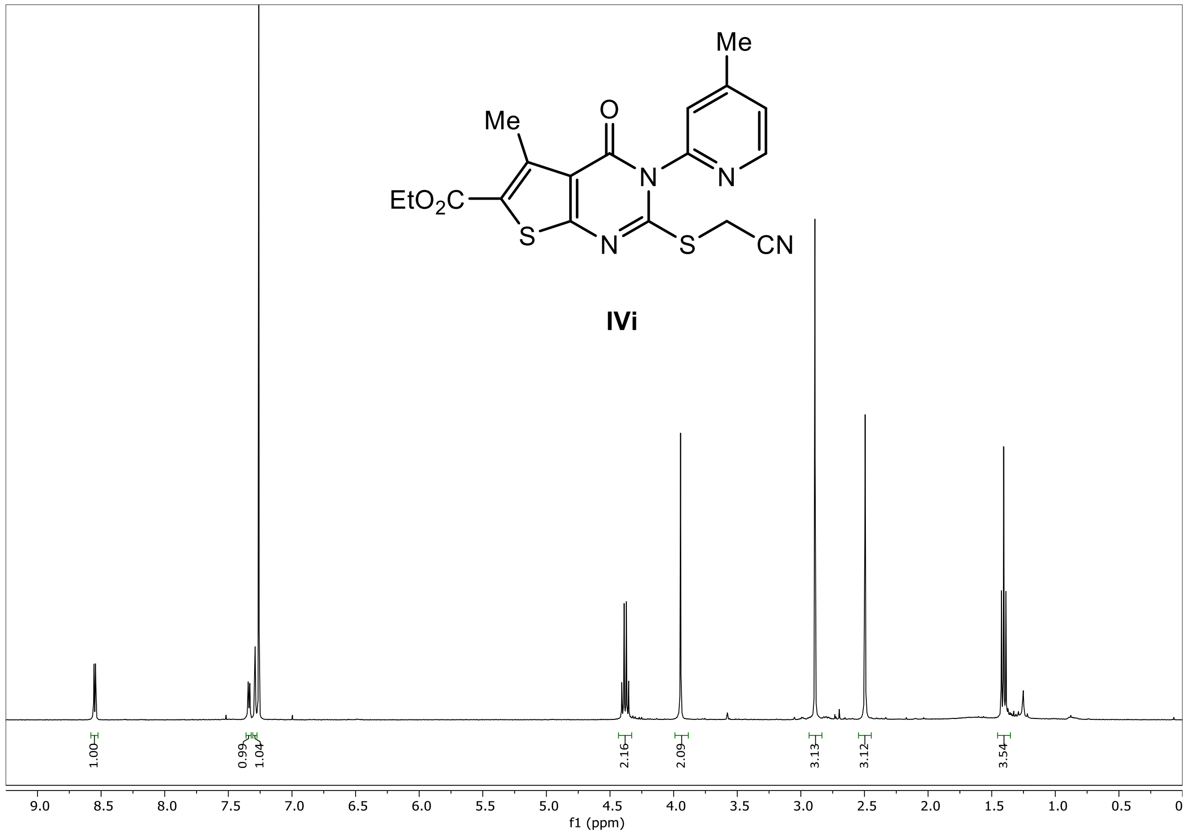

^{1}H-NMR (400 MHz, $CDCl_3$)

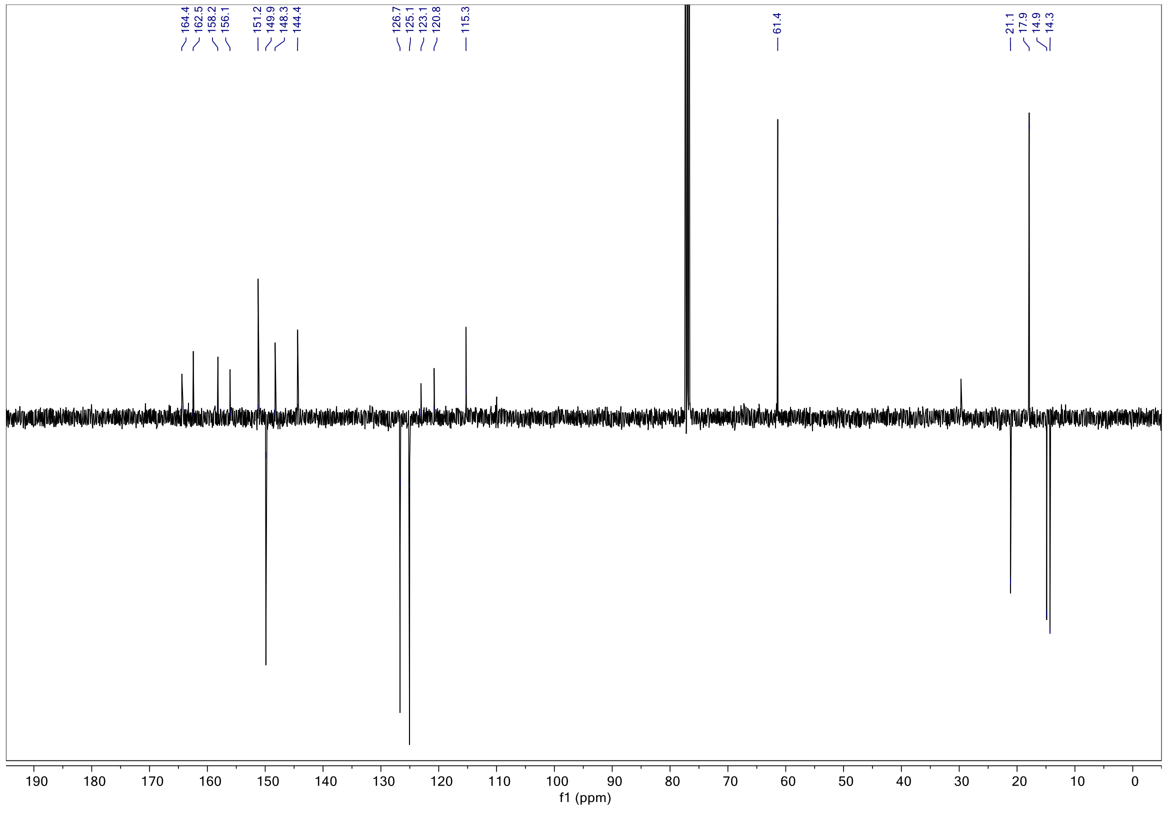

$^{13}C\{^1H\}$-APT NMR (100 MHz, $CDCl_3$)

Ethyl 5-methyl-2-((2-methylallyl)thio)-3-(4-methylpyridin-2-yl)-4-oxo-3,4-dihydrothieno[2,3-d]pyrimidine-6-carboxylate (IVj)

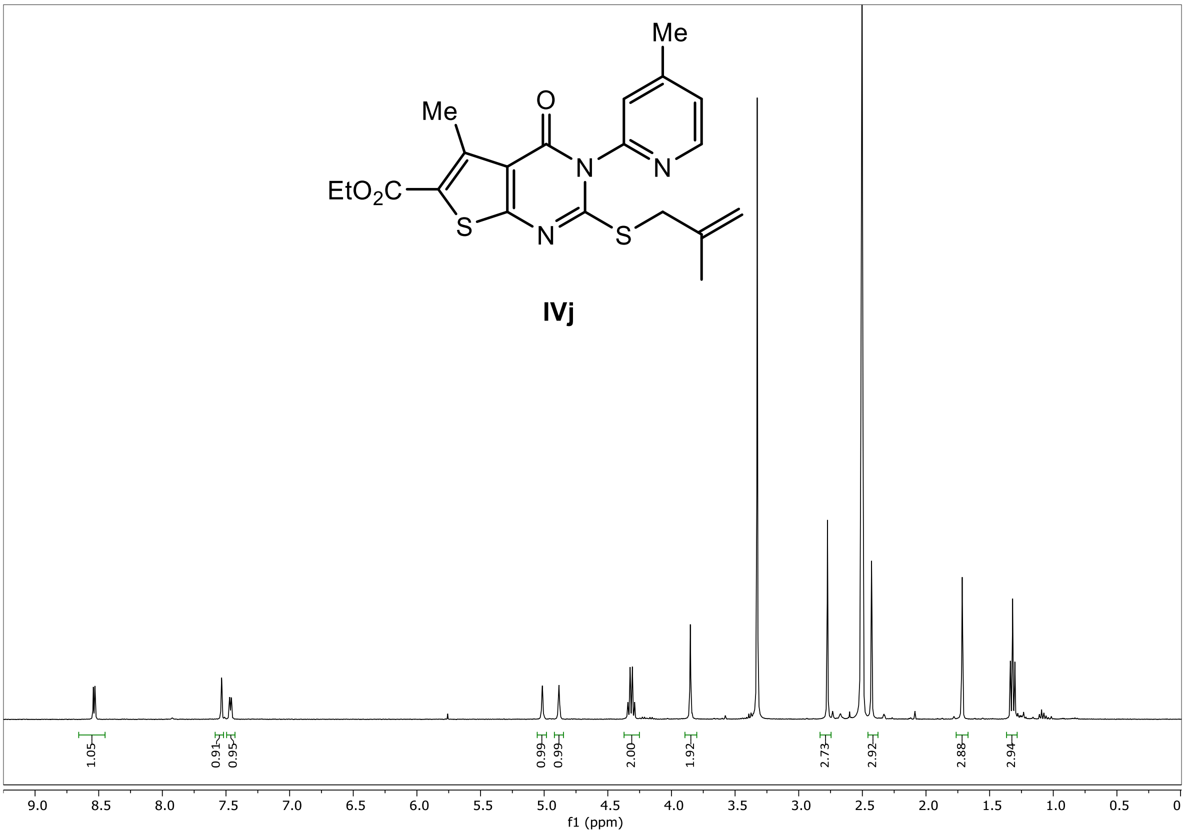

^{1}H-NMR (400 MHz, DMSO-d_6)

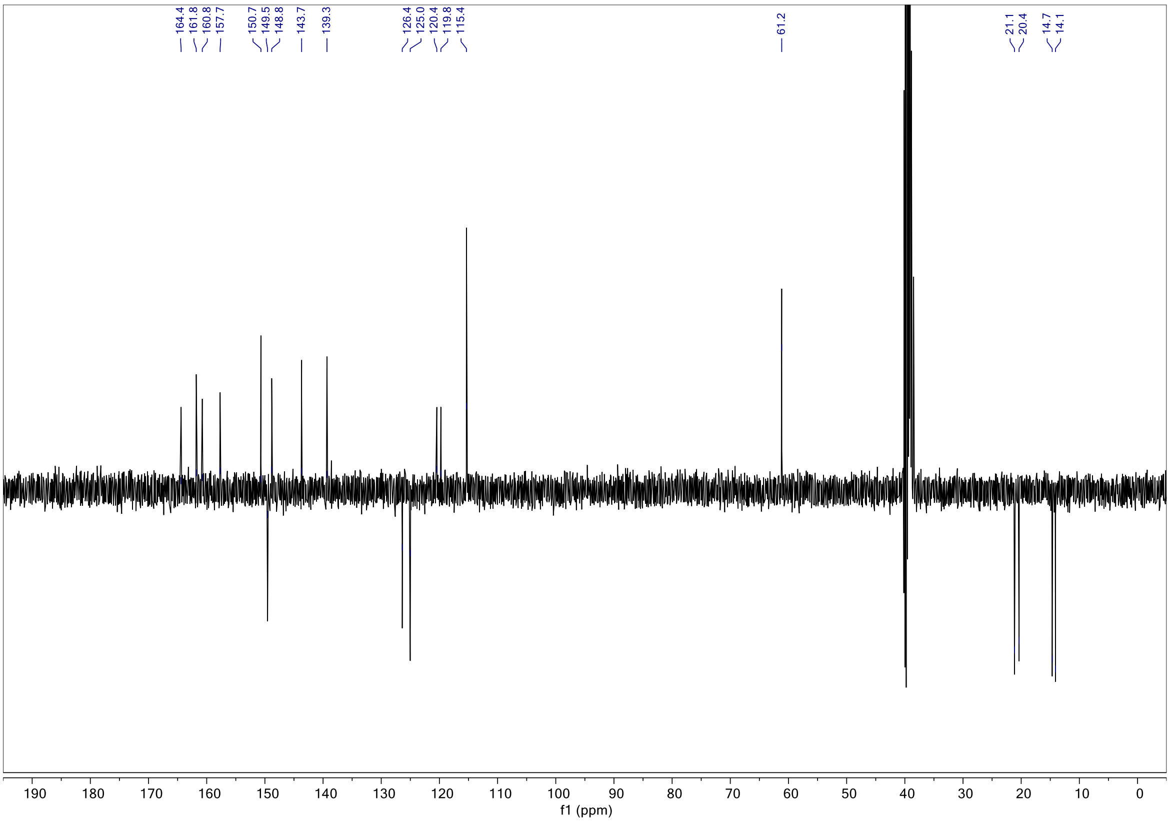

^{13}C{^{1}H}-APT NMR (100 MHz, DMSO-d_6)

Ethyl 2-(allylthio)-5-methyl-3-(4-methylpyridin-2-yl)-4-oxo-3,4-dihydrothieno[2,3-d]pyrimidine-6-carboxylate (IVk)

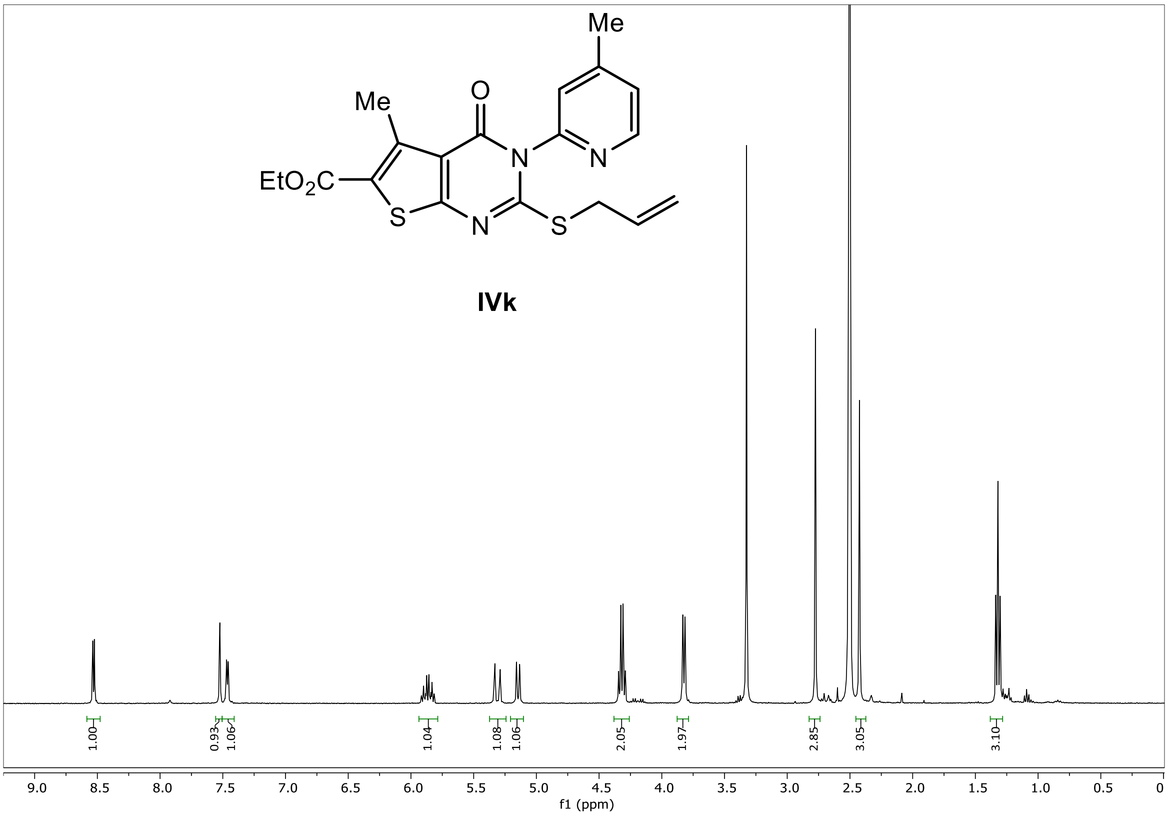

^{1}H-NMR (400 MHz, DMSO-d_6)

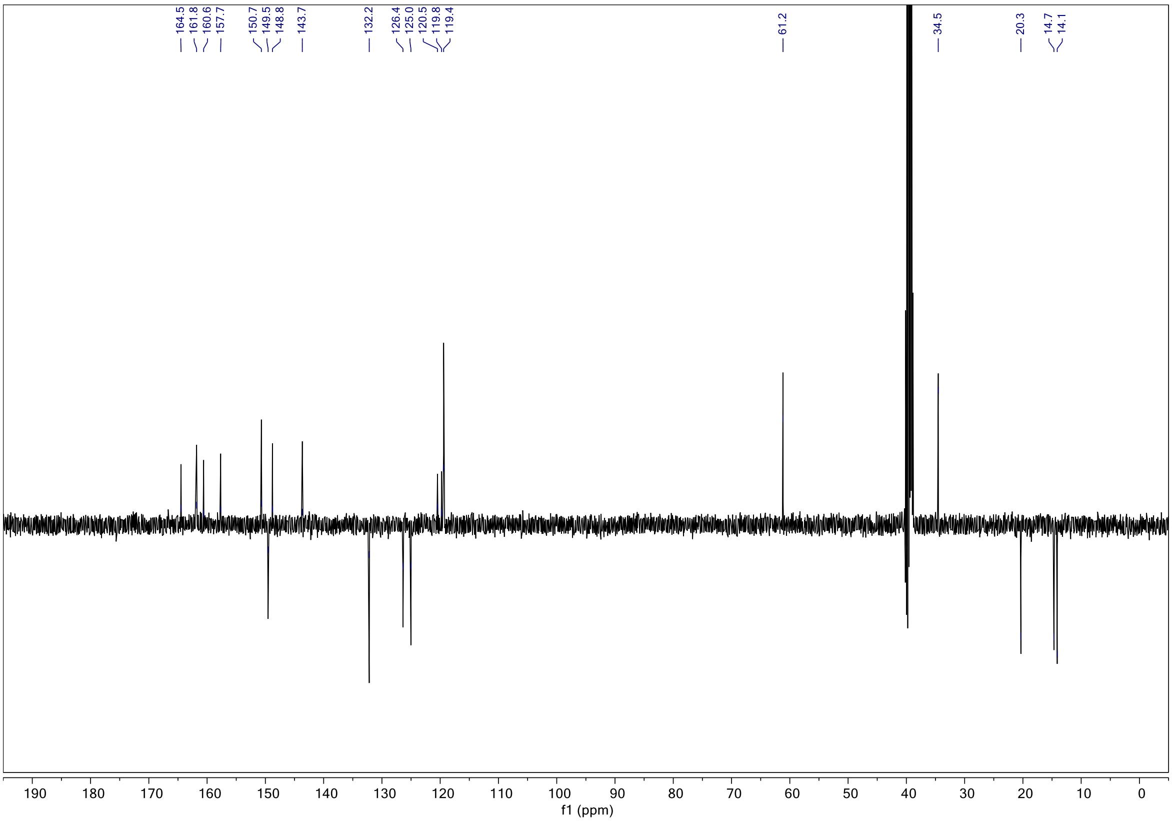

^{13}C{^{1}H}-APT NMR (100 MHz, DMSO-d_6)

2-(Allylthio)-6-(hydroxymethyl)-5-methyl-3-(4-methylpyridin-2-yl)thieno[2,3-d]pyrimidin-4(3H)-one (IVl)

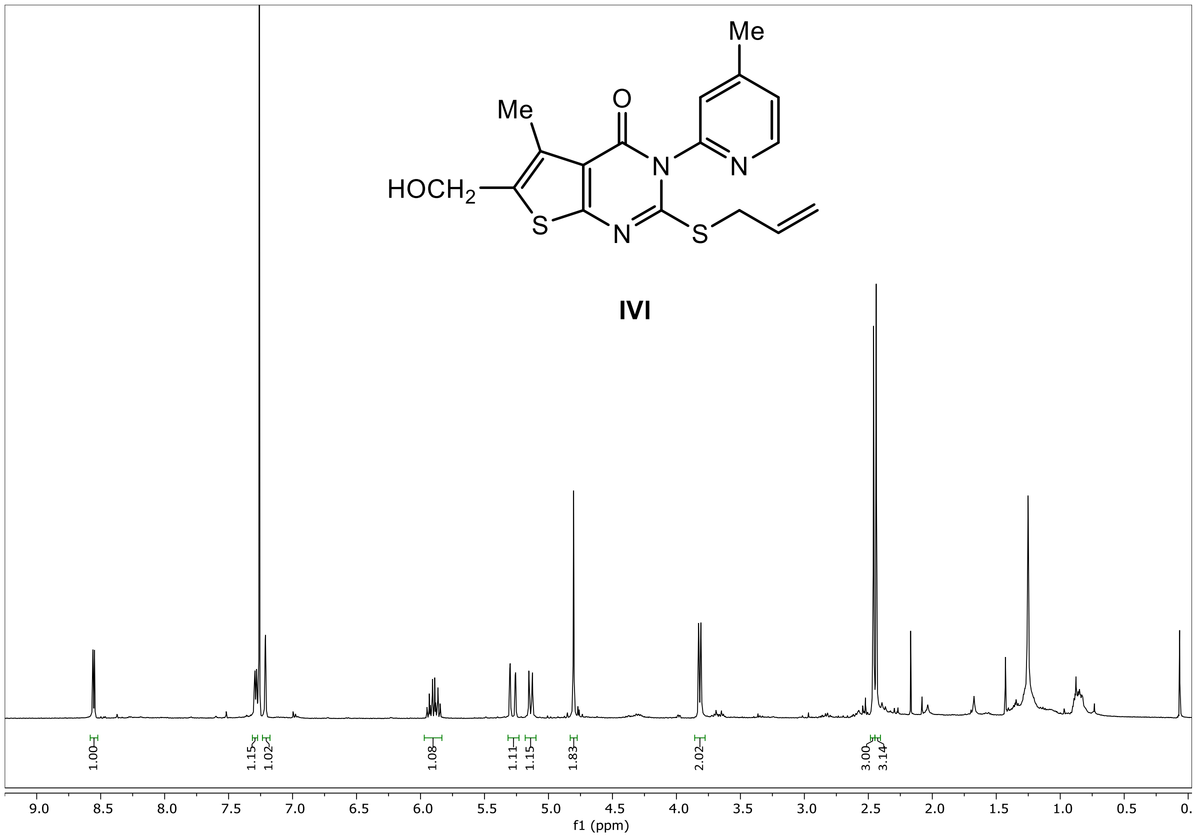

^{1}H-NMR (400 MHz, $CDCl_3$)

3-(3,5-Dimethylphenyl)-5,6-dimethyl-2-thioxo-2,3-dihydrothieno[2,3-d]pyrimidin-4(1H)-one (IVm)

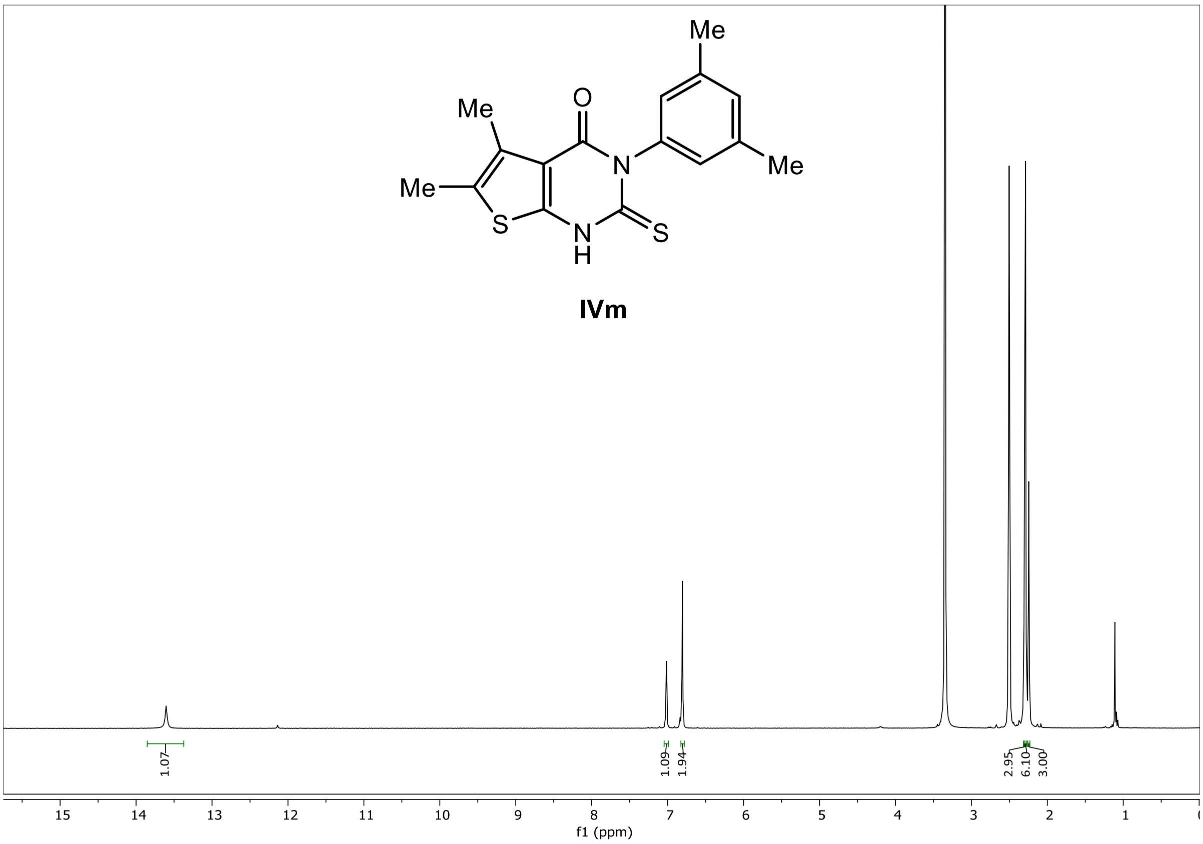

^{1}H-NMR (400 MHz, DMSO-d_6)

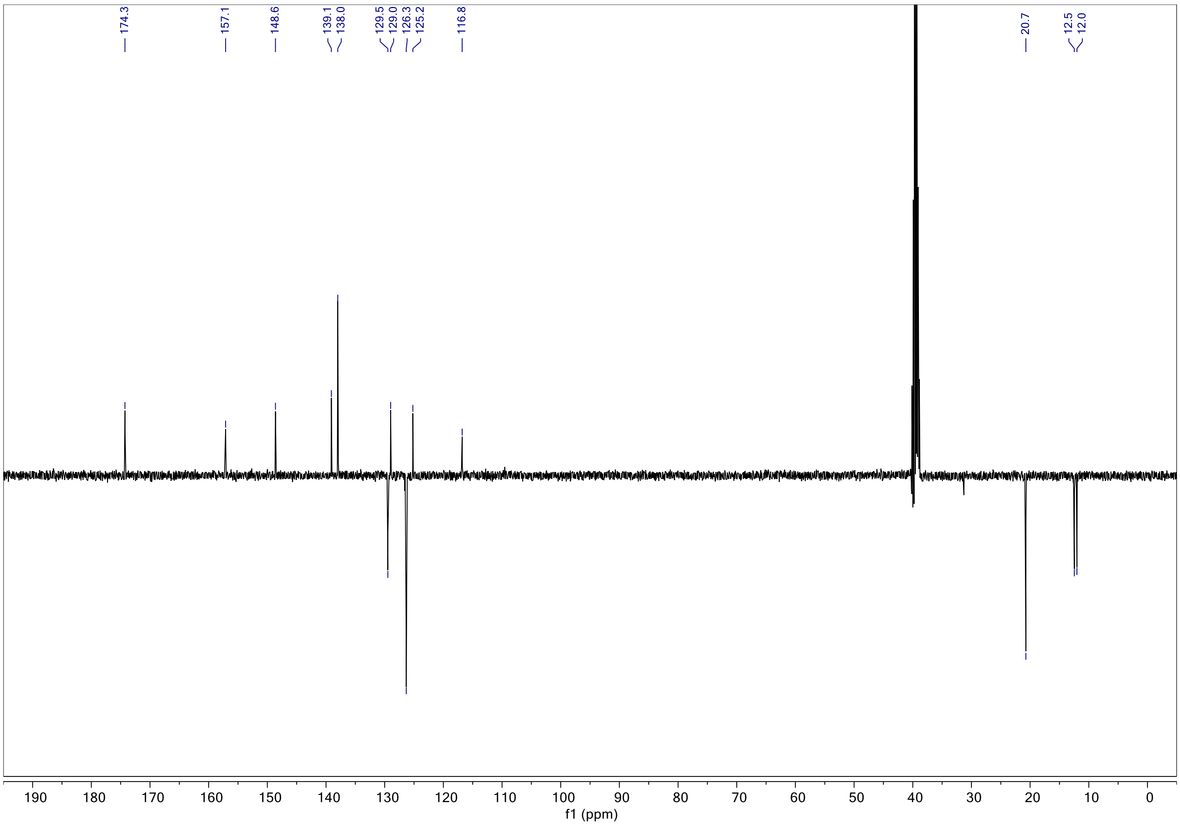

^{13}C{^{1}H}-APT NMR (100 MHz, DMSO-d_6)

5,6-Dimethyl-3-(naphthalen-1-yl)-2-thioxo-2,3-dihydrothieno[2,3-d]pyrimidin-4(1H)-one (IVn)

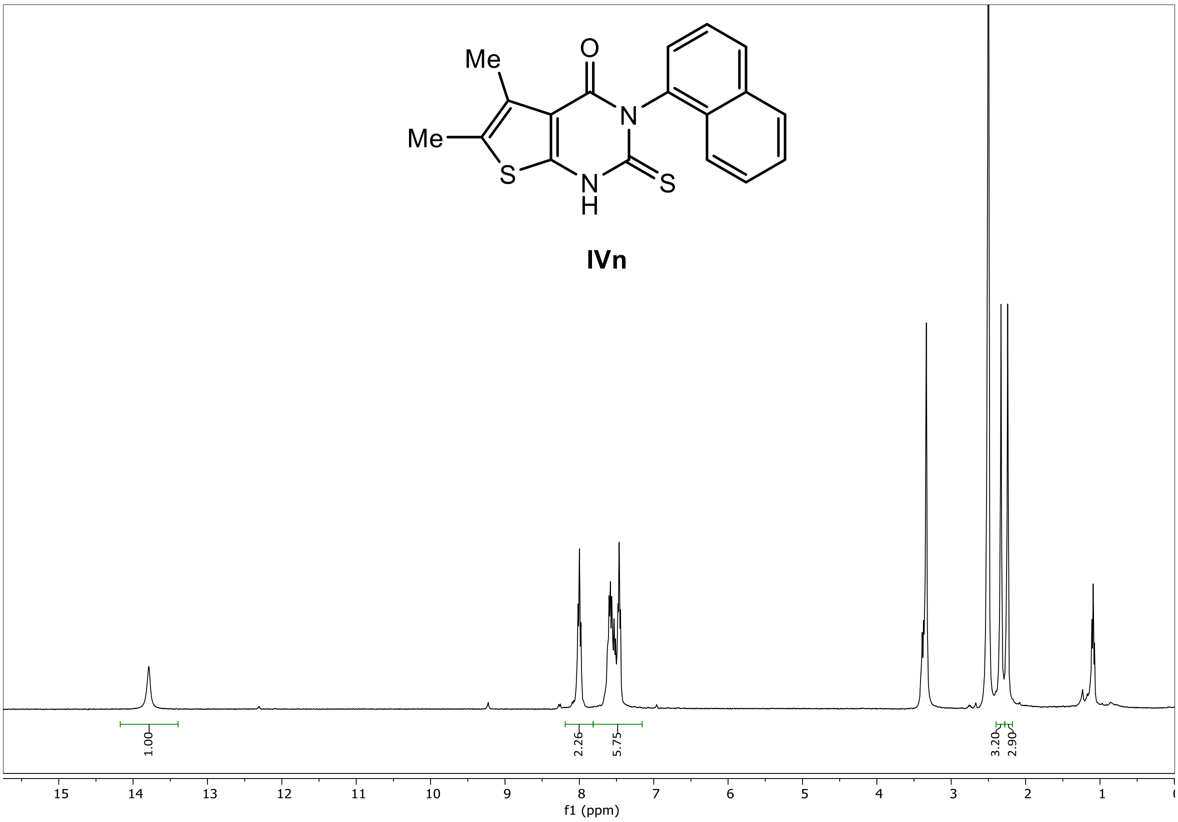

^{1}H-NMR (400 MHz, DMSO-d_6)

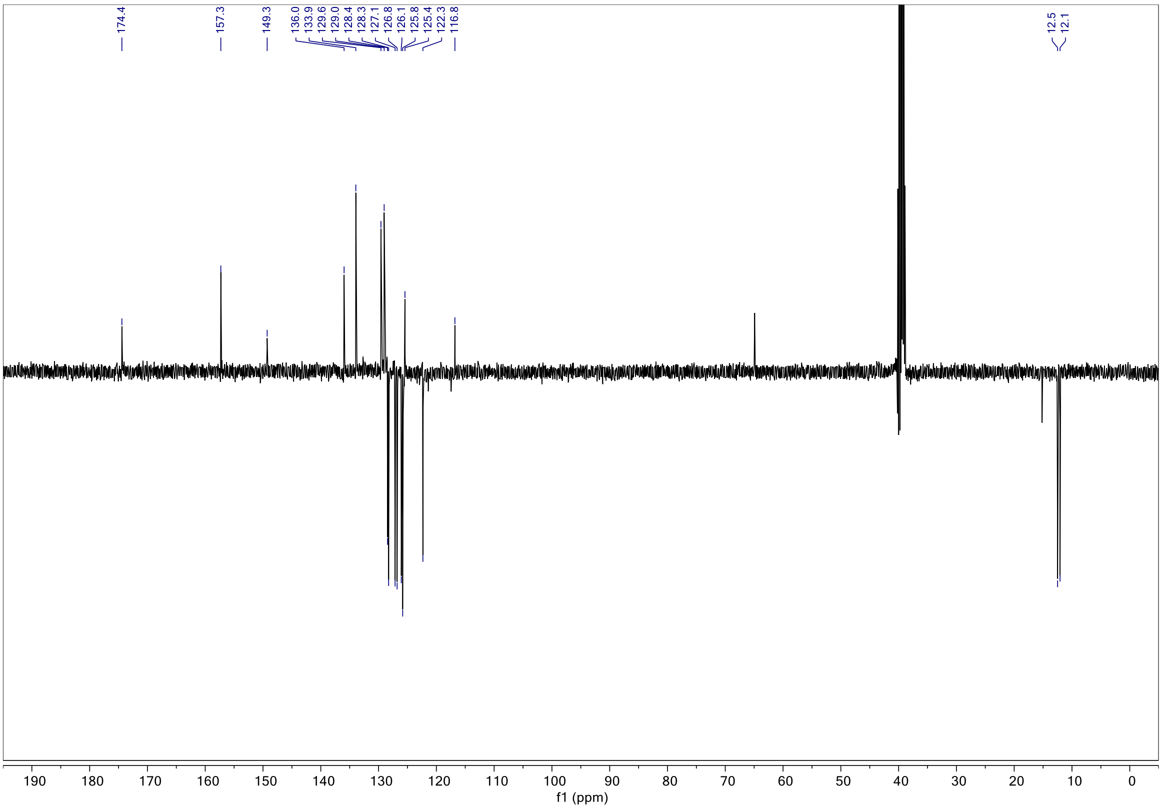

^{13}C{^{1}H}-APT NMR (100 MHz, DMSO-d_6)

5,6-Dimethyl-3-(5,6,7,8-tetrahydronaphthalen-1-yl)-2-thioxo-2,3-dihydrothieno[2,3-d]pyrimidin-4(1H)-one (IVo)

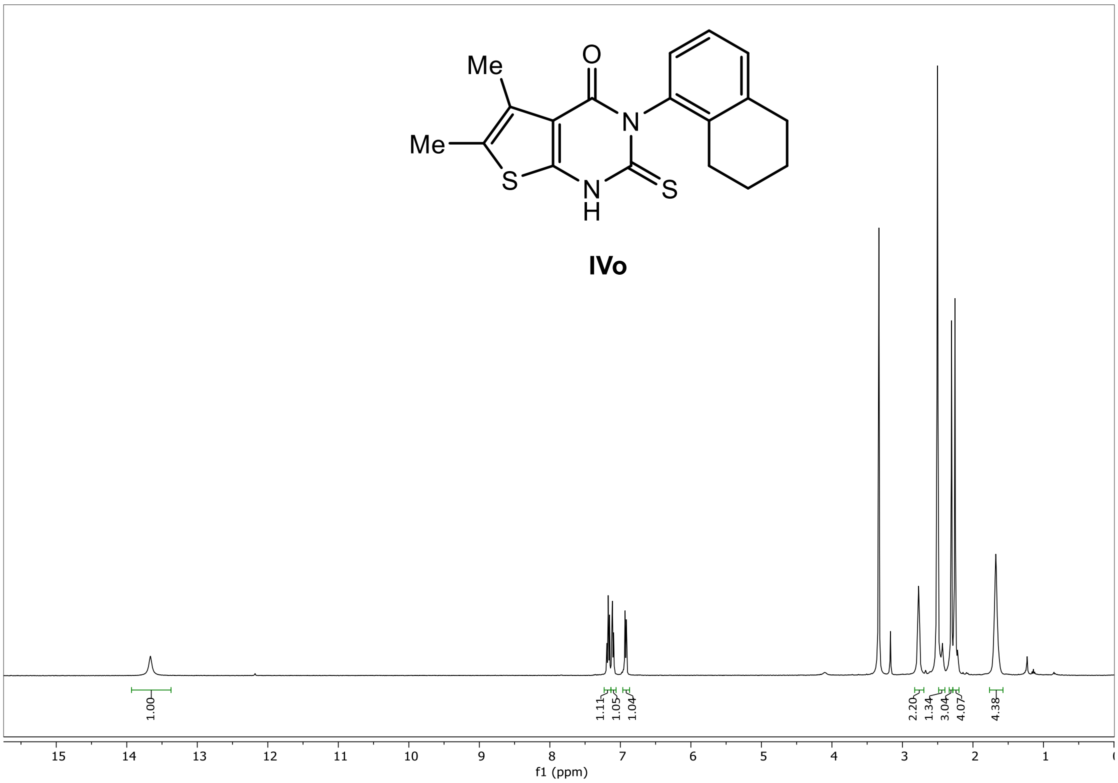

^{1}H-NMR (400 MHz, $CDCl_3$)

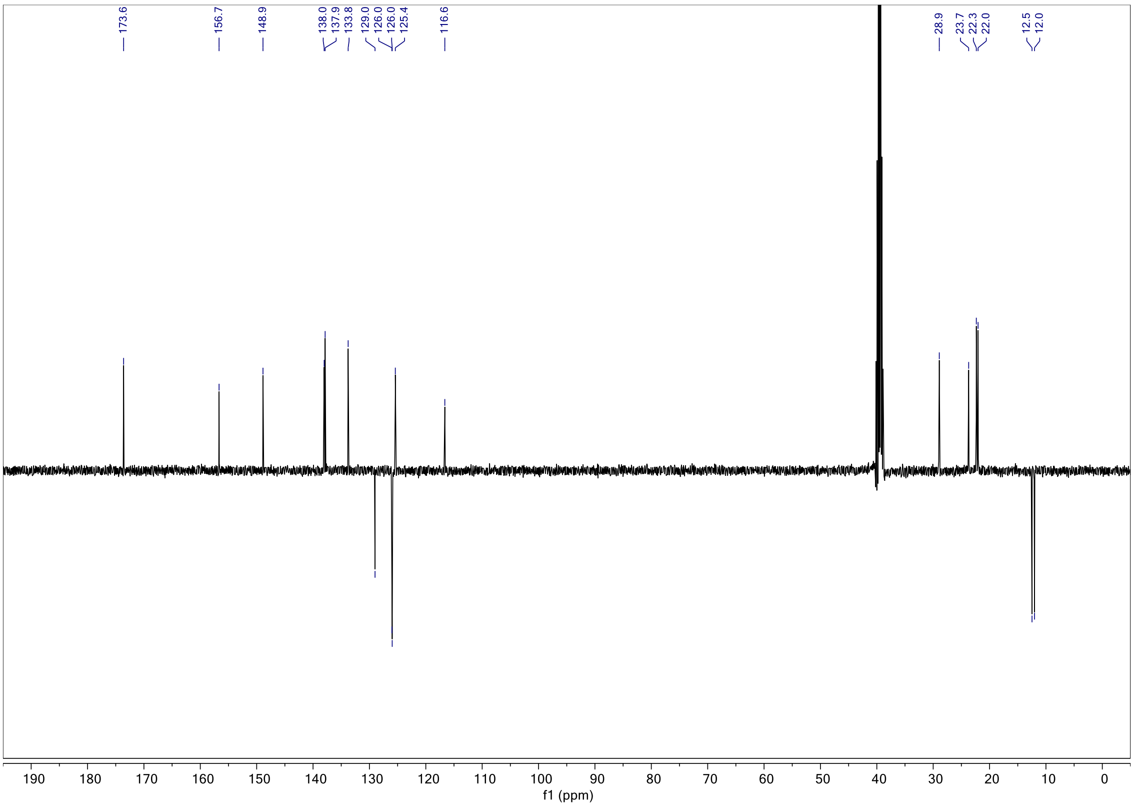

^{13}C{^{1}H}-APT NMR (100 MHz, $CDCl_3$)

2-((5,6-Dimethyl-3-(naphthalen-1-yl)-4-oxo-1,2,3,4-tetrahydrothieno[2,3-d]pyrimidin-2-yl)thio)acetonitrile (IVp)

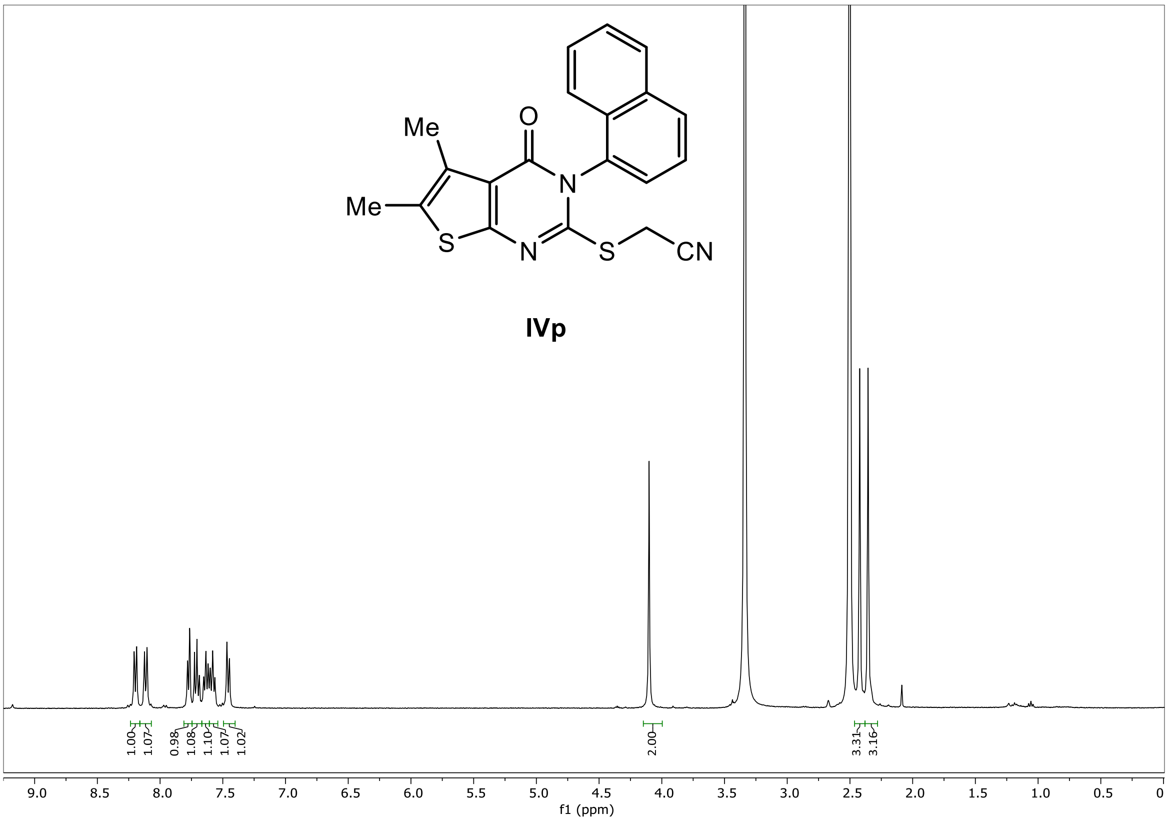

^{1}H-NMR (400 MHz, $CDCl_3$)

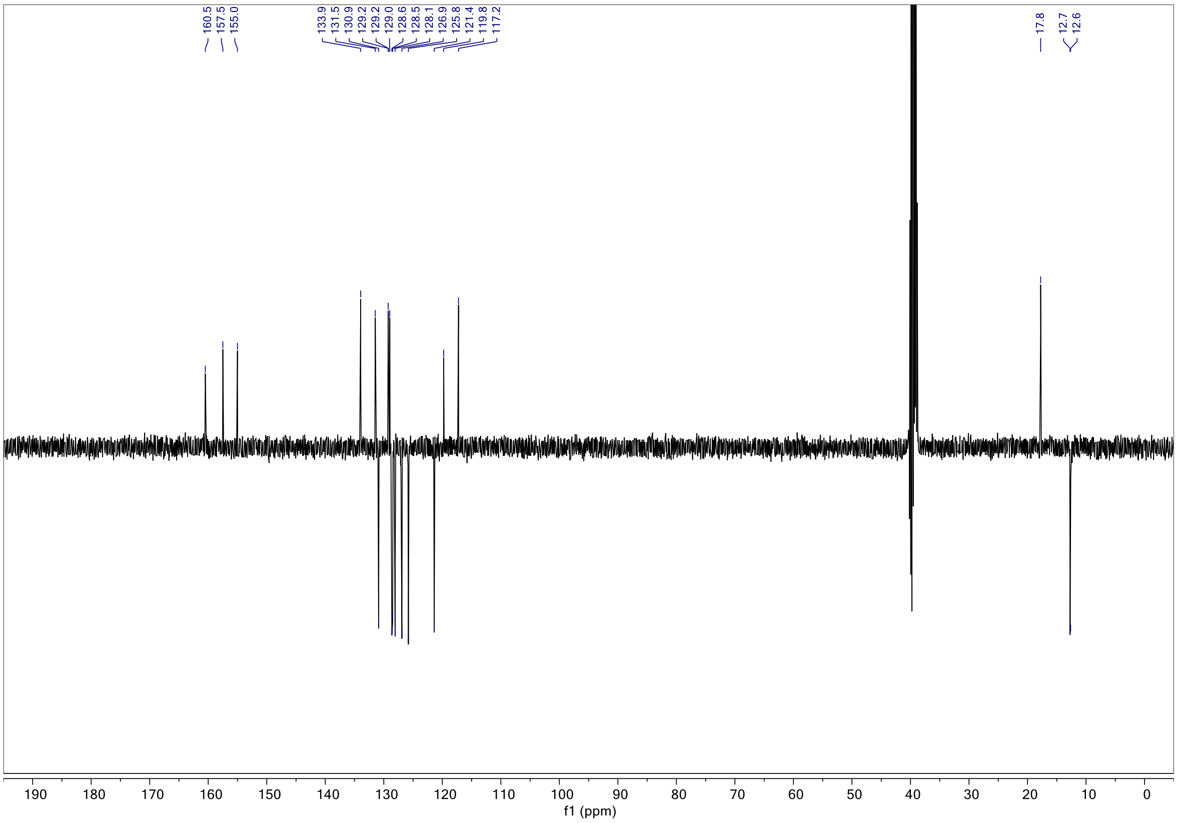

^{13}C{^{1}H}-APT NMR (100 MHz, $CDCl_3$)

Diethyl 2-amino-5-methylthiophene-3,4-dicarboxylate (19)

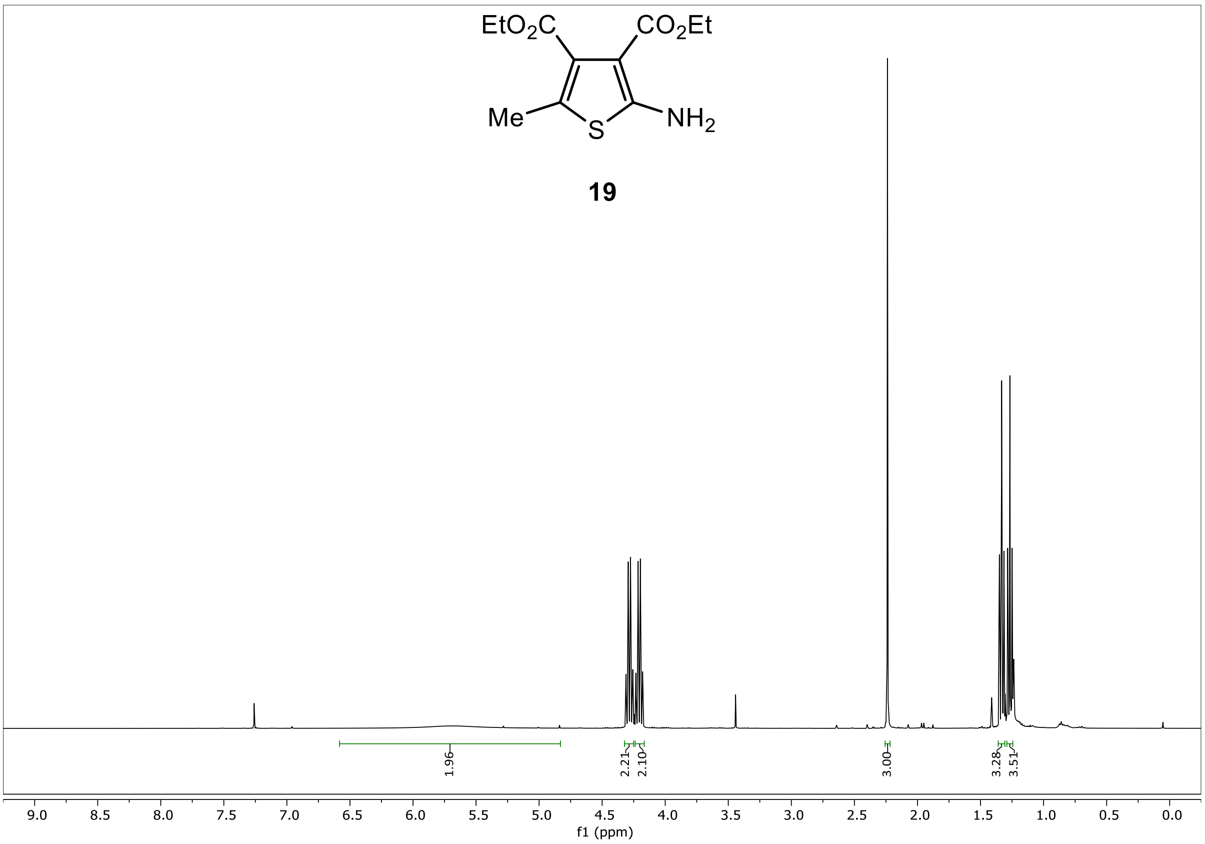

^{1}H-NMR (400 MHz, $CDCl_3$)

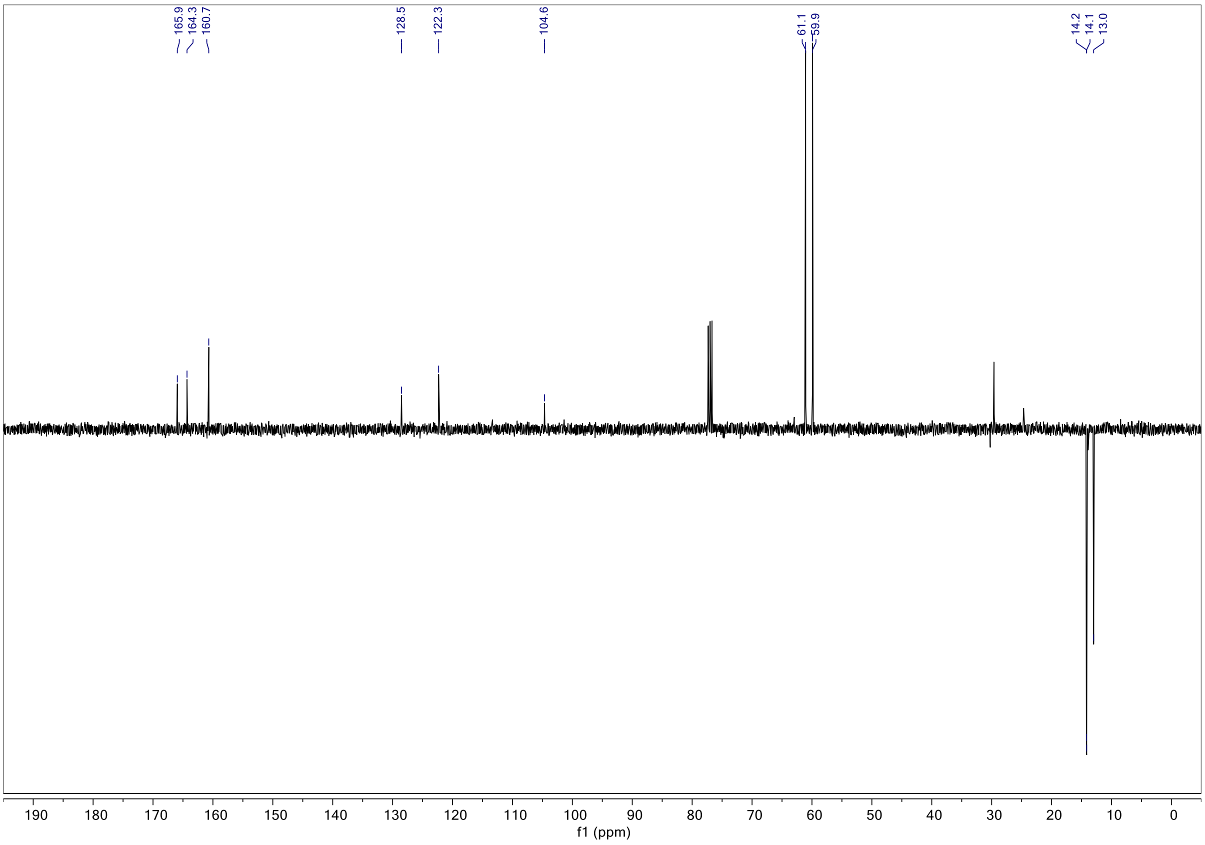

^{13}C{^{1}H}-APT NMR (100 MHz, $CDCl_3$)

Diethyl 2-isothiocyanato-5-methylthiophene-3,4-dicarboxylate (20)

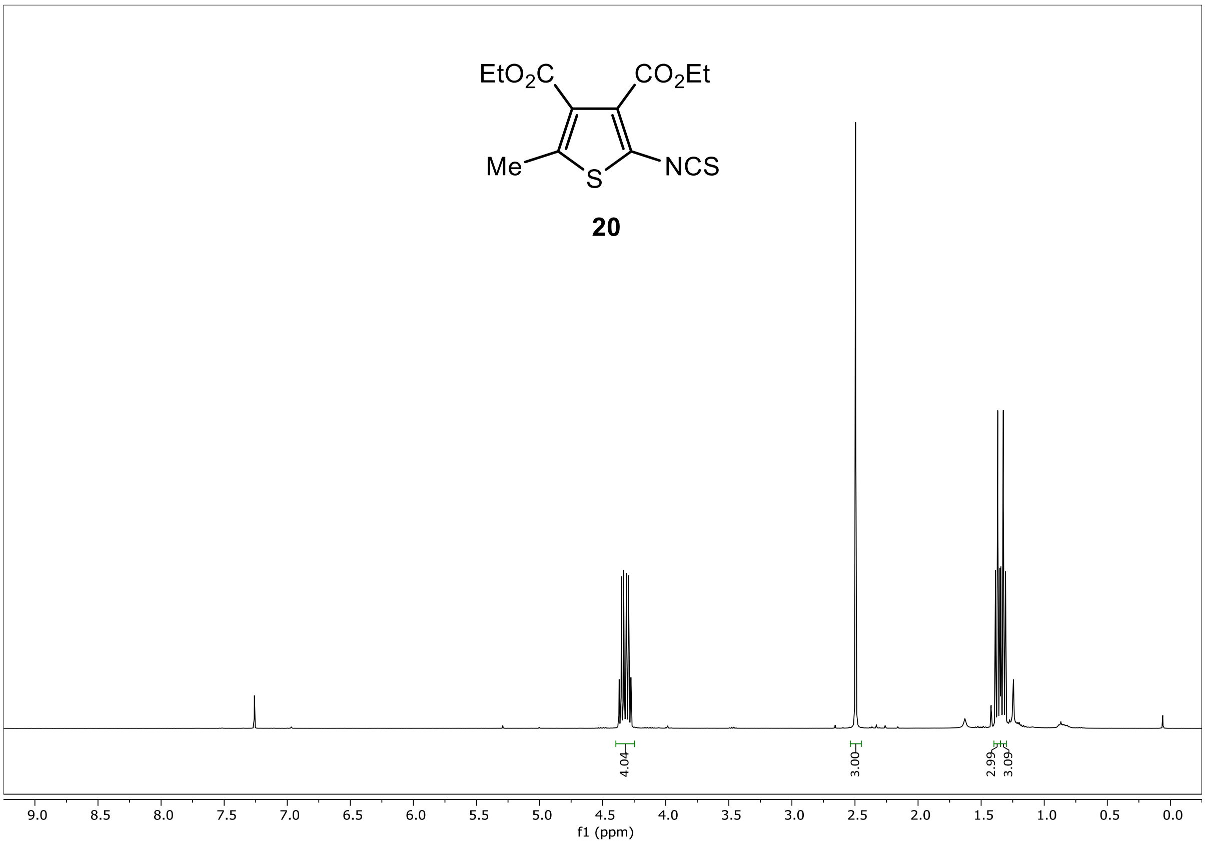

^{1}H-NMR (400 MHz, $CDCl_3$)

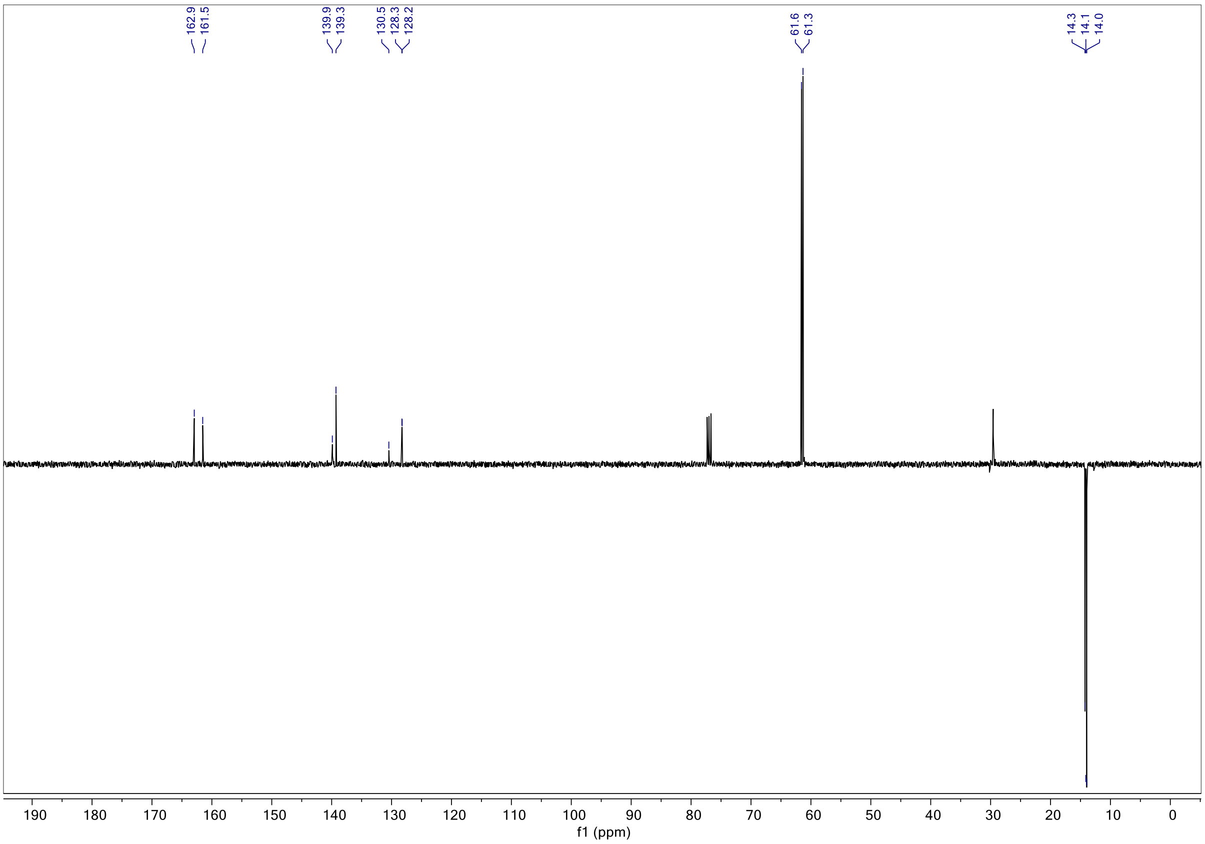

$^{13}C\{^{1}H\}$-APT NMR (100 MHz, $CDCl_3$)

Diethyl 2-methyl-5-(3-(4-methylpyridin-2-yl)thioureido)thiophene-3,4-dicarboxylate (21)

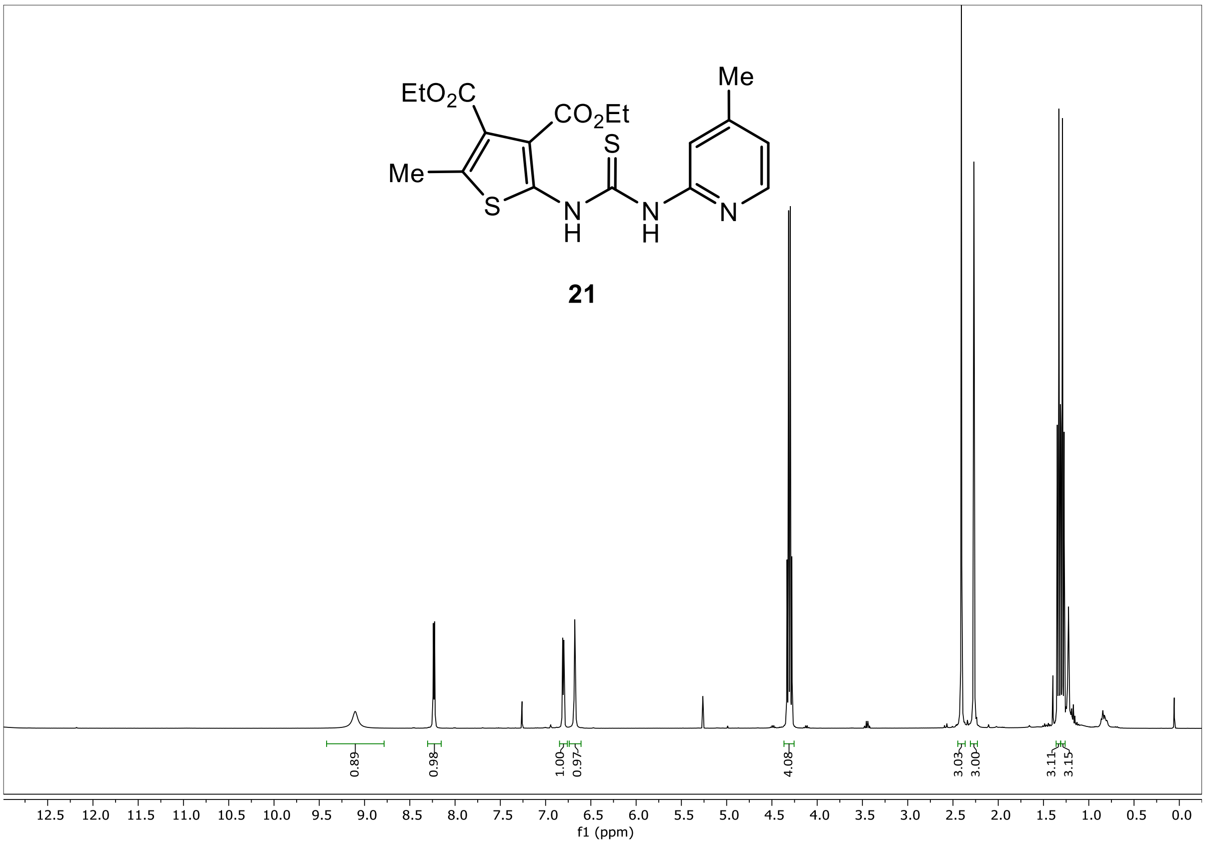

^{1}H-NMR (400 MHz, $CDCl_3$)

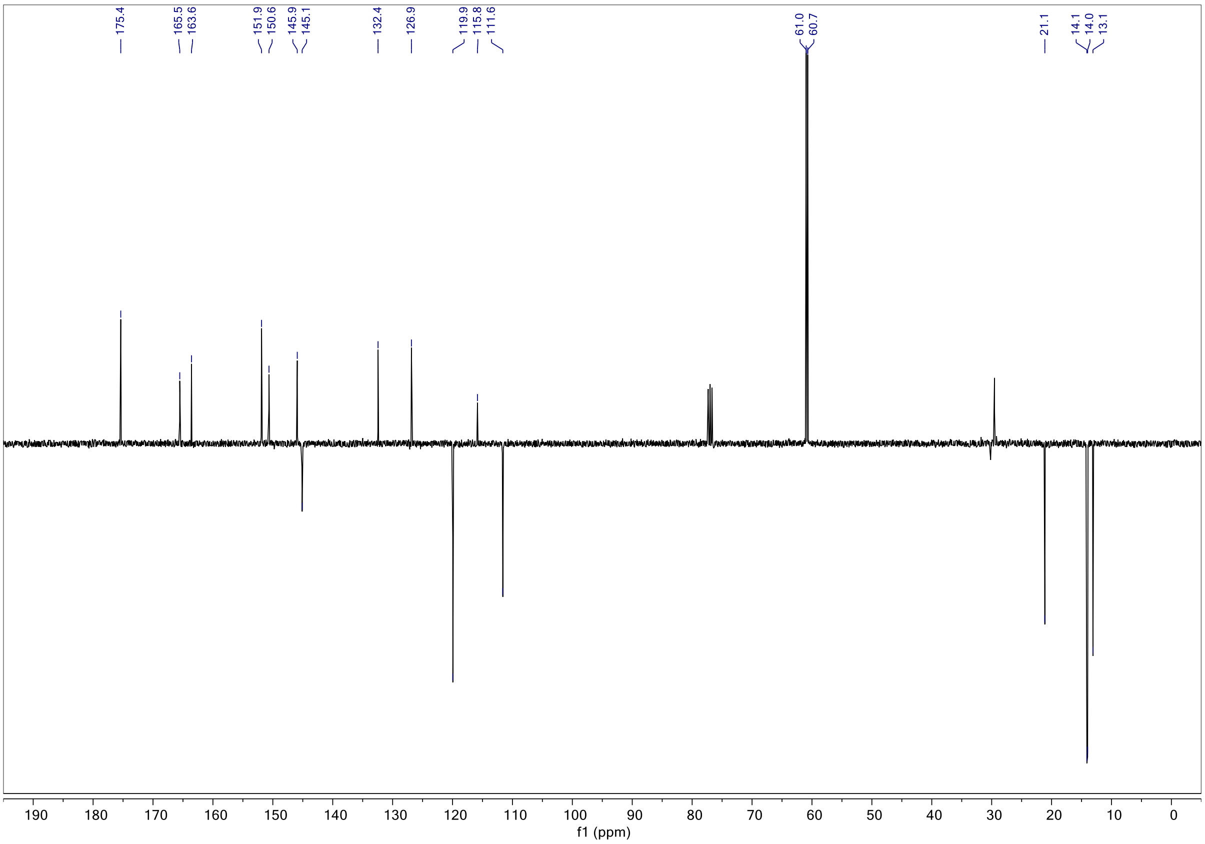

^{13}C{^{1}H}-APT NMR (100 MHz, $CDCl_3$)

Ethyl 6-methyl-3-(4-methylpyridin-2-yl)-4-oxo-2-thioxo-1,2,3,4-tetrahydrothieno[2,3-d]pyrimidine-5-carboxylate (IVq)

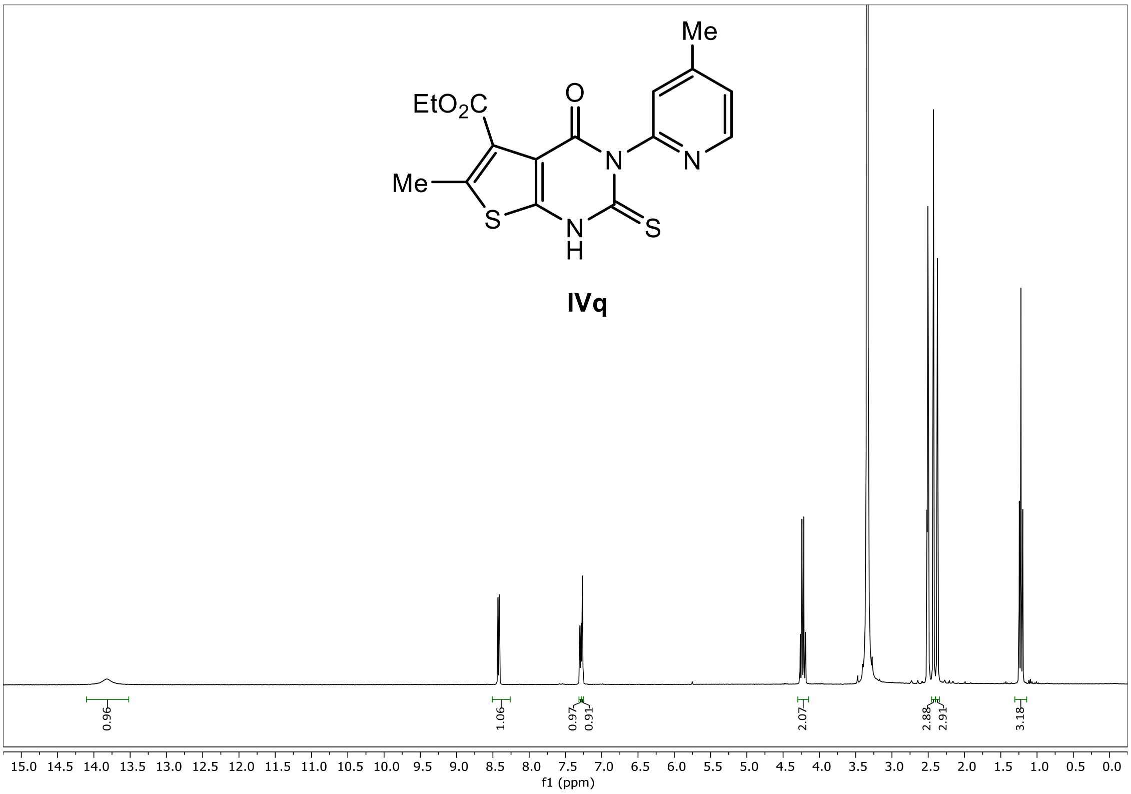

^{1}H-NMR (400 MHz, $CDCl_3$)

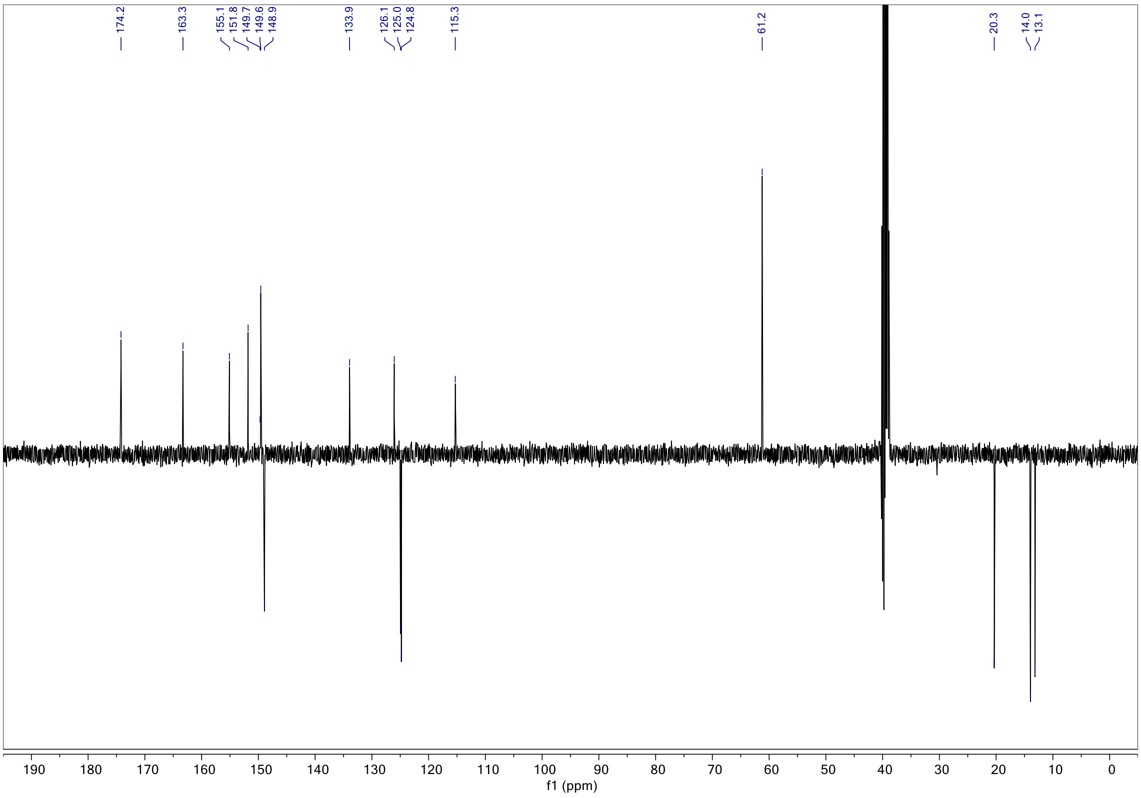

$^{13}C\{^1H\}$-APT NMR (100 MHz, $CDCl_3$)
